# Synthesis of the B-*seco* limonoid core scaffold

**DOI:** 10.3762/bjoc.10.15

**Published:** 2014-01-16

**Authors:** Hanna Bruss, Hannah Schuster, Rémi Martinez, Markus Kaiser, Andrey P Antonchick, Herbert Waldmann

**Affiliations:** 1 Abteilung Chemische Biologie, Max-Planck-Institut für Molekulare Physiologie, Otto-Hahn-Straße 11, 44227 Dortmund, Germany; 2 Fakultät für Chemie und Chemische Biologie, Technische Universität Dortmund, Otto-Hahn-Straße 6, 44227 Dortmund, Germany; 3 Chemical Biology, Zentrum für Medizinische Biotechnologie, Fakultät für Biologie, Universität Duisburg-Essen, Universitätsstraße 2, 45117 Essen, Germany

**Keywords:** B-*seco* limonoids, biology oriented synthesis, Ireland–Claisen rearrangement, natural products, tetranortriterpenoids

## Abstract

Synthetic investigations towards the structurally complex and highly decorated framework of B-*seco* limonoid natural products by means of a [3,3]-sigmatropic rearrangement are described. Detailed model studies reveal, that an Ireland–Claisen rearrangement can be employed to construct the central C9–C10 bond thereby giving access to the B-*seco* limonoid scaffold. However, application of the developed strategy ended up failing in more complex and sterically demanding systems.

## Introduction

**Figure 1:**
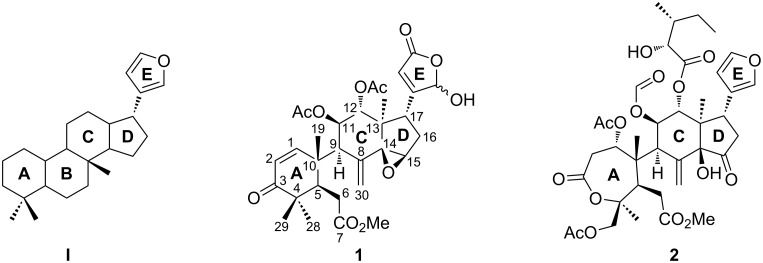
Structures of the 4,4,8-trimethyl-17-furanylsteroid core structure **I** and the representative B-*seco* limonoids 21-hydroxytoonacilide (**1**) and prieurianin (**2**).

B-*seco* limonoids are a family of more than 100 highly oxygenated plant tetranortriterpenoids derived from the 4,4,8-trimethyl-17-furanylsteroid core structure **I** (Figure 1) [1–3]. Members of this natural product class, like 21-hydroxytoonacilide (**1**) [4–5] and prieurianin (**2**) [6–16] display antifeedant [6–7,17–21], antimalaria and anticancer [10–11,22–25] as well as diverse further bioactivities. Recently it was discovered that prieurianin (**2**) impairs the actin cytoskeleton by a mechanism that does not involve direct interaction with actin suggesting that its mode of action differs from previously known modulators [26].

B-*seco* limonoids constitute exceptionally challenging synthesis targets, as the characteristic structural features are a compact, highly oxygenated as well as richly decorated framework and stereochemically dense functionalization. In all B-*seco* limonoids an A ring is linked by a C–C bond to a *trans*-fused bicyclic C–D ring having an *exo*-methylene moiety. The crowded C9–C10 bond bridging the two domains is the main synthetic obstacle.

**Scheme 1:**
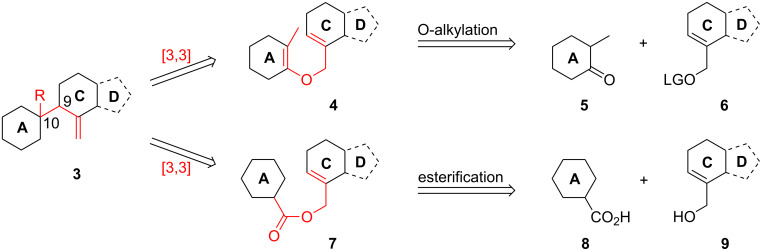
Retrosynthetic analysis of the B-*seco* limonoid framework employing a [3,3]-sigmatropic rearrangement for formation of the C9–C10 bond. R = Me or CO_2_H, LG = leaving group.

Taking into account the biology-oriented synthesis (BIOS) concept [27–34], which employs the scaffolds of biologically relevant natural product classes to inspire the synthesis of probes and reagents for chemical biology and medicinal chemistry research, we aimed at the development of a synthetic strategy to get access to the B-*seco* limonoid scaffold by means of a [3,3]-sigmatropic rearrangement as key step enabling the formation of the crucial C9–C10 bond (Scheme 1) [35]. In this paper we present a full report on this synthesis [36] as well as further synthetic studies towards the application of the developed strategy to the total synthesis of B-*seco* limonoid natural products.

## Results and Discussion

**Scheme 2:**
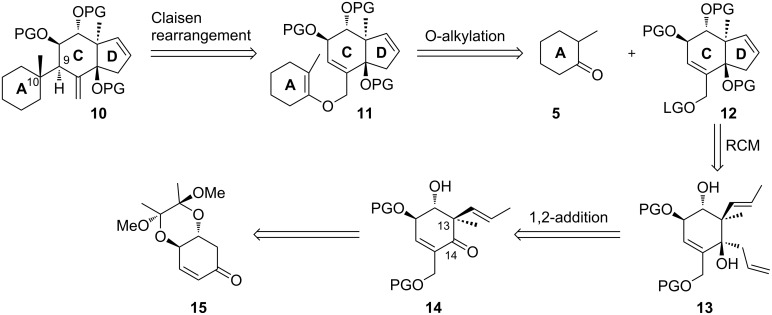
Retrosynthetic analysis of the B-*seco* limonoid scaffold employing a Claisen rearrangement as key step for formation of the C9–C10 bond. PG = protecting group, LG = leaving group.

**Retrosynthetic analysis: Claisen rearrangement.** In planning the synthesis we were inspired by Ley’s synthesis of azadirachtin in which a Claisen rearrangement has been successfully employed as key transformation [37–38]. Thus the allyl vinyl ether rearrangement precursor **11** was thought to be obtained from an O-alkylation between the thermodynamic enolate of 2-methylcyclohexanone (**5**) and the bicyclic electrophile **12** (Scheme 2). A challenging synthetic problem appears to be the construction of the stereochemically dense *trans*-fused C–D ring system **12**, which possesses four stereogenic centers including two contiguous asymmetric quaternary centers at the ring junction. We decided to start the sequence with known enone **15** [39] and intended to construct the all-carbon quaternary center at C13 by substrate controlled α-functionalization. The second quaternary center at C14 might be established by 1,2-addition and finally, ring-closing metathesis would give rise to bicyclic system **12**.

**Scheme 3:**
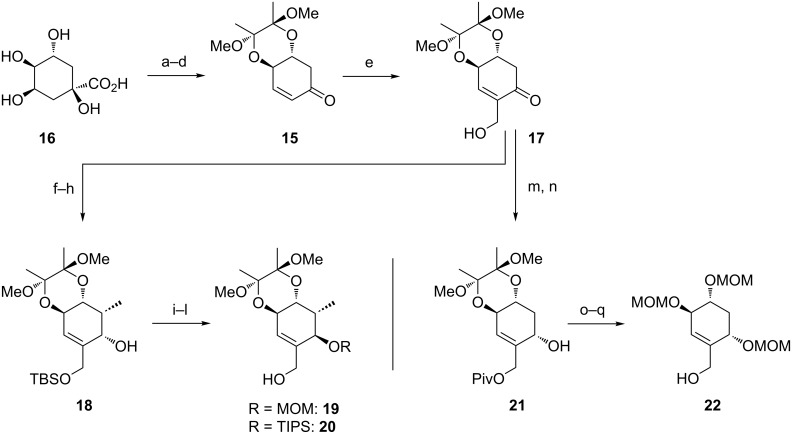
Synthesis of alcohols **19**, **20** and **22**. Reagents and conditions: a) CSA, 2,3-butanedione, trimethyl orthoformate, MeOH, reflux, 16 h, 93%; b) NaBH_4_, MeOH, rt, 0.5 h, quant.; c) silica-gel supported NaIO_4_, CH_2_Cl_2_/MeOH (20:1), rt, 3 h, quant.; d) MsCl, NEt_3_, CH_2_Cl_2_, 0 °C to rt, 3 h, quant.; e) paraformaldehyde, imidazole, THF/1 M NaHCO_3_ (1:1), rt, 2.5 h, 74%; f) TBSOTf, 2,6-lutidine, CH_2_Cl_2_, 0 °C, 15 min, quant.; g) LiHMDS, MeI, THF/DMPU (5:1), −78 °C to −10 °C, 91%, de = 100%; h) NaBH_4_, CeCl_3_·7H_2_O, MeOH, 0 °C, 15 min, 90%, de = 100%; i) PPh_3_, *p*-nitrobenzoic acid, DEAD, toluene, rt, 18 h; j) MeOH, Et_2_O, aqueous saturated K_2_CO_3_ solution, rt, 1 h, 64% (2 steps); for the synthesis of **19**: k) DIPEA, MOMCl, CH_2_Cl_2_, reflux, 16 h, 99%; l) TBAF, THF, rt, 20 min, 94%; for the synthesis of **20**: k) TIPSOTf, 2,6-lutidine, DMF, 0 °C to rt, 3 h, quant.; l) PTSA, MeOH, THF, rt, 30 h, 77%; m) PivCl, DMAP, pyridine, −15 °C to rt, 2 h, 77%; n) NaBH_4_, CeCl_3_·7H_2_O, MeOH, 0 °C to rt, 30 min, 96%, de = 100%; o) TFA, H_2_O, rt, 5 min, 72%; p) DIPEA, MOMCl, CH_2_Cl_2_, reflux, 15 h, 62%; q) DIBAL-H, CH_2_Cl_2_, −78 °C to rt, 2 h, quant.

**Model studies towards the Claisen rearrangement.** As the stereochemical substitution on the C ring system will have a major impact on the face selectivity of the planned Claisen rearrangement we defined precursors **19**, **20** and **22** (Scheme 3) as suitable model systems, presenting appropriate stereogenic substitution at the C ring system. After merging these alcohols with an undecorated A ring, the resulting allyl vinyl ethers could serve as suitable rearrangement precursors.

The synthesis of **19** and **20** commenced with enone **15** [39], which was prepared from (−)-quinic acid (**16**) according to the route reported by Arthurs et al. [40] with minor modifications. After Baylis–Hillman reaction and subsequent silylation of the resulting free primary hydroxy group, substrate-controlled α-methylation of the lithium enolate proceeded with full stereocontrol [41–42], which can be explained by the strong conformational rigidity of the butane-2,3-diacetal (BDA) protected *trans*-diequatorial diols [43] and the stereoelectronic preference for axial attack on the electron-rich C13. Luche reduction with stereoelectronically preferred axial attack of the hydride gave alcohol **18** and Mitsunobu reaction installed the required stereochemistry at C14. The free C14 hydroxy group was masked with protecting groups (MOM and TIPS) of different size and chemical nature to examine the *face*-selectivity of the [3,3]-sigmatropic rearrangement. After selective desilylation, alcohols **19** and **20** were obtained.

In order to synthesize a model substrate without the rigid BDA-protecting group, compound **21** was treated with aqueous TFA to give a triol which was masked with three MOM-protecting groups (Scheme 3). Reductive cleavage of the pivaloyl group furnished alcohol **22**.

Mesylation or tosylation of the primary alcohols in **19**, **20** and **22** gave suitable electrophiles for the planned O-alkylation with the thermodynamic enolate of 2-methylcyclohexanone. However, under various conditions (NaH/15-crown-5/THF; *t-*BuOK/18-crown-6/THF/DMPU; KHMDS/THF) [44–45], the intended O-alkylation to yield the allyl vinyl ether failed. Equally, copper-catalyzed C–O couplings [46] of the alcohols **19**, **20** and **22** with organotrifluoroborates failed or gave only low yields. Likewise Buchwald’s procedure for the copper-catalyzed coupling of primary alcohols with vinyl iodides to yield the allyl vinyl ether or, depending on the reaction conditions, directly the Claisen rearrangement products was not successful [47].

**Scheme 4:**
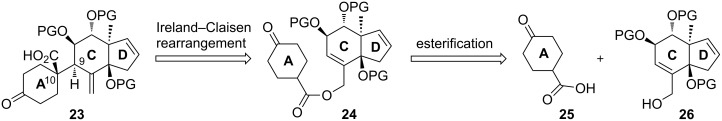
Retrosynthetic analysis of the B-*seco* limonoid scaffold employing an Ireland–Claisen rearrangement as the key step for the formation of the C9–C10 bond. PG = protecting group.

**Alternative strategy: Ireland–Claisen rearrangement.** As the Claisen rearrangement precursor, the allyl vinyl ether, could not be obtained under various conditions, we had to change the synthetic strategy and employed the Ireland variant of the Claisen rearrangement to construct the crucial C9–C10 bond (Scheme 4). In this strategy the A-ring **25** could be merged with the bicyclic C–D system **26** by esterification in order to obtain the allyl ester rearrangement precursor **24**. Murai et al. [48] showed the utility of an Ireland–Claisen rearrangement in their model studies addressing the limonoid framework of azadirachtin.

**Scheme 5:**
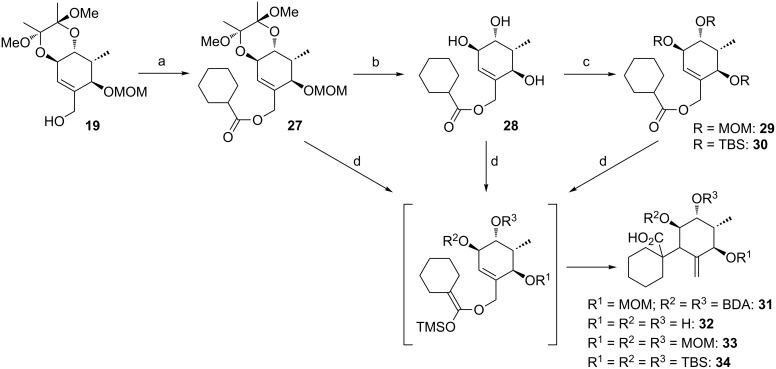
Synthesis and Ireland–Claisen rearrangement of the allyl esters **27**, **28**, **29** and **30**. Reagents and conditions: a) DCC, DMAP, cyclohexanecarboxylic acid, CH_2_Cl_2_, rt, 4 h; b) TFA/H_2_O (3:1), rt, 30 min, 73% (2 steps); for the synthesis of **29**: c) DIPEA, MOMCl, CH_2_Cl_2_, 40 °C, 15 h, 86%; for the synthesis of **30**: c) imidazole, TBSCl, CH_2_Cl_2_, rt, 21 h, 45%; d) KHMDS, TMSCl, toluene, −78 °C to 85 °C, 18 h. Results: see Table 1.

**Model studies towards the Ireland–Claisen rearrangement.** To test the feasibility of this approach, model rearrangement precursor **27** was prepared from alcohol **19** (Scheme 5). Esterification with cyclohexanecarboxylic acid gave the desired allyl ester **27**. Furthermore, to investigate the influence of the protecting groups at C11, C12 and C14 on the stereoselectivity and reaction rate, the allyl esters **28**, **29** and **30** were prepared.

The four rearrangement precursors **27**, **28**, **29** and **30** were exposed to KHMDS and TMSCl in toluene, in order to induce the Ireland–Claisen rearrangement (Scheme 5, Table 1). The rigid BDA protecting group at C11 and C12 in **27** was not compatible with these conditions, resulting in only cleavage of the ester moiety (Table 1, entry 1). In case of the unprotected rearrangement precursor **28** an excess of base and TMSCl was used for in situ protection of the three free hydroxy groups as TMS ether, but the desired [3,3]-sigmatropic rearrangement could not be induced (Table 1, entry 2). However rearrangement of *tris*-MOM ether **29** as well as *tris*-TBS ether **30** gave the desired carboxylic acids **33** and **34** in high yield and excellent diastereoselectivity (Table 1, entries 3 and 4).

**Table 1:** Ireland–Claisen rearrangement of model compounds **27**, **28**, **29** and **30**.

entry	rearrangement precursor	result

1	**27**	cleavage of the ester moiety
2	**28**	no conversion
3	**29**	89% yield of **33** (de = 76%)
4	**30**	quant. yield of **34** (de = 100%)

**Figure 2:**
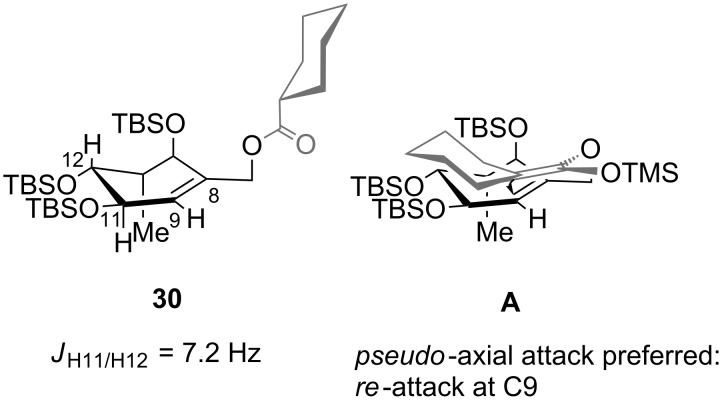
Conformation of rearrangement precursor **30** and possible transition state involved in the Ireland–Claisen rearrangement.

In view of the stereochemistry of the major diastereomers of the products, the rearrangement would occur from the *re-*face (transition state **A**, Figure 2). Thus, assuming the OTBS group in **30** at C14 is pseudo-axial to avoid allylic A^1,2^-strain, the sigmatropic rearrangement occurred via a pseudo-axial attack of the silyl ketene acetal on the double bond in the cyclohexene ring. These results are in accordance with the observations of Ireland et al. [49], who examined the propensity for axial versus equatorial attack in the rearrangement of cyclohexenyl acetates and observed a strong preference for the stereochemically controlled axial approach. The size of the protecting groups had a strong influence on the face-selectivity, furnishing in case of the TBS derivative only one diastereomer. By substitution of C14 with a bulky group (1,2-allylic strain) the ring inversion barrier of the cyclohexene conformations might be increased, resulting in higher de values.

These model studies suggested that an Ireland–Claisen rearrangement is a feasible strategy to construct the C9–C10 bond in B-*seco* limonoids.

**Scheme 6:**
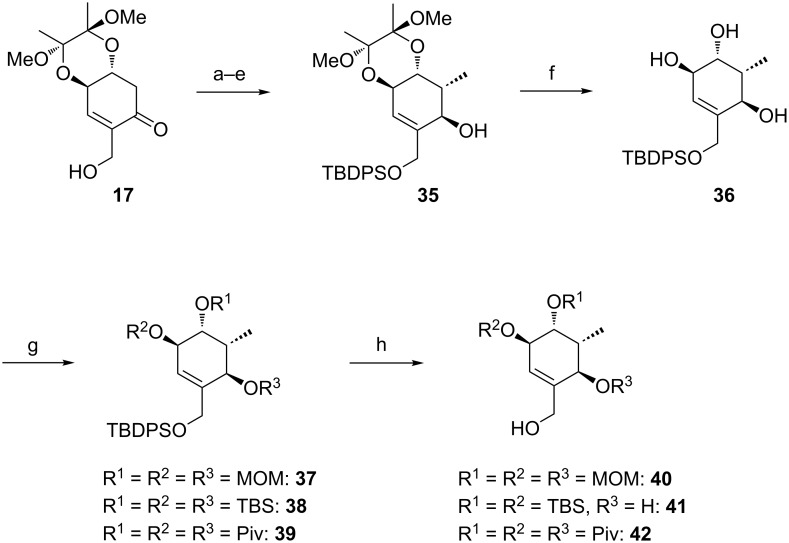
Synthesis of model C rings **40**, **41** and **42**. Reagents and conditions: a) TBDPSCl, DMAP, NEt_3_, CH_2_Cl_2_, rt, 18 h, 93%; b) LiHMDS, MeI, THF/DMPU (10:1), −78 °C to 0 °C, 1.5 h, 98%; c) CeCl_3_·7H_2_O, NaBH_4_, MeOH, 0 °C, 15 min, 86%; d) Ph_3_P, *p-*nitrobenzoic acid, DEAD, toluene, 15 h; e) MeOH, Et_2_O, aqueous saturated K_2_CO_3_-solution, rt, 2 h, 85% (2 steps); f) CH_2_Cl_2_/TFA/H_2_O (2:1:0.1), rt, 10 min, 85%; for the synthesis of **37**: g) DIPEA, MOMCl, NaI, THF, 65 °C, 4.5 h, 98%; for the synthesis of **38** g) TBSCl, imidazole, DMF, rt, 18 h, 92%; for the synthesis of **39**: PivCl, DMAP, pyridine, rt, 4 d, 78%; for the synthesis of **40**: h) **37**, TBAF, THF, rt, 2 h, 95%; for the synthesis of **41**: h) **38**, 10% NaOH/MeOH, reflux, 6.5 h, 72%; for the synthesis of **42**: h) **39**, HF·pyridine, THF, rt, 24 h, 71%.

In order to perform further model studies, we established a straight forward synthetic access to model substrates without the BDA group (Scheme 6). With the TBDPS- instead of the TBS ether on the primary hydroxy group, the BDA group in alcohol **35** could be selectively cleaved by treatment with TFA in aqueous CH_2_Cl_2_ to release triol **36**, which was masked with different protecting groups (MOM, TBS, Piv). After desilylation, the liberated alcohols **40**, **41** and **42** could be esterified with various cyclic and acyclic model A rings to give the targeted rearrangement precursors **A** (Scheme 6, Table 2).

**Table 2:** Esterification of alcohols **40**, **41** and **42** and Ireland–Claisen rearrangement. Reagents and conditions: a) EDC·HCl, DMAP, carboxylic acid, CH_2_Cl_2_, rt, 15–22 h; for **52**: silylation of the free hydroxy group at C14: imidazole, TBSCl, DMAP, DMF, rt, 40 h; b) KHMDS, TMSCl, toluene, −78 °C to 85 °C, ca. 18 h.

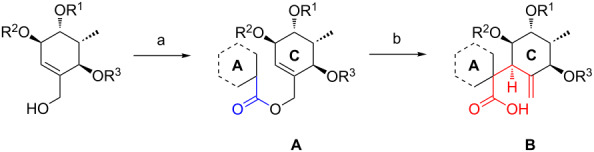			

entry	alcohol	allyl ester **A**	carboxylic acid **B**

1	**40**	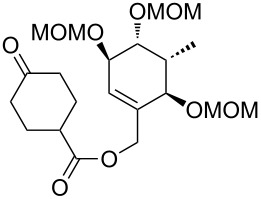 **43** (87% yield)	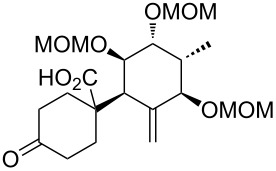 **44** (75% yield, de = 80%)
2	**40**	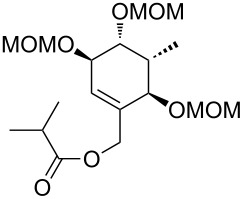 **45** (81% yield)	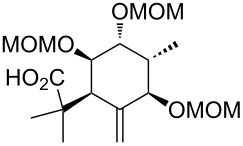 **46** (93% yield, de = 78%)
3	**40**	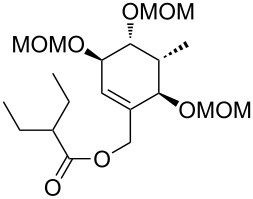 **47** (93% yield)	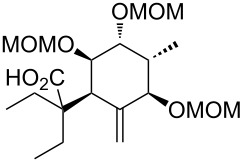 **48** (quant. yield, de = 72%)
4	**40**	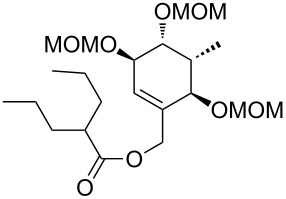 **49** (quant. yield)	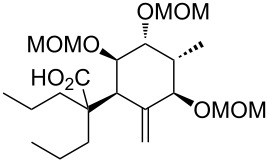 **50** (95% yield, de = 72%)
5	**41**	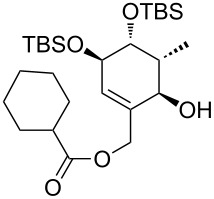 **51** (79% yield)	–
6	**41**	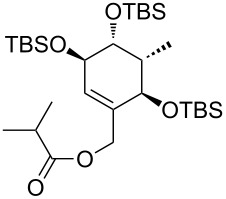 **52** (78% yield, 2 steps)	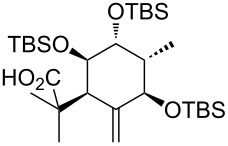 **53** (quant. yield, de = 94%)
7	**42**	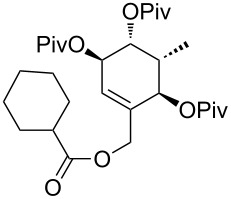 **54** (95% yield)	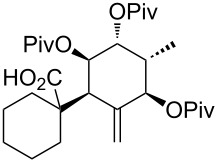 **55** (90% yield, de = 94%)

The obtained rearrangement precursors were submitted to the Ireland–Claisen rearrangement conditions and gave the desired carboxylic acids **B** in excellent yield and diastereoselectivity (Scheme 6, Table 2). The presence of the keto functionality in **43** is compatible with the rearrangement conditions (Table 2, entry 1). Intermediary, the silyl enol ether and the silyl ketene acetal are formed. However, after the rearrangement, the keto-functionality can be set free again during an acidic work-up. In terms of yield and diastereoselectivity there was no difference observed between rearrangements with derivatives with a cyclic or acyclic model A ring. Rearrangement precursor **51** containing a free hydroxy group could not be converted into the desired carboxylic acid (Table 2, entry 5). Under the same conditions but by using an excess of base and TMSCl for the in situ protection of the free hydroxy group, only the starting material could be reisolated.

**Scheme 7:**
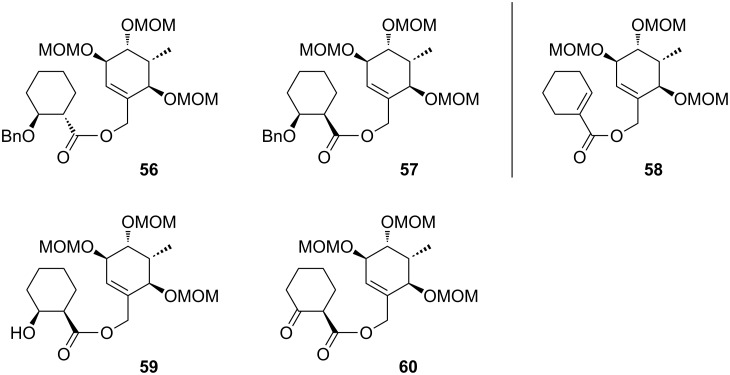
β-Substituted allyl esters tested in the Ireland–Claisen and the Carroll rearrangement.

Encouraged by these findings we attempted to perform the [3,3]-sigmatropic rearrangement with C1-substituted A rings, as many B-*seco* limonoids are oxygenated at this position. For this purpose, both the *anti*- and *syn*-substituted β-alkoxy esters **56** and **57** were synthesized (Scheme 7; for experimental procedure see Supporting Information File 1). In the literature [50] not many examples are known in which β-alkoxy esters serve as rearrangement precursors as the β-elimination of the alkoxy group can easily occur under the rearrangement conditions. Indeed, under various conditions by using different bases (LDA, LiHMDS, KHMDS) and solvents (THF, toluene, THF/DMPU) and by carefully controlling the reaction conditions (deprotonation at low temperature (−100 °C), gradual warming of the reaction mixture), only the elimination product **58** could be isolated.

Furthermore, a dianionic Ireland–Claisen rearrangement employing β-hydroxy ester **59** was unsuccessful (Scheme 7). Under various conditions, we observed only decomposition of the starting material by cleavage of the ester moiety. Likewise, cleavage of the ester moiety occurred in an attempt to perform a Carroll rearrangement with β-keto ester **60**.

These studies show that it might be necessary to oxygenate at C1 after the sigmatropic rearrangement because the C1 substitution seems to have major impact on the success of the rearrangement.

**Scheme 8:**
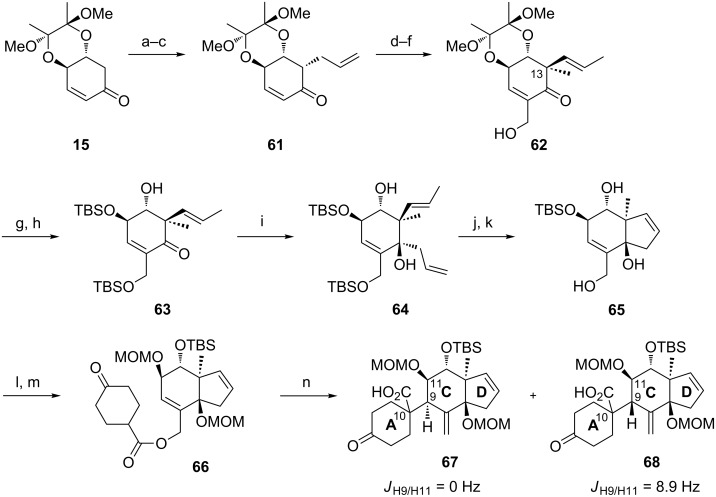
Synthesis and Ireland–Claisen rearrangement of bicyclic allyl ester precursor **66**. Reagents and conditions: a) LiHMDS, TMSCl, THF, −78 °C to 0 °C, 90 min; b) NBS, THF, 0 °C, 90 min, 68% (2 steps); c) allyltributyltin, AIBN, toluene, 80 °C, 18 h, 81%, de = 80%; d) KHMDS, THF, −78 °C, 1 h, then MeI, rt, 20 min, 74%, de = 100%; e) PdCl_2_(CH_3_CN)_2_, toluene, 65 °C, 2 days, 70% (81% brsm); f) paraformaldehyde, imidazole, THF/1 M NaHCO_3_ (2:1), rt, 19 h, 74%; g) TFA/H_2_O (3:1), rt, 30 min, 84%; h) TBSCl, imidazole, DMF, rt, 18 h, 94%; i) tetraallyltin, BuLi, THF, −78 °C to rt, 30 min, 72%, dr = ca. 2:1; j) Grubbs I, CH_2_Cl_2_, rt, 18 h, 80%; k) HF·pyridine, THF, 0 °C to rt, 20 h, 99%; l) EDC·HCl, 4-oxocyclohexanecarboxylic acid (**25**), DMAP, CH_2_Cl_2_, rt, 16 h, 80%; m) DIPEA, MOMCl, NaI, THF, 50 °C, 21 h, 62%; n) LiHMDS, supernatant of a centrifuged mixture of TMSCl/NEt_3_ (v/v = 1:1) and toluene, toluene, −78 °C to 65 °C in 6 h, 60 h at 65 °C, 88% yield, dr = 1:2 (**67**:**68**).

**Synthesis of the B-*seco* limonoid scaffold.** Encouraged by the results of the rearrangements of the model substrates with an undecorated A ring, we attempted to access the B-*seco* limonoid scaffold by an analogous rearrangement of bicyclic precursor **66** (Scheme 8).

The elaboration of the required bicyclic system commenced with enone **15** (Scheme 8). Initially, we envisaged an α-vinylation via Buchwald’s procedure for the catalytic asymmetric vinylation of enones [51]. However, the desired vinylated product could not be obtained under the described conditions. An alternative α-formylation/Wittig olefination sequence gave only low yields. O’Brien et al. [41] described the failure of a direct alkylation of the lithium enolate of **15** with alkyl halides under several conditions. They incorporated an α-allyl side chain via an α-bromo-enone, which can be obtained from an initially formed silyl enol ether, and subsequent reaction with NBS. Keck allylation of the α-bromo-enone using allyltributyltin and AIBN gave the desired α-allylated product **61**. We used the high substrate control to construct the first quaternary center at C13 by trapping the potassium enolate of **61** with MeI, furnishing the desired product as a single diastereomer. Isomerization of the terminal double bond with cat. PdCl_2_(CH_3_CN)_2_ and Baylis–Hillman reaction proceeded uneventfully to afford compound **62**. Deprotection of the butane-2,3-diacetal under acidic conditions followed by selective silylation of the primary and the allylic hydroxy groups gave alcohol **63**.

The second quaternary center of the bicyclic C–D system **66** was envisaged to be constructed by 1,2-addition, using the free β-hydroxy functionality in **63** as directing group. Several conditions with allyl boronates, stannanes, silanes, indium, magnesium bromide, cerium, zinc bromide and other reagents have been screened. Finally, the best result was achieved with tetraallyltin and BuLi affording a 2:1 mixture of diastereomers, with the desired diastereomer being the minor product. The stereochemistry was unambiguously confirmed by crystal structure analysis of a derivative of the major diastereomer [36]. Thus, the preference for the axial attack predominates the aimed directing effect of the β-hydroxy group.

Bicyclic system **65** could be obtained by ring-closing metathesis using Grubbs 1^st^ generation catalyst and subsequent selective deprotection of the primary silyl ether. After esterification with 4-oxocyclohexanecarboxylic acid (**25**) and protection of the remaining two free hydroxy groups as MOM ethers, which was accompanied by silyl migration, the synthesis of rearrangement precursor **66** was completed.

Application of the developed reaction conditions for the rearrangement of the model systems was fruitless, resulting in only cleavage of the ester moiety. Crucial for the success of the envisaged Ireland–Claisen rearrangement was a gradual warming of the reaction mixture from −78 °C to 65 °C over a period of 6 h and the addition of the supernatant of a centrifuged mixture of TMSCl, NEt_3_ and toluene instead of the addition of unactivated TMSCl. When **66** was exposed to TMSCl/NEt_3_ and LiHMDS in toluene, the intended rearrangement occurred smoothly giving rise to **67** and **68** (ca. 1:2 ratio, 88% combined yield) through the desired C9–C10 bond formation (Scheme 8). The configuration of the diastereomers was determined based on the analysis of the coupling constants of H9 and H11 and nOe signal enhancements.

**Figure 3:**
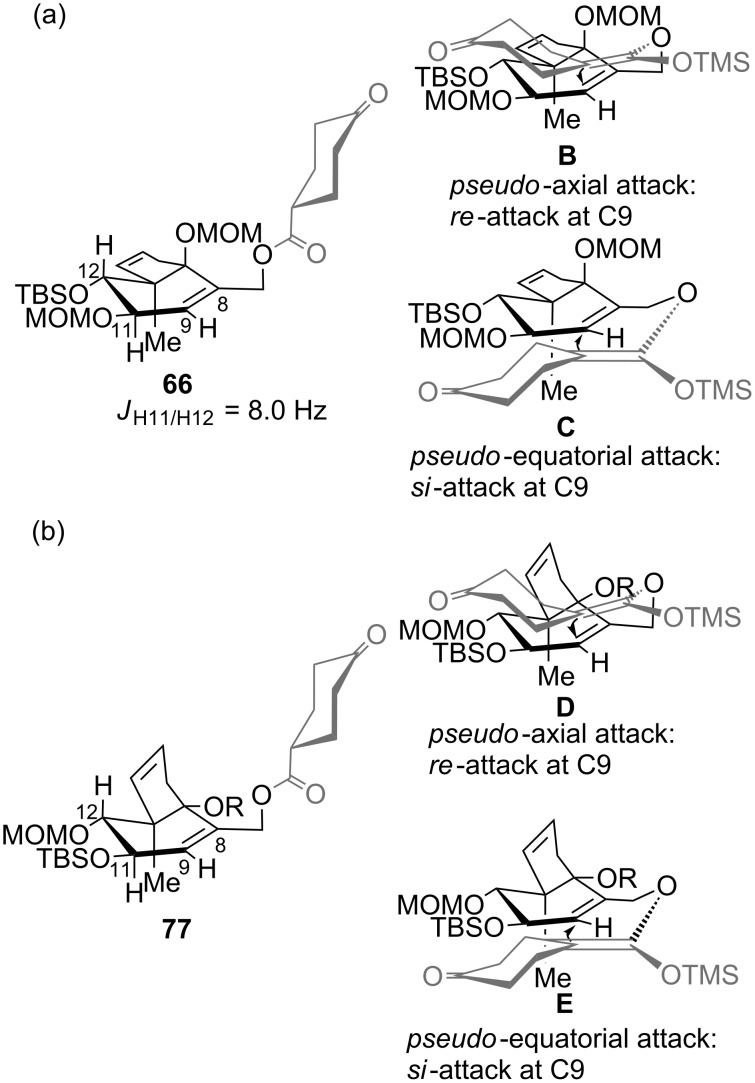
Conformations of rearrangement precursors **66** and **77** and possible transition states involved in the Ireland–Claisen rearrangements. R = MOM.

In contrast to the high face-selectivity in the rearrangement of the model systems, the reaction seems to take place from both sides of the bicyclic C–D system. However, in this case the *si*-face approach appears to be favoured (transition state **C**, Figure 3a). This might be explained by the rigidity of the bicyclic system, such that transition states **B** and **C** can compete without preference for a conformation that clearly favours axial attack. Underscored by MM2 conformational calculations, it is furthermore plausible that the C11 oxygen can form an H-bond to the terminal methyl C–H of the C14 MOM group, thus blocking a pseudo-axial approach and leading to the observed reversal in selectivity.

**Scheme 9:**
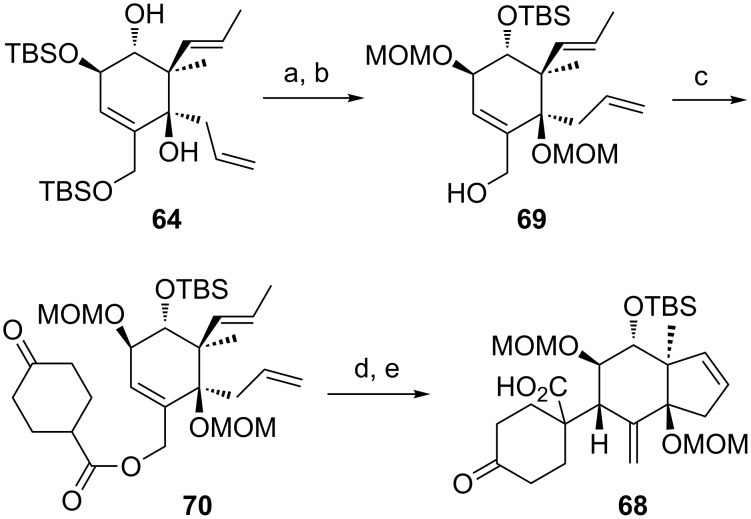
Synthesis and Ireland–Claisen rearrangement of allyl ester **70**. Reagents and conditions: a) DIPEA, MOMCl, NaI, THF, 65 °C, 3 days, 35%; b) HF·pyridine, THF, 20 h, rt, 83%; c) EDC·HCl, DMAP, CH_2_Cl_2_, 4-oxocyclohexanecarboxylic acid (**25**), rt, 24 h, 83%; d) LiHMDS, TMSCl/NEt_3_, toluene, 1 h at −78 °C, then gradual warming to 65 °C within 6 h and 40 h at 65 °C, yield n.d. dr = 1:0; e) Grubbs I, CH_2_Cl_2_, rt, 20 h, yield n.d.

We hypothesized that the diastereoselectivity could be improved by performing the rearrangement with the open-chain precursor **70** (Scheme 9), which should be less rigid than the bicyclic system **66** and therefore, in analogy to the results of the rearrangements with the model substrates, probably preferably rearrange via a pseudo*-*axial attack. The open-chain precursor **70** was obtained by MOM protection of diol **64**, selective cleavage of the primary TBS group and subsequent esterification with 4-oxocyclohexanecarboxylic acid (**25**).

However, in this case the [3,3]-sigmatropic rearrangement proceeded exclusively via pseudo-equatorial attack, giving after RCM the C9-*epi* limonoid scaffold **68** as single diastereomer. The sterically demanding rearrangement precursor **70** seems to allow only the undesired pseudo-equatorial attack.

**Scheme 10:**
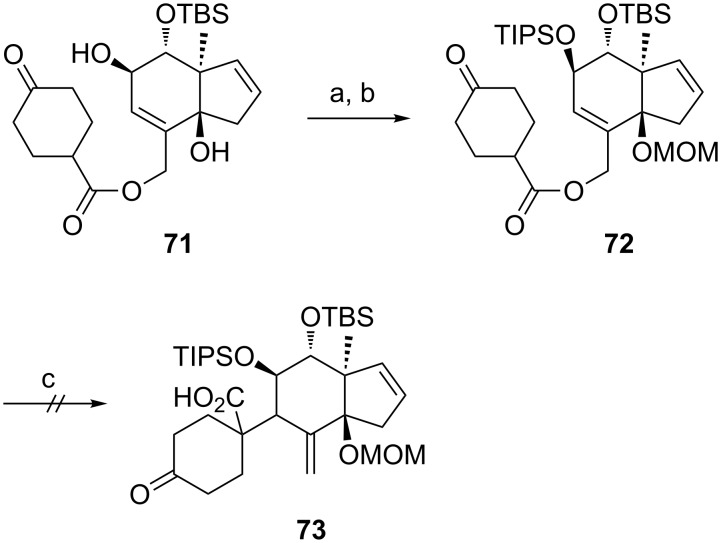
Synthesis and Ireland–Claisen rearrangement of allyl ester **72**. Reagents and conditions: a) TIPSOTf, pyridine, DMAP, rt, 22 h, 60%; b) DIPEA, MOMCl, THF, 25 h, 50 °C, 29%; c) LiHMDS, TMSCl/NEt_3_, toluene, 1 h at −78 °C, then gradual warming to 65 °C within 6 h and stirred for 2 days at 65 °C.

We envisaged that protecting the free secondary hydroxy group in **71** with a sterically demanding TIPS group might induce a conformational change of the rearrangement precursor and intended to investigate the influence of this conformational change on the diastereoselectivity of the Ireland–Claisen rearrangement (Scheme 10). However, the allyl ester **72** seems to be too sterically hindered to allow the [3,3]-sigmatropic rearrangement to proceed. After 2 days reaction time, only traces of rearrangement product **73** could be observed.

**Scheme 11:**
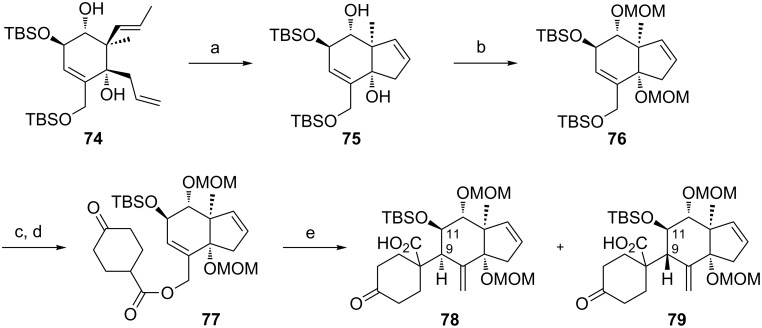
Synthesis of the C14-*epi* and C14/C9-*epi* B-*seco* limonoid scaffolds **78** and **79**. Reagents and conditions: a) Grubbs I, CH_2_Cl_2_, rt, 18 h, 86%; b) DIPEA, MOMCl, NaI, THF, 20 h, 65 °C, 89%; c) HF·pyridine, THF, 0 °C to rt, 24 h, 76%; d) EDC·HCl, DMAP, CH_2_Cl_2_, 4-oxocyclohexanecarboxylic acid (**25**), rt, 19 h, 92%; e) LiHMDS, TMSCl/NEt_3_, toluene, 1 h at −78 °C, then gradual warming to 65 °C within 6 h and stirred for 43 h at 65 °C, 78% yield, dr = 1.3:1 (**78**:**79**).

Moreover, in order to allow the synthetic access to further B-*seco* limonoid analogues, the C14-*epi* B*-seco* limonoid scaffold **78** and C14-*epi*/C9-*epi* scaffold **79** were accessed (Scheme 11). Starting from diol **74**, the C14-*epi* rearrangement precursor **77** was synthesized employing a sequence of ring-closing metathesis, TBS deprotection, esterification and MOM protection. The Ireland–Claisen rearrangement proceeded smoothly and gave a ca. 1.3:1 (**78**:**79**) mixture of diastereomers with the product resulting from the pseudo-axial attack of the silyl ketene acetal being the major diastereomer.

In analogy to the results above the transition states **D** and **E** seem to compete without any preference for a conformation that clearly favours axial attack (Figure 3b). Presumably because of the absence of electrostatic interactions in the pseudo-axial attack of the silyl ketene acetal (compare transition state **B** in Figure 3a: electrostatic interaction with -OMOM at C14), this approach is slightly favored.

**Scheme 12:**
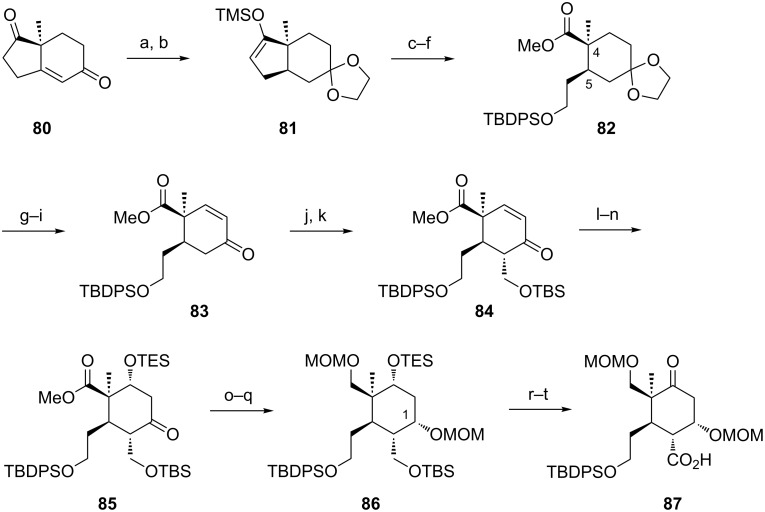
Synthesis of fully functionalized A ring **87**. Reagents and conditions: a) HO(CH_2_)_2_OH, THF, Pd/C, H_2_, pH 5, rt, overnight, 97%, de = 100%; b) LDA, TMSCl, THF, −78 °C to rt, 1.5 h, quant.; c) O_3_, CH_2_Cl_2_, −78 °C, then DMS, −78 °C to rt; d) TMSCHN_2_, CH_2_Cl_2_/MeOH (1:1), rt, 0.5 h, 59% (2 steps); e) NaBH_4_, MeOH, 0 °C, 0.5 h; f) TBDPSCl, imidazole, DMAP, CH_2_Cl_2_, 0.5 h, rt, 78% (2 steps); g) CH_2_Cl_2_/H_2_O/HClO_4_ (25:5:1), rt, 6 h; h) LiHMDS, TMSCl, THF, −78 °C to rt, 1.5 h; i) Pd(OAc)_2_, DMSO, O_2_, overnight, 72% (3 steps); j) LDA, 1*H*-benzotriazole-1-methanol, THF, −78 °C, 3 h, 67%, de = 100%; k) TBSCl, imidazole, DMF, rt, overnight, 90%; l) H_2_O_2_, NaOH, MeOH, 0 °C, 1 h, 80%, de = 100%; m) NaBH_4_, (PhSe)_2_, EtOH, 0 °C to rt, 10 min, 93%; n) TESCl, imidazole, DMF, 40 °C, 2 h, 99%; o) NaBH_4_, MeOH, 0 °C, 3 h, 64%; p) LiBH_4_, THF, 65 °C, 4 days, 67% (92% brsm); q) MOMCl, DIPEA, NaI, THF, 50 °C, 4 days, 96%, dr = 2:1; r) 5% TFA in CH_2_Cl_2_, rt, 0.5 h, 74%; s) DMP, NaHCO_3_, CH_2_Cl_2_, 0 °C, 3 h, 76%; t) NaClO_2_, NaH_2_PO_4_, 2-methyl-2-butene, *t-*BuOH/H_2_O (4:1), rt, 3.5 h, 91%.

**Synthesis of fully functionalized A ring.** Having these extensive studies completed we then focused on the synthesis of a suitable fully functionalized A ring **87** (Scheme 12) that after connection to the bicyclic C–D system and subsequent Ireland–Claisen rearrangement was supposed to give access to the entire framework of B-*seco* limonoids.

Explorations to access this fragment started from Hajos–Parrish ketone **80** [52] (Scheme 12), as we were inspired by a reaction sequence Arseniyadis et al. [53] used in their synthesis of a left-half taxoid building block. Regioselective protection of the less hindered ketone in **80** and diastereoselective hydrogenation of the double bond could be achieved in a known one-pot procedure [54–55] affording the *cis*-hydrindanone and providing the desired stereochemistry at C4 and C5. Ozonolytic cleavage of the corresponding silyl enol ether **81** followed by esterification with TMSCHN_2_ furnished the ester aldehyde that was reduced to the primary alcohol and protected to give TBDPS ether **82**. After selective cleavage of the acetal group by treatment with perchloric acid, installation of the double bond via Saegusa oxidation [56–57] of the previously formed TMS ether furnished enone **83** as the major regioisomer. Minor amounts of the undesired regioisomer could be separated by column chromatography. Hydroxymethylation of **83** using 1*H*-benzotriazole-1-methanol proceeded diastereoselectively due to substrate control [58]. The configuration was determined by the high coupling constant (*J*_H4/H5_ = 12.6 Hz) indicating the *trans*-diaxial orientation of H4 and H5. Silylation of the primary hydroxy group afforded compound **84** that was converted into the epoxide with complete stereocontrol [59]. The epoxide underwent regioselective opening [60–61] leading to the β-hydroxyketone that was temporarily masked as a TES ether.

Reduction of ketone **85** with NaBH_4_ resulted in the formation of two diastereomeric alcohols in 2:1 ratio. To our delight flash chromatography permitted smooth separation of the two compounds. nOe studies revealed that the main product is 1*S*-configurated as in most B-*seco* limonoids suggesting that an equatorial attack of the hydride is slightly preferred over the axial attack. However bearing in mind the elimination issues with β-alkoxy esters during Ireland–Claisen rearrangements discussed above we decided to continue the synthesis with both diastereomers as this elimination is expected to proceed faster in *syn*-substituted β-alkoxy esters since the hydrogen and the leaving group are in an antiperiplanar arrangement.

Continuing the synthesis with the 1*S*-isomer (Scheme 12), reduction of the ester moiety could be initiated by LiBH_4_ but proceeded sluggishly. For the protection of the corresponding diol we were limited to small protecting groups as introduction of the MOM-groups already occurred slowly and required high excess of reagents. Selective deprotection of the TBS and the TES ethers in **86** was achieved under acidic conditions. Oxidation of the resulting diol with Dess–Martin periodinane and subsequent Pinnick oxidation completed the synthesis.

**Scheme 13:**
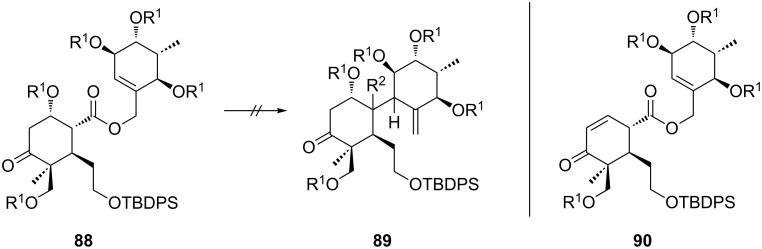
and Attempted Ireland–Claisen rearrangement of allyl ester **88**. R^1^ = MOM, R^2^ = CO_2_H.

**Ireland–Claisen rearrangements with fully decorated A ring.** With the suitable substituted fragment **87** in hand, we decided to initially perform the intended rearrangement with one of the model C rings. Thus carboxylic acid **87** was esterified with allylic alcohol **40** to give allyl ester **88** (Scheme 13).

Unfortunately exposure of **88** to the optimized conditions developed for the synthesis of the B-*seco* limonoid scaffold did not initiate the desired Ireland–Claisen rearrangement. Addition of HMPA was also fruitless. Noteworthy elimination of the MOM-protected hydroxy group at C1 occurred only to a minor extent under these conditions, but was not observed at all when HMPA was added. In situ formation of the TMS enol ether of **88** was detected, however as a consequence of the acidic work-up β-elimination of the OMOM group was induced resulting in α,β-unsaturated ketone **90**.

**Scheme 14:**
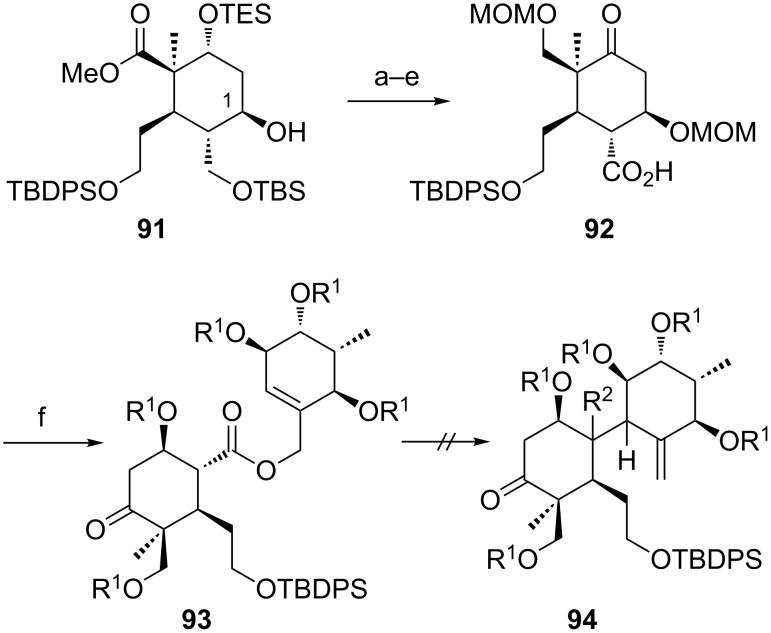
Synthesis and attempted Ireland–Claisen rearrangement of allyl ester **93**. Reagents and conditions: a) LiBH_4_, THF, 65 °C, overnight, 84%; b) MOMCl, DIPEA, NaI, THF, 50 °C, overnight, 89%; c) 8% TFA in CH_2_Cl_2_, rt, 0.5 h, 52%; d) DMP, NaHCO_3_, CH_2_Cl_2_, 0 °C, 3 h, 86%; e) NaClO_2_, NaH_2_PO_4_, 2-methyl-2-butene, *t-*BuOH/H_2_O (4:1), rt, 2.5 h, 99%; f) EDC·HCl, **40**, DMAP, CH_2_Cl_2_, rt, 2 d, 47%. R^1^ = MOM, R^2^ = CO_2_H.

Despite these unsatisfactory results we continued with the synthesis of C1-*epi* allyl ester **93** starting from alcohol **91** employing the same reaction sequence of ester reduction, diol protection, desilylation, oxidation and esterification (Scheme 14). Attempts to rearrange **93** under the optimized conditions were again unsuccessful. As expected, elimination of the OMOM group during the reaction was not detected due to the unfavoured orientation of the hydrogen and the leaving group. Subsequent acidic treatment did again lead to formation of enone **90**.

We assume that the additional substituents as well as the intermediary formed TMS ether cause excessive steric bulk and rigidity that prevent the silyl ketene acetals from adopting the required conformation. Hence, we considered enone **90** as an alternative rearrangement precursor as many B-*seco* limonoids exhibit a double bond in this position (see 21-hydroxytoonacilide (**1**)). However, despite the incapacity to form the silyl enol ether as well as the lack of the C1-substituent allyl ester **90** failed to furnish the corresponding carboxylic acid using the general conditions.

**Scheme 15:**
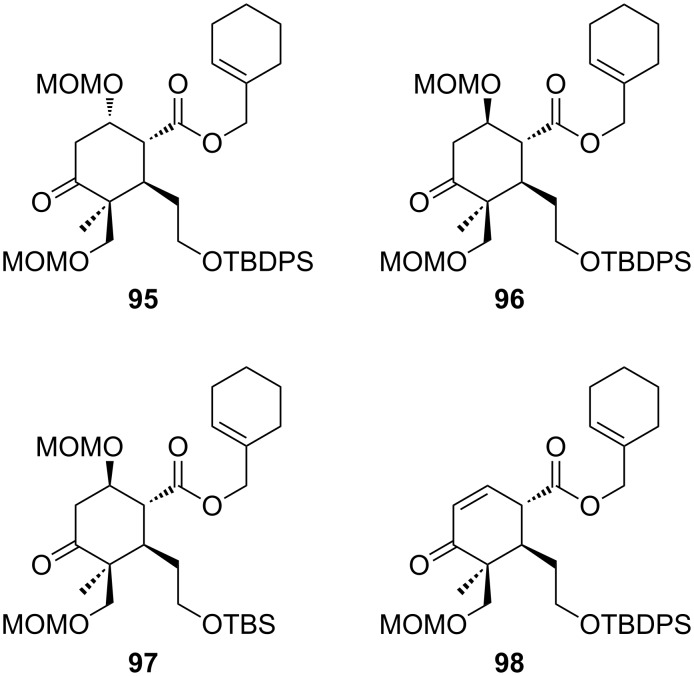
Allyl esters tested in the Ireland–Claisen rearrangement.

In order to explore whether the whole system or whether the A ring **87** itself is too crowded to allow the rearrangement to proceed, we used a completely undecorated C ring. For this purpose rearrangement precursors **95**, **96**, and **97** were synthesized (see Supporting Information File 1) and investigated with respect to their behavior in the [3,3]-sigmatropic rearrangement (Scheme 15). Unfortunately all attempts to procure this transformation using different bases (LiHMDS, KHMDS, LDA), additives (Et_3_N, HMPA), solvents (THF, toluene) and temperatures (up to 110 °C) completely failed. Indeed the results are in accordance with those obtained earlier. All allyl esters were converted to the corresponding TMS ethers under the rearrangement conditions. Formation of the β-elimination product was exclusively observed for the 1*S*-configurated allyl ester **95** in the absence of HMPA. Replacing the sterically demanding TBDPS group by the smaller TBS group in **97** could not initiate the rearrangement either. Furthermore, α,β-unsaturated ketone **98**, that was generated during the acidic work-up, did not show any conversion (Scheme 15).

## Conclusion

In view of the results obtained we conclude that an Ireland–Claisen rearrangement is not a suitable method to build up the completely decorated scaffold of the B-*seco* limonoid natural products, as it failed when a fully substituted A ring is used. Presumably steric constraints in combination with the rigidity caused by the intermediary formed silyl enol ether are responsible for the failure as it has been indicated before for substrates that contain a rigid BDA group or a bulky TIPS group.

## Supporting Information

File 1 Experimental procedures and characterisation data.
