# Supplementary material for: Synthesis of the B-seco limonoid core scaffold
Source: Beilstein J Org Chem. 2014 Jan 16;10:194–208. doi: 10.3762/bjoc.10.15 (PMC3943702; doi:10.3762/bjoc.10.15)
Supplement: File 1 — Experimental procedures and characterisation data. [file Beilstein_J_Org_Chem-10-194-s001.pdf]

# Supporting Information

# for

# Synthesis of the B-*seco* limonoid core scaffold

Hanna Bruss[1,2], Hannah Schuster[1,2], Rémi Martinez[1], Markus Kaiser[3], Andrey P. Antonchick[1,2] and Herbert Waldmann*[1,2]

Address: [1]Abteilung Chemische Biologie, Max-Planck-Institut für Molekulare Physiologie, Otto-Hahn-Straße 11, 44227 Dortmund, Germany, [2]Fakultät für Chemie und Chemische Biologie, Technische Universität Dortmund, Otto-Hahn-Straße 6, 44227 Dortmund, Germany and [3]Chemical Biology, Zentrum für Medizinische Biotechnologie, Fakultät für Biologie, Universität Duisburg-Essen, Universitätsstraße 2, 45117 Essen, Germany

Email: Herbert Waldmann - herbert.waldmann@mpi-dortmund.mpg.de

* Corresponding author

## Experimental procedures and characterisation data

**General Information.** Unless otherwise noted, all commercially available chemicals were used as provided without further purification. Dry solvents were in general used as received, dichloromethane was distilled from calcium hydride under an atmosphere of argon. All reactions were carried out with dry solvents under argon atmosphere using oven-dried glassware. Analytical thin-layer chromatography (TLC) was performed on Merck silica gel aluminium plates with F-254 indicator, visualised using combinations of irradiation with UV light and staining with potassium permanganate. Flash chromatography was performed using silica gel for chromatography 0.040–0.063 mm, 60A purchased from Acros Organic. Solvent mixtures are understood as volume/volume. $^{1}$H NMR and $^{13}$C NMR were recorded on a Bruker DRX400 (400 MHz), DRX500 (500 MHz), DRX600 (600 MHz) or a Varian Mercury - 400 Oxford NMR spectrometer in $CDCl_3$, $C_6D_6$ or $CD_3OD$. Data are reported in the following order: chemical shift (δ) in ppm; multiplicities are indicated bs (broadened singlet), s (singlet), d (doublet), t (triplet), q (quartet), m (multiplet); coupling constants (J) are given in Hertz (Hz). High resolution mass spectra were recorded on a LTQ Orbitrap mass spectrometer coupled to an Accela HPLC-System (HPLC column: Hypersyl GOLD, 50 mm × 1 mm, 1.9 μm). Optical rotations were measured in a Schmidt + Haensch Polartronic HH8 polarimeter. Melting points were recorded on a Büchi Melting point B-540. Experimental procedures and analytical data for compounds **15**, **17**, **29**, **30**, **33**, **34**, **35**, **36**, **37**, **38**, **39**, **40**, **41**, **42**, **51**, **54**, **55**, **61**, **62**, **63**, **64**, **65**, **66**, **67**, **68**, **74** and **75** have already been described [1].

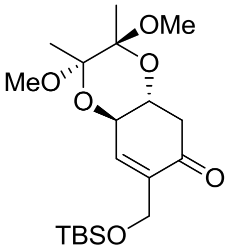

**S1**

**Silylether S1.** To a stirred solution of alcohol **17** (500 mg, 1.84 mmol) in $CH_2Cl_2$ (18 ml) at 0 °C were added 2,6-lutidine (642 µl, 5.51 mmol) and TBSOTf (464 µl, 2.02 mmol). The resulting solution was stirred for 15 min at 0 °C. After addition of aqueous saturated $NaHCO_3$-solution (20 ml), the two layers were separated and the aqueous layer was extracted with $Et_2O$ (3 × 20 ml). The combined organic layers were dried ($MgSO_4$) and evaporated under reduced pressure. Purification by flash chromatography on silica with petroleum ether/EtOAc 10:1 as eluent gave silylether **S1** (710 mg, quant.) as a colourless oil.

$\mathbf{R}_f$ = 0.28 (petroleum ether:EtOAc = 10:1); $[\boldsymbol{\alpha}]_{\mathbf{D}}^{\mathbf{20}}$ = +40.2 (*c* = 1.20 in $CH_2Cl_2$); **$^1$H NMR** (400 MHz, $CDCl_3$) δ = 6.87 – 6.83 (m, 1H, 9-H), 4.52 – 4.47 (m, 1H, 11-H), 4.34 (ddd, *J* = 15.8, 3.6, 2.2 Hz, 1H, 1´-$H_a$), 4.24 (ddd, *J* = 15.8, 3.1, 2.1 Hz, 1H, 1´-$H_b$), 3.99 (ddd, *J* = 13.6, 9.1, 4.9 Hz, 1H, 12-H), 3.31 (s, 3H, -OC$H_3$), 3.24 (s, 3H, -OC$H_3$), 2.70 (dd, *J* = 16.3, 4.9 Hz, 1H, 13-$H_a$), 2.48 (dd, *J* = 16.3, 13.6 Hz, 1H, 13-$H_b$), 1.35 (s, 3H, -C$H_3$), 1.31 (s, 3H, -C$H_3$), 0.90 (s, 9H, -SiC(C$H_3$)$_3$), 0.05 (s, 6H, Si(C$H_3$)$_2$) ppm; **$^{13}$C NMR** (101 MHz, $CDCl_3$) δ = 196.26 (q, 14-C), 142.41 (t, 9-C), 139.88 (q, 8-C), 100.96 (q, BDA), 99.91 (q, BDA), 69.69 (t, 12-C), 68.42 (t, 11-C), 59.85 (s, 1´-H), 48.42 (p, -O*C*$H_3$), 48.29 (p, -O*C*$H_3$), 42.57 (q, -*C*($CH_3$)$_3$), 26.17 (p, 3C, -SiC(*C*$H_3$)$_3$), 18.57 (q, -Si*C*($CH_3$)$_3$), 17.99 (p, -*C*$H_3$), 17.91 (p, -*C*$H_3$), -5.22 (p, -Si(C$H_3$)$_2$), -5.31 (p, -Si(C$H_3$)$_2$) ppm; **HRMS-ESI** ($C_{19}H_{34}O_6Si$): calc. for $[M+H]^+$: 387.21974, found: 387.22034.

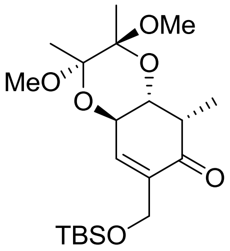


**S2**

**Silylether S2.** To a stirred solution of enone **S1** (750 mg, 1.94 mmol) in THF (30 ml) and DMPU (6 ml) at –78 °C was added LiHMDS (1 M in THF, 4.85 ml, 4.85 mmol). The reaction mixture was stirred for 30 min at –78 °C. After the addition of MeI (1.21 ml, 19.4 mmol), the reaction mixture was allowed to warm to –10 °C and stirred for 2 h at –10 °C. Aqueous saturated $NH_4Cl$-solution (30 ml) was added and the resulting two layers were separated. The aqueous layer was extracted with $Et_2O$ (3 × 30 ml) .The combined organic layers were dried ($MgSO_4$) and evaporated under reduced pressure. Purification by flash chromatography on silica with petroleum ether/EtOAc 10:1 as eluent gave **S2** (709 mg, 91%) as a colourless oil.

$\mathbf{R}_f$ = 0.47 (petroleum ether:EtOAc = 10:1); $[\alpha]_D^{20}$ = +20.7 (*c* = 1.10 in $CH_2Cl_2$); $^1$**H NMR** (400 MHz, $CDCl_3$) δ = 6.82 – 6.80 (m, 1H, 9-H), 4.64 – 4.58 (m, 1H, 11-H), 4.33 (ddd, *J* = 15.8, 3.4, 2.1 Hz, 1H, 1´-$H_a$), 4.29 – 4.22 (m, 1H, 1´-$H_b$), 4.00 (dd, *J* = 9.4, 5.6 Hz, 1H, 12-H), 3.30 (s, 3H, -OC$H_3$), 3.21 (s, 3H, -OC$H_3$), 2.69 (qd, *J* = 7.5, 5.6 Hz, 1H, 13-H), 1.34 (s, 3H, -C$H_3$), 1.30 (s, 3H, -C$H_3$), 1.16 (d, *J* = 7.5 Hz, 3H, 2´-H), 0.90 (s, 9H, -SiC(C$H_3$)$_3$), 0.06 (s, 6H, -Si(C$H_3$)$_2$) ppm; $^{13}$**C NMR** (101 MHz, $CDCl_3$) δ = 200.39 (q, 14-C), 141.53 (t, 9-C), 138.34 (q, 8-C), 100.90 (q, BDA), 100.00 (q, BDA), 70.09 (t, 12-C), 65.12 (t, 11-C), 60.07 (s, 1´-C), 48.33 (p, -O*C*H$_3$), 48.13 (p, -O*C*H$_3$), 45.13 (q, -*C*(CH$_3$)$_3$), 26.18 (p, 3C, -SiC(*C*H$_3$)$_3$), 18.58 (q, -Si*C*(CH$_3$)$_3$), 18.05 (p, -*C*H$_3$), 17.86 (p, -*C*H$_3$), 10.25 (p, 2´-C), -5.20 (p, -Si(C$H_3$)$_2$), -5.30 (p, -Si(C$H_3$)$_2$) ppm; **HRMS-ESI** ($C_{20}H_{36}O_6Si$): calc. for $[M+H]^+$:401.23539, found: 401.23530.

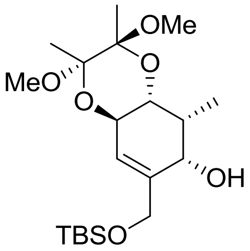

**18**

**Alcohol 18.** To a stirred solution of ketone **S2** (392 mg, 979 μmol) in methanol (100 ml) at 0 °C were added $CeCl_3 \cdot 7H_2O$ (401 mg, 1.08 mmol) and $NaBH_4$ (37 mg, 0.98 mmol). The reaction mixture was stirred for 15 min at 0 °C. After the addition of aqueous saturated $NH_4Cl$-solution (50 ml), the solution was concentrated under reduced pressure. The residue was solved in EtOAc (100 ml) and water (100 ml) and the two layers were separated. The aqueous layer was extracted with EtOAc (3 × 100 ml) and the combined organic layers were washed with brine (200 ml), dried ($MgSO_4$) and evaporated under reduced pressure. Purification by flash chromatography on silica with petroleum ether/ EtOAc 5:1 to 3:1 as eluent gave alcohol **18** (355 mg, 90%) as a colourless oil.

$\mathbf{R}_f$ = 0.50 (petroleum ether:EtOAc = 5:1); $[\alpha]_D^{20}$ = +61.8 (*c* = 1.10 in $CH_2Cl_2$); **$^1$H NMR** (500 MHz, $CDCl_3$) δ = 5.45 – 5.42 (m, 1H, 9-H), 4.56 (bs, 1H, 14-H), 4.32 – 4.27 (m, 1H, 11-H), 4.22 (d, *J* = 12.7 Hz, 1H, 1´-$H_a$), 4.15 (d, *J* = 12.7 Hz, 1H, 1´-$H_b$), 3.64 (dd, *J* = 9.2, 3.6 Hz, 1H, 12-H), 3.18 (s, 3H, -OC$H_3$), 3.15 (s, 3H, -OC$H_3$), 2.38 – 2.31 (m, 1H, 13-H), 1.23 (s, 6H, 2 x –C$H_3$), 0.94 (d, *J* = 7.1 Hz, 3H, 2´-H), 0.83 (s, 9H, -SiC(C$H_3$)$_3$), 0.02 (s, 3H, -Si(C$H_3$)$_2$), 0.02 (s, 3H, -Si(C$H_3$)$_2$) ppm; **$^{13}$C NMR** (126 MHz, $CDCl_3$) δ = 139.76 (q, 8-C), 122.07 (t, 9-C), 100.40 (q, BDA), 100.25 (q, BDA), 71.92 (t, 14-C), 70.00 (t, 12-C), 65.61 (t, 11-C), 65.51 (s, 1´-C), 47.87 (p, -O*C*$H_3$), 47.79 (p, -O*C*$H_3$), 37.64 (t, 13-C) , 25.93 (p, 3C, -SiC(*C*$H_3$)$_3$), 18.27 (q, -Si*C*(C$H_3$)$_3$), 18.03 (p, -*C*$H_3$), 17.89 (p, -*C*$H_3$), 7.33 (p, 2´-C), -5.30 (p, -Si(*C*$H_3$)$_2$), -5.37 (p, -Si(*C*$H_3$)$_2$) ppm.

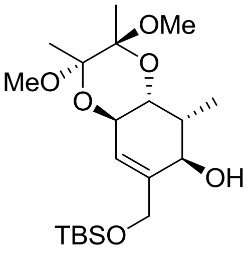

**S3**

**Alcohol S3.** Alcohol **18** (942 mg, 2.34 mmol) was solved in toluene (50 ml). To the solution were added $Ph_3P$ (4.60 g, 17.6 mmol), *p*-nitrobenzoic acid (2.93 g, 17.6 mmol) and DEAD (2.76 ml, 17.6 mmol). The reaction mixture was stirred for 18 h at room temperature and then concentrated under reduced pressure. Filtration through a plug of silica (petroleum ether/EtOAc 15:1) gave the crude product, which was used in the next step without further purification.

The crude product was solved in a mixture of methanol (40 ml), $Et_2O$ (13 ml) and saturated aqueous $K_2CO_3$-solution (27 ml) and the resulting solution was stirred for 1 h at room temperature. The two layers were separated and the organic layer was washed with saturated aqueous $NH_4Cl$-solution (30 ml). The aqueous layer was extracted with $Et_2O$ (3 × 50 ml) and the combined organic extracts were dried ($MgSO_4$) and evaporated under reduced pressure. Purification by flash chromatography on silica with petroleum ether/EtOAc 4:1 as eluent gave **S3** (607 mg, 64% over 2 steps) as a colourless oil.

$\mathbf{R_f}$ = 0.56 (petroleum ether:EtOAc = 3:1); $[\alpha]_D^{20}$ = +100.6 (*c* = 1.00 in $CH_2Cl_2$); **$^1$H NMR** (500 MHz, $CDCl_3$) δ = 5.62 – 5.60 (m, 1H, 9-H), 4.26 – 4.20 (m, 2H, 1´-$H_a$, 11-H), 4.14 (d, *J* = 11.5 Hz, 1H, 1´-$H_b$), 4.07 (dd, *J* = 9.4, 4.2 Hz, 1H, 12-H), 3.99 (bs, 1H, 14-H), 3.24 (s, 3H, -OC$H_3$), 3.23 (s, 3H, -OC$H_3$), 3.15 (bs, 1H, OH), 2.18 – 2.09 (m, 1H, 13-H), 1.31 (s, 3H, -C$H_3$), 1.28 (s, 3H, -C$H_3$), 0.93 (d, *J* = 7.4 Hz, 3H, 2´-H), 0.88 (s, 9H, -SiC(C$H_3$)$_3$), 0.08 (s, 3H, -Si(C$H_3$)$_2$), 0.06 (s, 3H, -Si(C$H_3$)$_2$) ppm; **$^{13}$C NMR** (126 MHz, $CDCl_3$) δ = 137.75 (q, 8-C), 125.55 (t, 9-C), 100.68 (q, BDA), 100.46 (q, BDA), 73.81 (t, 14-C), 67.65 (t, 12-C), 67.55 (t, 11-C), 65.54 (s, 1´-C), 48.03 (p, -O*C*$H_3$), 48.00 (p, -O*C*$H_3$), 38.46 (t, 13-C), 26.06 (p, 3C, -SiC(*C*$H_3$)$_3$), 18.38 (q, -Si*C*($CH_3$)$_3$), 18.17 (p, -*C*$H_3$), 18.09 (p, -*C*$H_3$), 10.92 (p, 2´-C), -5.34 (p, 3C, -Si(C*H*$_3$)$_2$) ppm; **HRMS-ESI** ($C_{20}H_{38}O_6Si$): calc. for $[M+Na]^+$: 425.23299, found: 425.23259.

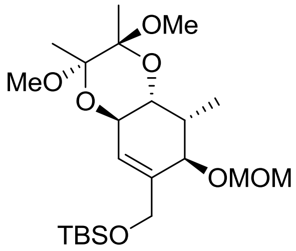


**S4**

**MOM-Ether S4.** To a stirred solution of alcohol **S3** (1.70 g, 4.22 mmol) in $CH_2Cl_2$ (100 ml) were added DIPEA (5.88 ml, 33.8 mmol) and MOMCl (2.56 ml, 33.8 mmol). The reaction mixture was stirred for 16 h under reflux. After the addition of water (50 ml), the two layers were separated and the aqueous layer was extracted with $CH_2Cl_2$ (3 × 50 ml). The combined organic layers were dried ($MgSO_4$) and evaporated under reduced pressure. Purification by flash chromatography on silica with petroleum ether/EtOAc 6:1 as eluent gave **S4** (1.88 g, 99%) as a colourless oil.

$\mathbf{R}_f$ = 0.50 (petroleum ether:EtOAc = 5:1); $[\boldsymbol{\alpha}]_{\mathbf{D}}^{\mathbf{20}}$ = +81.7 (*c* = 0.98 in $CH_2Cl_2$); **$^1$H NMR** (400 MHz, $CDCl_3$) δ = 5.69 – 5.67 (m, 1H, 9-H), 4.69 (d, *J* = 7.0 Hz, 1H, -OC$H_2$OC$H_3$), 4.63 (d, *J* = 7.0 Hz, 1H, -OC$H_2$OC$H_3$), 4.24 – 4.19 (m, 1H, 11-H), 4.17 – 4.12 (m, 1H, 1´-$H_a$), 4.12 – 4.07 (m, 1H, 1´-$H_b$), 3.97 (dd, *J* = 9.4, 4.2 Hz, 1H, 12-H), 3.80 (bs, 1H, 14-H), 3.36 (s, 3H, -O$CH_2$OC$H_3$), 3.24 (s, 3H, -OC$H_3$), 3.21 (s, 3H, -OC$H_3$), 2.28 – 2.19 (m, 1H, 13-H), 1.29 (s, 3H, -C$H_3$), 1.28 (s, 3H, -C$H_3$), 0.94 (d, *J* = 7.4 Hz, 3H, 2´-H), 0.88 (s, 9H, -SiC(C$H_3$)$_3$) 0.04 (s, 3H, -Si(C$H_3$)$_2$), 0.04 (s, 3H, -Si(C$H_3$)$_2$) ppm; **$^{13}$C NMR** (101 MHz, $CDCl_3$) δ = 137.90 (q, 8-C), 123.96 (t, 9-C), 100.41 (q, BDA), 100.58 (q, BDA), 96.88 (s, -*C*$H_2$-OMe), 77.91, 68.09, 65.17, 64.12 (11-C, 12-C, 14-C, 1´-C), 55.90 (p, -O*C*$H_3$), 47.99 (p, -O*C*$H_3$), 47.80 (p, -O*C*$H_3$), 36.01 (t, 13-C), 26.15 (p, 3C, Si-C(*C*$H_3$)$_3$), 18.55 (p, -*C*$H_3$), 18.15 (p, -*C*$H_3$),18.12 (q, Si-*C*($CH_3$)$_3$), 10.96 (p, 2´-C), -4.99 (p, Si(*C*$H_3$)$_2$), -5.23 (p, Si(*C*$H_3$)$_2$) ppm; **HRMS-ESI** ($C_{22}H_{42}O_7Si$): calc. for $[M+H]^+$: 447.27726, found: 447.27710.

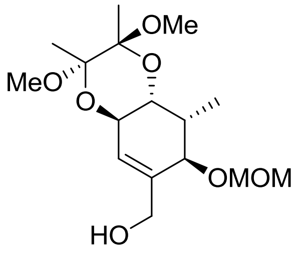

**19**

**Alcohol 19.** To a stirred solution of silyl ether **S4** (1.88 g, 4.20 mmol) in THF (100 ml) was added TBAF (1 M in THF, 6.3 ml, 6.3 mmol). The reaction mixture was stirred for 20 min at room temperature and then concentrated under reduced pressure. Purification by flash chromatography on silica (petroleum ether/EtOAc 4:3 to 2:1) gave alcohol **19** (1.32 g, 94%) as a white solid.

$\mathbf{R}_f$ = 0.35 (petroleum ether:EtOAc = 1:1); $[\alpha]_{\mathrm{D}}^{20}$ = +76.7 (*c* = 0.94 in $CH_2Cl_2$); **$^1$H NMR** (400 MHz, $CDCl_3$) δ = 5.72 (bs, 1H, 9-H), 4.72 (d, *J* = 6.8 Hz, 1H, -OC$H_2$OC$H_3$), 4.64 (d, *J* = 6.8 Hz, 1H, -OC$H_2$OCH$_3$), 4.24 – 4.07 (m, 3H, 11-H, 1´-H$_a$, 1´-H$_b$), 3.94 (dd, *J* = 9.4, 4.3 Hz, 1H, 12-H), 3.89 (bs, 1H, 14-H), 3.39 (s, 3H, -OCH$_2$OC$H_3$), 3.24 (s, 3H, -OC$H_3$), 3.21 (s, 3H, -OC$H_3$), 2.28 – 2.19 (m, 1H, 13-H), 1.94 (bs, 1H, OH), 1.29 (s, 3H, -C$H_3$), 1.28 (s, 3H, -C$H_3$), 0.96 (d, *J* = 7.4 Hz, 3H, 2´-H) ppm; **$^{13}$C NMR** (101 MHz, $CDCl_3$) δ = 137.84 (q, 8-C), 125.74 (t, 9-C), 100.57 (q, BDA), 100.40 (q, BDA), 96.83 (s, -*C*H$_2$-OMe), 78.38, 67.86, 64.89, 64.46 (11-C, 12-C, 14-C, 1´-C), 56.09 (p, -O*C*H$_3$), 47.97 (p, -O*C*H$_3$), 47.82 (p, -O*C*H$_3$), 35.89 (t, 13-C), 18.08 (p, -*C*H$_3$), 18.05 (p, -*C*H$_3$), 11.13 (p, 2´-C) ppm; **HRMS-ESI** ($C_{16}H_{28}O_7$): calc. for $[M+Na]^+$: 355.17272, found: 355.17279.

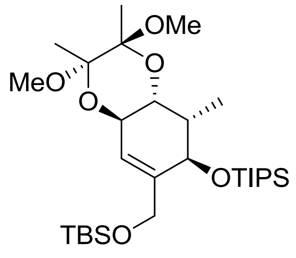

**S5**

***Bis*-Silylether S5.** To a stirred solution of alcohol **S3** (94 mg, 0.23 mmol) in DMF (3 ml) at 0 °C were added 2,6-lutidine (163 µl, 1.40 mmol) and TIPSOTf (0.19 ml, 0.70 mmol). The reaction mixture was stirred for 15 min at 0 °C and 3 h at room temperature. After the

addition of MeOH (5 ml) and aqueous saturated $NaHCO_3$-solution (5 ml), the resulting two layers were separated and the aqueous layer was extracted with $Et_2O$ (3 × 10 ml). The combined organic layers were washed with brine (20 ml), dried ($MgSO_4$) and evaporated under reduced pressure. Purification by flash chromatography on silica (petroleum ether/EtOAc 30:1) gave *bis*-silylether **S5** (129 mg, quant.) as a colourless oil.

**$R_f$** = 0.41 (petroleum ether:EtOAc = 20:1); $[\alpha]_D^{20}$ = +67.4 (*c* = 1.17 in $CH_2Cl_2$); **HRMS-ESI** ($C_{29}H_{58}O_6Si_2$): calc. for $[M+NH_4]^+$: 576.41102, found: 576.41068.

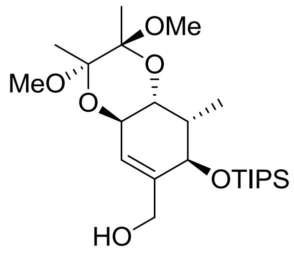


**20**

**Alcohol 20.** To a stirred solution of *bis*-silyl ether **S5** (125 mg, 224 μmol) in MeOH (8.0 ml) and THF (0.8 ml) was added *p*TSA (112 mg, 447 μmol). The solution was stirred for 30 h at room temperature. After the addition of $Et_2O$ (10 ml) and aqueous saturated $NaHCO_3$-solution (10 ml), the resulting two layers were separated and the aqueous layer was extracted with $Et_2O$ (3 × 10 ml). The combined organic layers were dried ($MgSO_4$) and evaporated under reduced pressure. Purification by flash chromatography on silica (petroleum ether/EtOAc 6:1) gave **20** (77 mg, 77%) as a white solid.

**$R_f$** = 0.18 (petroleum ether:EtOAc = 5:1); $[\alpha]_D^{20}$ = +89.3 (*c* = 1.05 in $CH_2Cl_2$); **$^1$H NMR** (500 MHz, $CDCl_3$) δ = 5.66 (s, 1H, 9-H), 4.23 (d, *J* = 9.4 Hz, 1H, 11-H), 4.15 (bs, 3H, 1´-$H_a$, 1´-$H_b$, 14-H), 4.08 (dd, *J* = 9.4, 4.0 Hz, 1H, 12-H), 3.23 (s, 3H, -OC*H*$_3$), 3.22 (s, 3H, -OC*H*$_3$), 2.17 – 2.07 (m, 1H, 13-H), 1.31 (s, 3H, -C*H*$_3$), 1.28 (s, 3H, -C*H*$_3$), 1.06 (s, 21H, -Si(C*H*(C*H*$_3$)$_2$)$_3$), 0.93 (d, *J* = 7.4 Hz, 3H, 2´-H) ppm; **$^{13}$C NMR** (126 MHz, $CDCl_3$) δ = 139.88 (q, 8-C), 124.20 (t, 9-C), 100.64 (q, BDA), 100.45 (q, BDA), 73.11 (t, 14-C), 67.66 (t, 12-C), 65.32 (t, 11-C), 64.62 (s, 1´-C), 48.12 (p, -O*C*H$_3$), 47.99 (p, -O*C*H$_3$), 39.47 (t, 13-C), 18.41 (p, -*C*H$_3$), 18.38 (p, -*C*H$_3$), 18.24 (t, -Si(*C*H(CH$_3$)$_2$)$_3$), 18.12 (t, -Si(*C*H(CH$_3$)$_2$)$_3$), 12.99 (p, 6C, -Si(CH(*C*H$_3$)$_2$)$_3$), 10.89 (p, 2´-C) ppm; (1 x -Si(*C*H(CH$_3$)$_2$)$_3$ is missing); **HRMS-ESI** ($C_{23}H_{44}O_6Si$): calc. for $[M+Na]^+$: 467.27994, found: 467.27957.

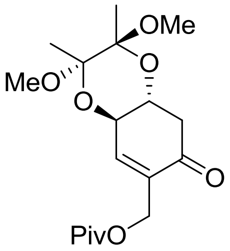

**S6**

**Pivalate S6.** To a stirred solution of alcohol **17** (1.50 g, 5.51 mmol) in pyridine (23 ml) at –15 °C were added DMAP (134 mg, 1.10 mmol) and PivCl (1.02 ml, 8.26 mmol). The reaction mixture was allowed to warm to room temperature and stirred for 2 h. The solution was evaporated under reduced pressure and the residue was dried under high vacuum. The crude product was solved in $CH_2Cl_2$ (50 ml) and then washed with aqueous saturated $NaHCO_3$-solution. The aqueous layer was extracted with $CH_2Cl_2$ (3 × 50 ml) and the combined organic layers were dried ($MgSO_4$) and evaporated under reduced pressure. Purification by flash chromatography on silica with petroleum ether/EtOAc (9:1) as eluent gave pivalate **S6** (1.51 g, 77%) as a colourless oil.

**R*f*** = 0.26 (petroleum ether:EtOAc = 7:1); $[\boldsymbol{\alpha}]_{\mathbf{D}}^{\mathbf{20}}$ = + 48.5 (*c* = 1.14 in $CH_2Cl_2$); **$^1$H NMR** (500 MHz, $CDCl_3$) δ = 6.73 – 6.70 (m, 1H, 9-H), 4.72 – 4.67 (m, 1H, 1´-$H_a$), 4.67 – 4.61 (m, 1H, 1´-$H_b$), 4.49 – 4.45 (m, 1H, 11-H), 3.97 (ddd, *J* = 13.7, 9.1, 4.8 Hz, 1H, 12-H), 3.27 (s, 3H, -OC$H_3$), 3.21 (s, 3H, -OC$H_3$), 2.71 (dd, *J* = 16.4, 4.8 Hz, 1H, 13-$H_a$), 2.46 (dd, *J* = 16.4, 13.7 Hz, 1H, 13-$H_b$), 1.31 (s, 3H, -C$H_3$), 1.27 (s, 3H, -C$H_3$), 1.17 ppm (s, 9H, -C(C$H_3$)$_3$); **$^{13}$C NMR** (126 MHz, $CDCl_3$) δ = 195.09 (q, 14-C), 177.97 (q, -O*C*(O)C($CH_3$)$_3$), 144.27 (t, 9-C), 135.74 (q, 8-C), 100.99 (q, BDA), 99.89 (q, BDA), 69.32 (t, 11-C), 68.07 (t, 12-C), 60.60 (s, 1´-C), 48.38 (p, -C(O*C*$H_3$)), 48.25 (p, -C(O*C*$H_3$)), 42.19 (s, 13-C), 38.99 (q, -*C*($CH_3$)$_3$), 27.36 (p, 3C, -C(*C*$H_3$)$_3$), 17.85 (p, -C(*C*$H_3$)), 17.79 (p, -C(*C*$H_3$)) ppm; **HRMS-ESI** ($C_{18}H_{28}O_7$): calc. for $[M+Na]^+$: 379.17272, found: 379.17260.

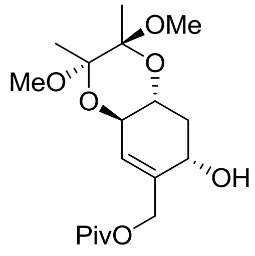


**21**

**Pivalate 21.** To a stirred solution of enone **S6** (850 mg, 2.38 mmol) in MeOH (25 ml) at 0 °C were added $CeCl_3{\cdot}7H_2O$ (977 mg, 2.62 mmol) and $NaBH_4$ (90 mg, 2.3 mmol). The reaction mixture was stirred for 30 min at room temperature and then quenched with aqueous saturated $NH_4Cl$-solution (30 ml). The solution was concentrated under reduced pressure and the residue was solved in EtOAc (20 ml) and $H_2O$ (20 ml). The two layers were separated and the aqueous layer was extracted with EtOAc (3 × 20 ml). The combined organic layers were washed with brine (20 ml), dried ($MgSO_4$) and evaporated under reduced pressure. Purification by flash chromatography on silica (petroleum ether/EtOAc 3:1) gave alcohol **21** (819 mg, 96%) as a colourless oil.

**$R_f$** = 0.38 (petroleum ether:EtOAc = 2:1); $[\alpha]_D^{20}$ = +50.5 (*c* = 1.33 in $CH_2Cl_2$); **$^1$H NMR** (400 MHz, $CDCl_3$) δ = 5.68 – 5.66 (m, 1H, 9-H), 4.94 – 4.88 (m, 1H, 1´-$H_a$), 4.36 (d, *J* = 12.8 Hz, 1H, 1´-$H_b$), 4.37 – 4.30 (m, 1H, 14-H), 4.30 – 4.24 (m, 1H, 11-H), 3.67 (ddd, *J* = 13.1, 8.8, 3.3 Hz, 1H, 12-H), 3.25 (s, 3H, -OC*H*3), 3.24 (s, 3H, -OC*H*3), 2.80 (bs, 1H, OH), 2.32 (ddd, *J* = 12.2, 6.5, 3.3 Hz, 1H, 13-$H_a$), 1.71 (ddd, *J* = 13.1, 12.2, 9.5 Hz, 1H, 13-$H_b$), 1.30 (s, 3H, -C*H*3), 1.30 (s, 3H, -C*H*3), 1.19 (s, 9H, -C(C*H*3)3) ppm; **$^{13}$C NMR** (101 MHz, $CDCl_3$) δ = 179.52 (q, -O*C*(O)C($CH_3$)3), 137.72 (q, 8-C), 128.05 (t, 9-C), 100.62 (q, BDA), 100.22 (q, BDA), 69.39 (t, 11-C), 67.45, 67.36 (t, 2C, 14-C, 12-C), 64.70 (s, 1´-C), 48.21 (p, -O*C*H3), 48.16 (p, -O*C*H3), 39.17 (q, -*C*($CH_3$)3), 35.69 (s, 13-C), 27.46 (p, 3C, -C(*C*H3)3), 18.06 (p, -*C*H3), 18.03 (p, -*C*H3) ppm; **HRMS-ESI** ($C_{18}H_{30}O_7$): calc. for $[M+Na]^+$: 381.18837, found: 381.18822.

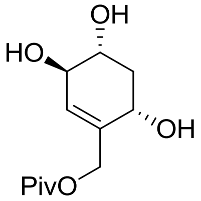

**S7**

**Triol S7.** Alcohol **21** (380 mg, 1.06 mmol) was solved in a mixture of $H_2O$ (3.2 ml) and TFA (9.7 ml). The solution was stirred for 5 min at room temperature and then evaporated under reduced pressure. Purification by flash chromatography on silica (EtOAc) gave **S7** (187 mg, 72%) as a white solid.

$\mathbf{R}_f$ = 0.18 (EtOAc); $[\alpha]_D^{20}$ = –29.0 (*c* = 1.06 in MeOH); **$^1$H NMR** (400 MHz, $CD_3OD$) δ = 5.67 – 5.64 (m, 1H, 9-H), 4.78 – 4.72 (m, 1H, 1´-$H_a$), 4.64 – 4.58 (m, 1H, 1´-$H_b$), 4.40 – 4.34 (m, 1H, 14-H), 4.08 – 4.02 (m, 1H, 11-H), 3.57 (ddd, *J* = 12.1, 7.5, 3.4 Hz, 1H, 12-H), 2.30 (ddd, *J* = 12.2, 5.7, 3.4 Hz, 1H, 13-H), 1.68 (ddd, *J* = 12.1, 12.2, 9.5 Hz, 1H, 13-H), 1.26 (s, 9H, OC(C*H*$_3$)$_3$)) ppm; **$^{13}$C NMR** (101 MHz, $CD_3OD$) δ = 179.68 (q, -O*C*(O)C($CH_3$)$_3$), 139.00, 128.64 (8-C, 9-C), 73.76, 72.69, 67.59, 64.80 (11-C, 12-C, 14-C, 1´-C), 40.45, 39.89 (13-C, -OC(O)*C*($CH_3$)$_3$), 27.57 (p, 3C, -OC(O)C(*C*H$_3$)$_3$) ppm; **HRMS-ESI** ($C_{12}H_{20}O_5$): calc. for $[M+H]^+$: 245.13835, found: 245.13842.

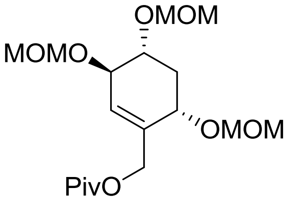

**S8**

***Tris*-MOM Ether S8**. To a solution of triol **S7** (88 mg, 0.36 mmol) in $CH_2Cl_2$ (5 ml) were added DIPEA (502 µl, 2.88 mmol) and MOMCl (219 µl, 2.88 mmol). The reaction mixture was heated under reflux for 15 h. Water (10 ml) was added and the resulting two layers were separated. The aqueous layer was extracted with $CH_2Cl_2$ (3 × 10 ml) and the combined organic layers were dried ($MgSO_4$) and evaporated under reduced pressure. Purification by flash chromatography on silica (petroleum ether/EtOAc 4:1) gave **S8** (84 mg, 62%) as a colourless oil.

**R$_f$** = 0.25 (petroleum ether:EtOAc = 4:1); $[\alpha]_D^{20}$ = –12.6 (*c* = 0.74 in $CH_2Cl_2$); **$^1$H NMR** (400 MHz, $CDCl_3$) δ = 5.75 – 5.72 (m, 1H, 9-H), 4.80 (d, *J* = 6.8 Hz, 1H, -O$CH_2$OCH$_3$), 4.73 – 4.69 (m, 4H, 2 x -O$CH_2$OCH$_3$), 4.62 (d, *J* = 7.0 Hz, 1H, O$CH_2$OCH$_3$), 4.60 (bd, *J* = 7.8 Hz, 2H, 1´-H$_a$, 1´-H$_b$), 4.32 – 4.25 (m, 1H, 14-H), 4.19 – 4.13 (m, 1H, 11-H), 3.68 (ddd, *J* = 11.7, 7.6, 3.6 Hz, 1H, 12-H), 3.39 (s, 3H, -O$CH_3$), 3.37 (s, 3H, -O$CH_3$), 3.36 (s, 3H, -O$CH_3$), 2.48 (ddd, *J* = 12.3, 5.7, 3.6 Hz, 1H, 13-H$_a$), 1.72 (ddd, *J* = 12.3, 11.7, 9.2 Hz, 1H, 13-H$_b$), 1.20 (s, 9H, -OC($CH_3$)$_3$) ppm; **$^{13}$C NMR** (101 MHz, $CDCl_3$) δ = 178.13 (q, -O*C*(O)C(CH$_3$)$_3$), 136.64 (q, 8-C), 127.09 (t, 9-C), 97.05 (s, -O*C*H$_2$OCH$_3$), 96.19 (s, -O*C*H$_2$OCH$_3$), 96.03 (s, -*C*H$_2$OCH$_3$), 77.19 (t, 11-C), 75.52 (t, 12-C), 72.44 (t, 14-C), 63.79 (s, 1´-C), 55.99 (p, -O*C*H$_3$), 55.75 (p, -O*C*H$_3$), 55.64 (p, -O*C*H$_3$), 39.09 (q, -OC(O)*C*(CH$_3$)$_3$), 35.08 (s, 13-C), 27.46 (p, 3C, -OC(O)C($CH_3$)$_3$), **HRMS-ESI** ($C_{18}H_{32}O_8$): calc. for $[M+NH_4]^+$: 394.24354, found: 394.24323.

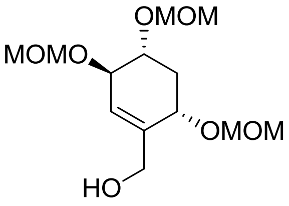


**22**

**Alcohol 22.** To a stirred solution of pivalate **S8** (76 mg, 0.20 mmol) in $CH_2Cl_2$ (5 ml) at –78 °C was added DIBAL-H (1 M in toluene, 1.21 ml, 1.21 mmol). The reaction mixture was allowed to warm to room temperature and stirred for 2 h. Aqueous saturated K-, Na-tartrate-solution (10 ml) and EtOAc (10 ml) were added and the resulting solution was stirred for 2 h at room temperature. The two layers were separated and the aqueous layer was extracted with $CH_2Cl_2$ (3 × 10 ml). The combined organic layers were dried ($MgSO_4$) and evaporated under reduced pressure. Purification by flash chromatography on silica (petroleum ether/EtOAc 1:2 to EtOAc) gave **22** (59 mg, quant.) as a colourless oil.

**R$_f$** = 0.19 (petroleum ether:EtOAc = 1:2); $[\alpha]_D^{20}$ = +4.4 (*c* = 1.24 in $CHCl_3$); **$^1$H NMR** (400 MHz, $CDCl_3$) δ = 5.71 – 5.68 (m, 1H, 9-H), 4.81 – 4.64 (m, 6H, 3 x -O$CH_2$OCH$_3$), 4.41 – 4.34 (m, 1H, 14-H), 4.21 – 4.08 (m, 3H, 11-H, 1´-H$_a$, 1´-H$_b$), 3.66 (ddd, *J* = 12.1, 7.7, 3.6 Hz, 1H, 12-H), 3.40 (s, 3H, -O$CH_3$), 3.38 (s, 3H, -O$CH_3$), 3.37 (s, 3H, -O$CH_3$), 2.47 (ddd, *J* = 12.3, 5.6, 3.6 Hz, 1H, 13-H$_a$), 1.71 (ddd, *J* = 12.1, 12.3, 9.5 Hz, 1H, 13-H$_b$) ppm; **$^{13}$C NMR** (101 MHz, $CDCl_3$) δ = 140.62 (q, 8-C), 126.50 (t, 9-C), 96.88 (s, -*C*H$_2$-OMe),

96.16 (s, -$CH_2$-OMe), 96.11 (s, -$CH_2$-OMe), 77.11 (t, 11-C), 76.03 (t, 12-C), 73.81 (t, 14-C), 64.13 (s, 1´-C), 56.05 (p, -O$CH_3$), 55.74 (p, -O$CH_3$), 55.65 (p, -O$CH_3$), 35.09 (s, 13-C) ppm; **HRMS-ESI** ($C_{13}H_{24}O_7$): calc. for $[M+Na]^+$: 315.14142, found: 315.14158.

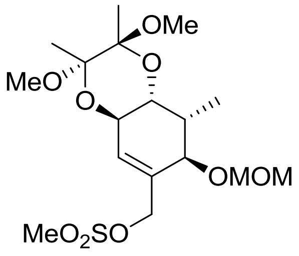


**S9**

**Mesylate S9.** To a stirred solution of alcohol **19** (39 mg, 0.12 mmol) in $CH_2Cl_2$ (3 ml) at –10 °C were added $NEt_3$ (81 µl, 0.59 mmol) and MsCl (18 µl, 0.23 mmol). The reaction mixture was stirred for 30 min at –10 °C and 40 min at room temperature. $Et_2O$ (5 ml) and aqueous saturated $NaHCO_3$-solution (10 ml) were added and the resulting two layers were separated. The aqueous layer was extracted with $CH_2Cl_2$ (2 × 10 ml) and the combined organic layers were dried ($MgSO_4$) and evaporated under reduced pressure. Purification by flash chromatography on silica (cyclohexane/EtOAc 1:1) gave **S9** (42 mg, 88%) as a white solid.

$\mathbf{R_f}$ = 0.13 (petroleum ether:EtOAc = 1:1); $[\alpha]_D^{20}$ = +62.1 ($c$ = 1.10 in $CH_2Cl_2$); $^1$**H NMR** (400 MHz, $CDCl_3$) δ = 5.88 (bs, 1H, 9-H), 4.81 – 4.76 (m, 1H, 1´-$H_a$), 4.70 (d, $J$ = 7.0 Hz, 1H, -O$CH_2$O$CH_3$), 4.65 (d, $J$ = 7.0 Hz, 1H, O$C\mathit{H}_2$O$CH_3$), 4.59 (d, $J$ = 11.6 Hz, 1H, 1´-$H_b$), 4.23 (d, $J$ = 9.3 Hz, 1H, 11-H), 3.93 (dd, $J$ = 9.3, 4.2 Hz, 1H, 12-H), 3.84 (bs, 1H, 14-H), 3.37 (s, 3H, -O$CH_2$O$C\mathit{H}_3$), 3.23 (s, 3H, -O$C\mathit{H}_3$), 3.20 (s, 3H, -O$C\mathit{H}_3$), 2.99 (s, 3H, -S$(O)_2$C$\mathit{H}_3$), 2.32 – 2.24 (m, 1H, 13-H), 1.28 (s, 3H, -C$\mathit{H}_3$), 1.27 (s, 3H, -C$\mathit{H}_3$), 0.94 (d, $J$ = 7.4 Hz, 3H, 2´-H) ppm; $^{13}$**C NMR** (101 MHz, $CDCl_3$) δ = 132.02 (q, 8-C), 131.17 (t, 9-C), 100.67 (q, BDA), 100.44 (q, BDA), 97.10 (s, -$CH_2$-OMe), 77.55, 70.14, 67.37, 64.69 (11-C, 12-C, 14-C, 1´-C), 56.12 (p, -O$CH_3$), 48.08 (p, -O$CH_3$), 47.86 (p, -O$CH_3$), 38.04, 35.60 (13-C, -$SO_2CH_3$), 18.02 (p, 2C, -$CH_3$), 10.85 (p, 2´-C) ppm; **HRMS-ESI** ($C_{17}H_{30}O_9S$): calc. for $[M+Na]^+$: 433.15027, found: 433.14994; **m.p.:** 101.1 °C.

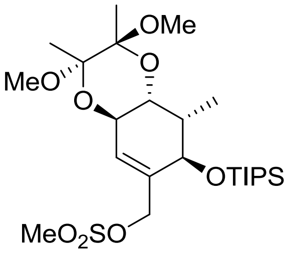


**S10**

**Mesylate S10.** To a solution of alcohol **20** (50 mg, 0.11 mmol) in $CH_2Cl_2$ (3 ml) was added $NEt_3$ (78 µl, 0.56 mmol). The solution was cooled to –10 °C and subsequently MsCl (17 µl, 0.22 mmol) was added. The reaction mixture was stirred for 30 min at –10 °C and 20 min at room temperature. $Et_2O$ (5 ml) and aqueous saturated $NaHCO_3$-solution (5 ml) were added and the resulting two layers were separated. The aqueous layer was extracted with $CH_2Cl_2$ (10 ml) and the combined organic layers were dried ($MgSO_4$) and evaporated under reduced pressure. Purification by flash chromatography on silica (petroleum ether/EtOAc 5:1) gave mesylate **S10** (59 mg, quant.) as a colourless oil.

**$R_f$** = 0.18 (petroleum ether:EtOAc = 5:1); $[\alpha]_D^{20}$ = +46.5 (*c* = 1.56 in $CH_2Cl_2$); **$^1$H NMR** (500 MHz, $CDCl_3$) δ = 5.81 (bs, 1H, 9-H), 4.77 (d, *J* = 11.6 Hz, 1H, 1´-$H_a$), 4.64 (d, *J* = 11.6 Hz, 1H, 1´-$H_b$), 4.24 (d, *J* = 9.5 Hz, 1H, 11-H), 4.16 (bs, 1H, 14-H), 4.05 (dd, *J* = 9.5, 4.0 Hz, 1H, 12-H), 3.22 (s, 3H, -OC$H_3$), 3.21 (s, 3H, -OC$H_3$), 2.96 (s, 3H, -OS(O)C$H_3$), 2.18 – 2.11 (m, 1H, 13-H), 1.29 (s, 3H, -C$H_3$), 1.27 (s, 3H, -C$H_3$), 1.08 – 1.03 (m, 21H, -Si(C*H*(C$H_3$)$_2$)$_3$), 0.93 (d, *J* = 7.4 Hz, 3H, 2´-H) ppm; **$^{13}$C NMR** (126 MHz, $CDCl_3$) δ = 133.96 (q, 8-C), 129.45 (t, 9-C), 100.67 (q, BDA), 100.45 (q, BDA), 72.19 (t, 14-C), 70.49 (s, 1´-C), 67.19 (t, 12-C), 64.98 (t, 11-C), 48.16 (p, -O*C*$H_3$), 48.08 (p, -O*C*$H_3$), 39.09 (t, 13-C), 38.15 (p, -OC(O)*C*$H_3$), 18.37 (p, -*C*$H_3$), 18.33 (p, -*C*$H_3$), 18.12 (t, -Si(*C*H(C$H_3$)$_2$)$_3$), 18.04 (t, -Si(*C*H(C$H_3$)$_2$)$_3$), 12.90 (6 x p, -Si(CH(*C*$H_3$)$_2$)$_3$), 10.72 (p, 2´-C) ppm; (1 x -Si(*C*H(C$H_3$)$_2$)$_3$ is missing); **HRMS-ESI** ($C_{24}H_{46}O_8SSi$): calc. for $[M+Na]^+$: 545.25749, found: 545.25696.

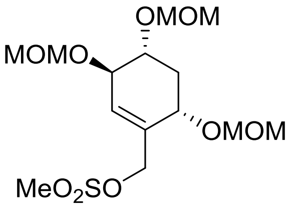

**S11**

**Mesylate S11.** To a solution of alcohol **22** (10 mg, 33 µmol) in $CH_2Cl_2$ (1 ml) at –10 °C were added $NEt_3$ (23 µl, 0.16 mmol) and MsCl (5.1 µl, 65 µmol). The reaction mixture was stirred for 30 min at –10 °C and 1 h at room temperature. $Et_2O$ (10 ml) and aqueous saturated $NaHCO_3$-solution (10 ml) were added and the resulting two layers were separated. The aqueous layer was extracted with $CH_2Cl_2$ (2 × 10 ml) and the combined organic layers were dried ($MgSO_4$) and evaporated under reduced pressure. Purification by flash chromatography on silica (EtOAc/cyclohexane 2:1) gave mesylate **S11** (8 mg, 64%) as a colourless oil.

**R*f*** = 0.28 (petroleum ether:EtOAc = 1:1); **$^1$H NMR** (400 MHz, $CDCl_3$) δ = 5.87 – 5.84 (m, 1H, 9-H), 4.86 – 4.61 (m, 8H, -OC$H_2$OC$H_3$, 1´-$H_a$, 1´-$H_b$), 4.42 – 4.37 (m, 1H, 14-H), 4.16 (bd, *J* = 8.2 Hz, 1H, 11-H), 3.68 (dd, *J* = 8.2, 3.8 Hz, 1H, 12-H), 3.41 (s, 3H, -OCH$_2$OC$H_3$), 3.39 (s, 3H, -OCH$_2$OC$H_3$), 3.37 (s, 3H, -OCH$_2$OC$H_3$), 3.00 (s, 3H, -OS(O)C$H_3$), 2.67 – 2.57 (m, 1H, 13-H), 0.94 (d, *J* = 7.0 Hz, 3H, 2´-H) ppm; **$^{13}$C NMR** (101 MHz, $CDCl_3$) δ = 133.38 (q, 8-C), 129.03 (t, 9-C), 97.15 (s, -O*C*H$_2$OCH$_3$), 96.21 (s, -O*C*H$_2$OCH$_3$), 96.04 (s, -O*C*H$_2$OCH$_3$), 78.62 (t, 12-C), 74.58, 74.42 (t, 2C, 11-C, 14-C), 69.53 (s, 1´-C), 56.47 (p, -O*C*H$_3$), 55.79 (p, -O*C*H$_3$), 55.75 (p, -O*C*H$_3$), 38.21 (p, -OS(O)*C*H$_3$), 36.69 (t, 13-C), 7.58 (p, 2´-C) ppm; **HRMS-ESI** ($C_{15}H_{28}O_9S$): calc. for $[M+NH_4]^+$: 402.17923, found: 402.17929.

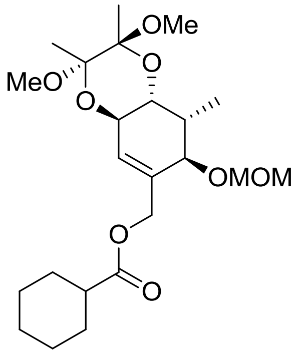


**27**

**Ester 27.** To a solution of alcohol **19** (100 mg, 300 µmol) in $CH_2Cl_2$ (4 ml) were added DMAP (cat.), DCC (127 mg, 614 µmol) and cyclohexanecarboxylic acid (66 mg, 0.51 mmol). The reaction mixture was stirred for 4 h at room temperature and then concentrated under reduced pressure. Purification by flash chromatography on silica (petroleum ether/EtOAc 7:1) gave ester **27**, which was still contaminated with urea and used in the next step without further purification.

$\mathbf{R}_{\boldsymbol{f}}$ = 0.50 (petroleum ether:EtOAc = 4:1); $^{1}$**H NMR** (400 MHz, $CDCl_3$) δ = 5.77 – 5.74 (m, 1H, 9-H), 4.70 (d, *J* = 7.1 Hz, 1H, -OC$H_2$OCH$_3$,), 4.64 – 4.60 (m, 1H, 1´-H$_a$), 4.61 (d, *J* = 7.1 Hz, 1H, -OC$H_2$OCH$_3$), 4.52 – 4.48 (m, 1H, 1´-H$_b$), 4.22 (d, *J* = 9.4 Hz, 1H, 11-H), 3.96 (dd, *J* = 9.4, 4.2 Hz, 1H, 12-H), 3.79 (bs, 1H, 14-H), 3.36 (s, 3H, -OCH$_2$OC$H_3$), 3.24 (s, 3H, -OCH$_3$), 3.21 (s, 3H, -OCH$_3$), 2.35 – 2.20 (m, 2H, 13-H, cyclohexyl), 1.92 – 1.84 (m, 2H, cyclohexyl), 1.81 – 1.67 (m, 4H, cyclohexyl), 1.66 – 1.58 (m, 2H, cyclohexyl), 1.49 – 1.36 (m, 2H, cyclohexyl), 1.29 (s, 3H, -C$H_3$), 1.28 (s, 3H, -C$H_3$), 0.94 (d, *J* = 7.4 Hz, 3H, 2´-H) ppm; $^{13}$**C NMR** (101 MHz, $CDCl_3$) δ = 175.90 (q, -O*C*(O)R), 133.72 (q, 8-C), 128.02 (t, 9-C), 100.65 (q, BDA), 100.44 (q, BDA), 96.82 (s, -*C*H$_2$-OMe), 77.68 (t, 14-C), 67.67 (t, 12-C), 64.91 (s, 1´-C), 64.69 (t, 11-C), 56.08 (p, -O*C*H$_3$), 48.07 (p, -O*C*H$_3$), 47.85 (p, -O*C*H$_3$), 43.44 (t, 13-C), 35.68 (t, cyclohexyl), 29.28 (s, cyclohexyl), 29.26 (s, cyclohexyl), 25.95 (s, cyclohexyl), 25.66 (s, 2C, cyclohexyl), 18.09 (p, 2C, -*C*H$_3$), 10.95 (p, 2´-C) ppm; **HRMS-ESI** ($C_{23}H_{38}O_8$): calc. for $[M+NH_4]^+$: 460.29049, found: 460.29016.

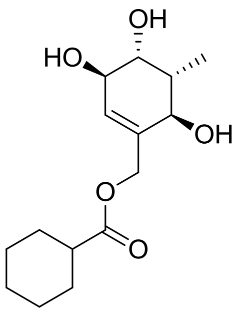


**28**

**Triol 28.** Ester **27** (83 mg, 0.19 mmol) was solved in a mixture of $H_2O$ (0.5 ml) and TFA (1.5 ml). The reaction mixture was stirred for 30 min at room temperature and then evaporated under reduced pressure. Purification by flash chromatography on silica (EtOAc) gave triol **28** (39 mg, 73% over 2 steps) as a white solid.

$\mathbf{R_f}$ = 0.18 (EtOAc); $[\alpha]_D^{20}$ = –1.3 (*c* = 0.4 in EtOAc); **$^1$H NMR** (400 MHz, $CDCl_3$) δ = 5.74 – 5.71 (m, 1H, 9-H), 4.74 – 4.69 (m, 1H, 1´-$H_a$), 4.58 – 4.53 (m, 1H, 1´-$H_b$), 4.07 (bd, *J* = 7.5 Hz, 1H, 11-H), 3.93 (dd, *J* = 7.5, 3.9 Hz, 1H, 12-H), 3.90 (d, *J* = 3.2 Hz, 1H, 14-H), 2.38 – 2.25 (m, 2H, 13-H, cyclohexyl), 2.23 – 2.12 (m, 1H, cyclohexyl), 1.93 – 1.84 (m, 2H, cyclohexyl), 1.77 – 1.69 (m, 2H, cyclohexyl), 1.66 – 1.58 (m, 1H, cyclohexyl), 1.49 – 1.35 (m, 4H, cyclohexyl), 0.93 (d, *J* = 7.3 Hz, 3H, 2´-H) ppm; **$^{13}$C NMR** (101 MHz, $CDCl_3$) δ = 176.63* (q, -*C*(O)OR), 135.68 (q, 8-C), 128.70 (t, 9-C), 72.33 (t, 14-C), 71.33 (t, 12-C), 69.89 (t, 11-C), 65.15 (s, 1´-C), 43.43 (t, 13-C), 40.32 (t, cyclohexyl), 29.94 (s, cyclohexyl), 29.26 (s, cyclohexyl), 25.91 (s, cyclohexyl), 25.62 (s, 2C, cyclohexyl), 11.04 (p, 2´-C); **HRMS-ESI** ($C_{15}H_{24}O_5$): calc. for $[M+H]^+$: 285.16965, found: 285.16956.

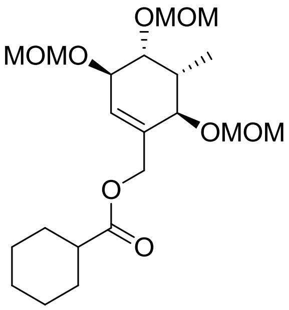


**29**

**Ester 29 [1].** To a solution of triol **28** (23 mg, 0.08 mmol) in $CH_2Cl_2$ (5 ml) were added DIPEA (0.16 ml, 0.88 mmol) and MOMCl (67 µl, 0.88 mmol). The reaction mixture was

heated under reflux for 15 h. $H_2O$ (10 ml) was added and the resulting two layers were separated. The aqueous layer was extracted with $CH_2Cl_2$ (3 × 10 ml) and the combined organic layers were dried ($MgSO_4$) and evaporated under reduced pressure. Purification by flash chromatography on silica (petroleum ether/EtOAc 3:1) gave MOM ether **29** (29 mg, 86%) as a colourless oil.

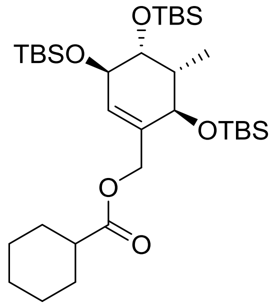


**30**

**Ester 30 [1].** To a solution of triol **28** (28 mg, 0.10 mmol) in $CH_2Cl_2$ (2 ml) were added TBSCl (74 mg, 0.49 mmol) and imidazole (40 mg, 0.58 mmol). The reaction mixture was stirred for 21 h at room temperature. 1 M HCl (5 ml) and EtOAc (5 ml) were added and the two layers were separated. The aqueous layer was extracted with EtOAc (2 × 10 ml) and the combined organic layers were washed with brine, dried ($MgSO_4$) and evaporated under reduced pressure. Purification by flash chromatography on silica (petroleum ether/EtOAc 30:1) gave silylether **30** (28 mg, 45%) as a colourless oil

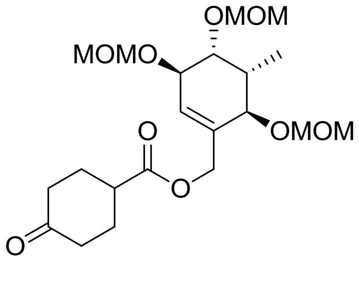


**43**

**4-Oxocyclohexylcarboxylate 43.** To a stirred solution of alcohol **40** (293 mg, 0.96 mmol) in $CH_2Cl_2$ (10 ml) were added EDC·HCl (238 mg, 1.24 mmol), DMAP (29 mg, 0.24 mmol) and 4-oxocyclohexanecarboxylic acid (163 mg, 1.15 mmol). The reaction mixture was stirred for

19 h at room temperature. The organic layer was washed with 1 M HCl (1 × 10 ml), aqueous saturated $NaHCO_3$-solution (10 ml) and water (2 × 10 ml), dried ($MgSO_4$) and evaporated under reduced pressure. Purification by flash chromatography on silica (petroleum ether/EtOAc 1:1) gave ester **43** (360 mg, 87%) as a colourless oil.

**$R_f$** = 0.33 (petroleum ether:EtOAc = 1:2); $[\alpha]_D^{20}$ = –1.8 (*c* = 1.0 in $CH_2Cl_2$); **$^1$H NMR** (500 MHz, $CDCl_3$) δ = 5.79 – 5.74 (m, 1H, 9-H), 4.72 (d, *J* = 6.8 Hz, 1H, -OC*H*$_2$OCH$_3$), 4.69 – 4.61 (m, 5H, -OC*H*$_2$OCH$_3$, 1´-H$_a$), 4.58 (d, *J* = 7.1 Hz, 1H, -OC*H*$_2$OCH$_3$), 4.53 (d, *J* = 12.9 Hz, 1H, 1´-H$_b$), 4.02 (bd, *J* = 7.7 Hz, 1H, 11-H), 3.87 (dd, *J* = 7.7, 4.0 Hz, 1H, 12-H), 3.76 (d, *J* = 3.1 Hz, 1H, 14-H), 3.32 (s, 3H, -OC*H*$_3$), 3.32 (s, 3H, -OC*H*$_3$), 3.31 (s, 3H, -OC*H*$_3$), 2.77 – 2.68 (m, 1H, cyclohexyl), 2.44 – 2.23 (m, 5H, cyclohexyl, 13-H), 2.19 – 2.10 (m, 2H, cyclohexyl), 2.01 – 1.89 (m, 2H, cyclohexyl), 0.88 (d, *J* = 7.2 Hz, 3H, 2´-H) ppm; **$^{13}$C NMR** (126 MHz, $CDCl_3$) δ = 209.68 (q, *C*=O), 173.69 (q, -*C*(O)OR), 133.48 (q, 8-C), 128.91 (t, 9-C), 96.98 (s, -O*C*H$_2$OCH$_3$), 96.82 (s, -O*C*H$_2$OCH$_3$), 96.49 (s, -O*C*H$_2$OCH$_3$), 77.16 (t, 14-C), 76.98 (t, 12-C), 73.99 (t, 11-C), 65.12 (s, 1´-C), 56.01 (p, -O*C*H$_3$), 55.51 (p, -O*C*H$_3$), 55.50 (p, -O*C*H$_3$), 40.80 (t, cyclohexyl), 39.74 (s, 2C, cyclohexyl), 36.26 (t, 13-C), 28.60 (s, 2C, cyclohexyl), 11.38 (p, 2´-C) ppm; **HRMS-ESI** ($C_{21}H_{34}O_9$): calc. for $[M+Na]^+$: 453.20950, found: 453.20905.

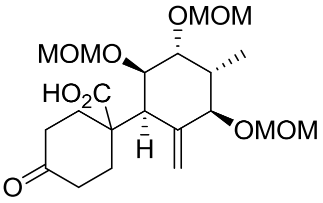


**44a**

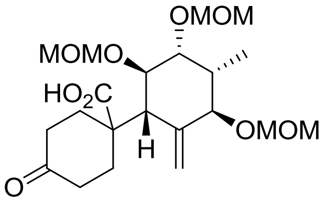


**44b**

**Carboxylic acids 44a and 44b.** To a solution of ester **43** (32 mg, 74 µmol) in a Schlenk-flask in toluene (2 ml) at –78 °C was slowly added KHMDS (0.5 M in toluene, 372 µl, 186 µmol). The reaction mixture was stirred for 2 h at –78 °C and then TMSCl (28 µl, 0.22 mmol) was added. After having stirred for 5 min at –78 °C, the reaction mixture was allowed to warm to room temperature. After having stirred for 15 min at room temperature, the Schlenk-flask is sealed and the reaction mixture was heated for 22.5 h at 85 °C. The reaction mixture was cooled to room temperature and then 1 M HCl (10 ml) was added. The solution was stirred for 30 min at room temperature and then the two layers were separated. The aqueous layer was extracted with EtOAc (3 × 10 ml) and the combined organic layers were washed with brine

(20 ml), dried ($MgSO_4$) and evaporated under reduced pressure. Purification by flash chromatography on silica (petroleum ether/EtOAc 2:1; 0.1% acetic acid) gave carboxylic acids **44a** (*major diastereomer*) and **44b** (*minor diastereomer*) (25 mg, 75% combined yield) as an inseparable mixture of diastereomers (de = 80%, calculated from the $^1$H-NMR in $CDCl_3$).

**44a** *(major diastereomer):* **$R_f$** = 0.19 (petroleum ether:EtOAc = 1:1); **$^1$H NMR** (600 MHz, $CDCl_3$) δ = 10.67 (bs, 1H, -$CO_2$*H*), 5.26 (bs, 1H, 1´-$H_a$), 4.92 (bs, 1H, 1´-$H_b$), 4.66 – 4.55 (m, 5H, -OC*H*$_2$OCH$_3$), 4.53 (d, *J* = 7.0 Hz, 1H, -OC*H*$_2$OCH$_3$), 4.04 (d, *J* = 11.2 Hz, 1H, 14-H), 3.80 (bs, 1H, 11-H), 3.70 (dd, *J* = 7.1 Hz, 1H, 12-H), 3.36 (s, 3H, -OC*H*$_3$), 3.35 (s, 3H, -OC*H*$_3$), 3.28 (s, 3H, -OC*H*$_3$), 2.84 (bs, 1H, 9-H), 2.58 – 2.53 (m, 1H, cyclohexyl), 2.52 – 2.40 (m, 2H, cyclohexyl), 2.36 – 2.24 (m, 3H, cyclohexyl), 2.09 – 2.00 (m, 1H, cyclohexyl), 2.00 – 1.95 (m, 1H, 13-H), 1.76 – 1.65 (m, 1H cyclohexyl), 1.04 (d, *J* = 6.9 Hz, 3H, 2´-H) ppm; **$^{13}$C NMR** (151 MHz, $CDCl_3$) δ = 211.47 (q, *C*=O), 179.50 (q, -*C*O$_2$H), 143.33 (q, 8-C), 114.74 (s, 1´-C), 96.16 (s, -O*C*H$_2$OCH$_3$), 95.73 (s, -O*C*H$_2$OCH$_3$), 95.64 (s, -O*C*H$_2$OCH$_3$), 78.99 (t, 12-C), 77.69 (t, 14-C), 73.92 (t, 11-C), 56.28, 56.18, 55.93 (2C) (-O*C*H$_3$, 9-C), 49.47 (q, cyclohexyl), 38.94 , 38.77, 38.61, 33.53, 31.49 (cyclohexyl, 13-C), 14.27 (p, 2´-C) ppm; **HRMS-ESI** ($C_{21}H_{34}O_9$): calc. for $[M+H]^+$: 431.22756, found: 431.22718.

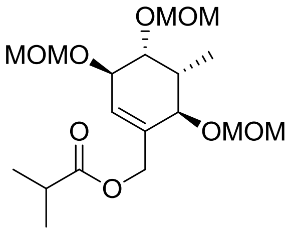


**45**

**Isobutyrate 45.** Ester **45** was prepared from alcohol **40** according to the *experimental procedure for the preparation of ester **43*** (81% yield).

**$R_f$** = 0.50 (petroleum ether:EtOAc = 3:1); $[\alpha]_D^{20}$ = +0.83 (*c* = 1.45 in $CHCl_3$); **$^1$H NMR** (400 MHz, $CDCl_3$) δ = 5.82 – 5.80 (m, 1H, 9-H), 4.85 – 4.51 (m, 8H, -OC*H*$_2$OCH$_3$, 1´-H), 4.08 (bd, *J* = 7.8 Hz, 1H, 11-H), 3.95 (dd, *J* = 7.8, 4.0 Hz, 1H, 12-H), 3.82 (d, *J* = 3.0 Hz, 1H, 14-H), 3.39 (s, 6H, -OCH$_2$OC*H*$_3$), 3.37 (s, 3H, -OCH$_2$OC*H*$_3$), 2.62 – 2.51 (m, 1H, -C*H*(CH$_3$)$_2$), 2.49 – 2.37 (m, 1H, 14-H), 1.17 (d, *J* = 2.0 Hz, 3H, -CH(C*H*$_3$)$_2$), 1.15 (d, *J* = 2.0 Hz, 3H, -CH(C*H*$_3$)$_2$), 0.94 (d, *J* = 7.2 Hz, 3H, 2´-H) ppm; **$^{13}$C NMR** (101 MHz, $CDCl_3$)

δ = 176.81 (q, -*C*(O)OR), 133.80 (q, 8-C), 128.50 (t, 9-C), 97.11 (s, -O*C*$H_2OCH_3$), 96.95 (s, -O*C*$H_2OCH_3$), 96.64 (s, -O*C*$H_2OCH_3$), 77.28 (t, 14-C), 77.23 (t, 12-C), 74.28 (t, 11-C), 64.72 (s, 1´-C), 56.11 (p, -OC*$H_3$*), 55.65 (p, -OC*$H_3$*), 55.64 (p, -OC*$H_3$*), 36.46 (t, 13-C), 34.27 (t, -*C*$H(CH_3)_2$), 19.22 (p, 2C, -CH(*C*$H_3)_2$), 11.38 (p, 2´-C) ppm; **HRMS-ESI** ($C_{18}H_{32}O_8$): calc. for $[M+Na]^+$: 399.19894, found: 399.19826.

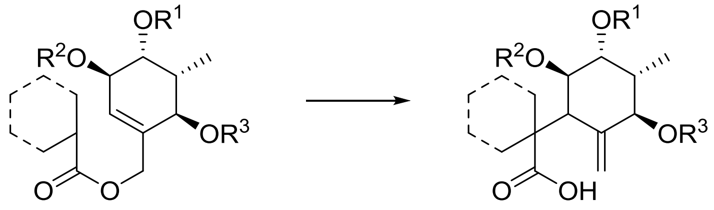


**A** **B**

**General procedure for the rearrangement of 29, 30, 45, 47, 49, 52, 54:** To a stirred solution of ester **A** (1.0 eq.) in toluene (14 ml/mmol) in a Schlenk tube at –78 °C was added KHMDS (0.5 M in toluene, 2.0 equiv). The resulting solution was stirred at –78°C for 2 h and then TMSCl (2.5 equiv) was added. The reaction mixture was stirred for 15 min at –78 °C and then allowed to warm to room temperature. After stirring for 10 min at room temperature, the Schlenk tube was sealed and warmed to 85 °C for 20 h. The reaction mixture was cooled to room temperature and then quenched with 1 M HCl. The aqueous layer was extracted with EtOAc (3×). The combined organic layers were washed with brine, dried ($MgSO_4$) and evaporated under reduced pressure to give the crude product. Purification by flash chromatography on silica gave carboxylic acids **B** as an inseparable mixture of diastereomers (the diastereomeric ratio is determined from the $^1$H NMR).

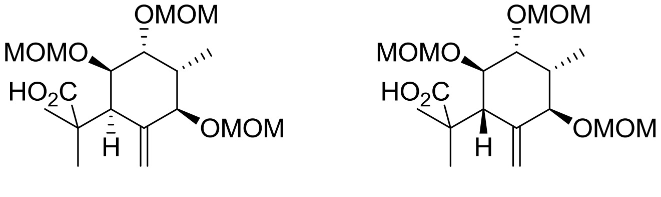


**46a** **46b**

**Carboxylic acids 46a and 46b** were prepared from ester **45** according to the *general procedure for the rearrangement of* ***29, 30, 45, 47, 49, 52, 54*** (93% yield, de = 78%).

**46a** *(major diastereomer):* **R$_f$** = 0.51 (petroleum ether:EtOAc = 1:1); **$^1$H NMR** (600 MHz, $CDCl_3$) δ = 10.67 (bs, 1H, -$CO_2$*H*), 5.17 (s, 1H, 1´-H$_a$), 5.00 (s, 1H, 1´-H$_b$), 4.69 – 4.63 (m, 4H, -OC*H*$_2$OCH$_3$), 4.61 – 4.55 (m, 2H, -OC*H*$_2$OCH$_3$), 4.00 (d, *J* = 7.5 Hz, 1H, 14-H), 3.80 (dd, *J* = 9.5 Hz, 1H, 12-H), 3.64 (dd, *J* = 10.7 Hz, 1H, 11-H), 3.37 (s, 3H, -OC*H*$_3$), 3.36 (s, 3H, -OC*H*$_3$), 3.32 (s, 3H, -OC*H*$_3$), 2.98 (d, *J* = 5.4 Hz, 1H, 9-H), 2.17 – 2.09 (m, 1H, 13-H), 1.30 (d, *J* = 4.0 Hz, 3H, -C*H*$_3$), 1.30 (d, *J* = 4.0 Hz, 3H, -C*H*$_3$), 0.99 (d, *J* = 7.0 Hz, 3H, 2´-H) ppm; **$^{13}$C NMR** (151 MHz, $CDCl_3$) δ = 183.88 (q, -*C*O$_2$H), 143.29 (q, 8-C), 114.64 (s, 1´-C), 96.78 (s, 2C, -O*C*H$_2$OCH$_3$), 94.96 (s, -O*C*H$_2$OCH$_3$), 80.40 (t, 12-C), 79.52 (t, 14-C), 76.34 (t, 11-C), 56.37 (p, -O*C*H$_3$), 56.16 (p, -O*C*H$_3$), 56.04 (p, -O*C*H$_3$), 52.37 (t, 9-C), 44.57 (q, alkyl), 38.81 (t, 13-C), 24.64 (p, 2C -*C*H$_3$), 13.48 (p, 2´-C) ppm; **HRMS-ESI** ($C_{18}H_{32}O_8$): calc. for $[M+H]^+$: 377.21699, found: 377.21695.

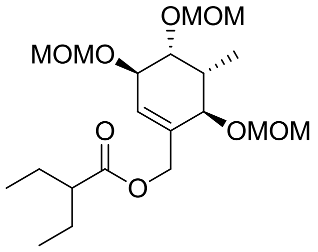


**47**

**Ester 47.** Ester **47** was prepared from alcohol **40** according to the *experimental procedure for the preparation of ester **43*** (93% yield).

**R$_f$** = 0.50 (petroleum ether:EtOAc = 3:1); $[\alpha]_D^{20}$ = +8.8 (*c* = 1.0 in $CHCl_3$); **$^1$H NMR** (400 MHz, $CDCl_3$) δ = 5.83 – 5.79 (m, 1H, 9-H), 4.77 (d, *J* = 6.8 Hz, 1H, -OC*H*$_2$OCH$_3$), 4.74 – 4.60 (m, 6H, -OC*H*$_2$OCH$_3$, 1´-H$_a$), 4.54 (d, *J* = 12.9 Hz, 1H, 1´-H$_b$), 4.06 (d, *J* = 8.0 Hz, 1H, 11-H), 3.93 (dd, *J* = 8.0, 4.0 Hz, 1H, 12-H), 3.81 (d, *J* = 3.0 Hz, 1H, 14-H), 3.38 (s, 3H, -OCH$_2$OC*H*$_3$), 3.37 (s, 3H, -OCH$_2$OC*H*$_3$), 3.35 (s, 3H, -OCH$_2$OC*H*$_3$), 2.46 – 2.34 (m, 1H, 13-H), 2.25 – 2.15 (m, 1H, -C*H*(CH$_2$CH$_3$)$_2$), 1.67 – 1.42 (m, 4H, -CH(C*H*$_2$CH$_3$)$_2$), 0.92 (d, *J* = 7.3 Hz, 3H, 2´-H), 0.86 (t, *J* = 7.4 Hz, 3H, -CH(CH$_2$C*H*$_3$)$_2$), 0.86 (t, *J* = 7.4 Hz, 3H, -CH(CH$_2$C*H*$_3$)$_2$) ppm; **$^{13}$C NMR** (101 MHz, $CDCl_3$) δ = 175.92 (q, -*C*(O)OR), 133.83 (q, 8-C), 128.77 (t, 9-C), 97.14 (s, -O*C*H$_2$OCH$_3$), 96.90 (s, -O*C*H$_2$OCH$_3$), 96.60 (s, -O*C*H$_2$OCH$_3$), 77.38 (t, 14-C), 77.16 (t, 12-C), 74.20 (t, 11-C), 64.51 (s, 1´-C), 56.06 (p, -OCH$_2$O*C*H$_3$), 55.59 (p, 2C, -OCH$_2$O*C*H$_3$), 49.15 (t, -*C*H(CH$_2$CH$_3$)$_2$), 36.46 (t, 13-C),

25.18 (s, -CH($CH_2CH_3$)$_2$), 25.17 (s, -CH(*C*$H_2CH_3$)$_2$), 12.04 (p, 2C, -CH($CH_2$*C*$H_3$)$_2$), 11.33 (p, 2´-C) ppm; **HRMS-ESI** ($C_{20}H_{36}O_8$): calc. for $[M+Na]^+$: 427.23024, found: 427.22948.

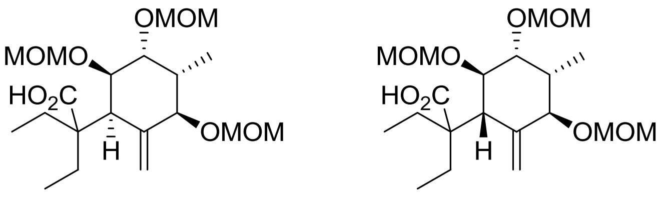


**48a** **48b**

**Carboxylic acids 48a and 48b** were prepared from ester **47** according to the *general procedure for the rearrangement of **29, 30, 45, 47, 49, 52, 54*** (quant. yield, de = 72%).

**48a** *(major diastereomer):* $\mathbf{R_f}$ = 0.32 (petroleum ether:EtOAc = 1:1); $^{1}$**H NMR** (600 MHz, $CDCl_3$) δ = 9.38 (bs, 1H, -$CO_2$*H*), 5.22 (s, 1H, 1´-$H_a$), 4.93 (s, 1H, 1´-$H_b$), 4.76 – 4.56 (m, 5H, -OC*H*$_2$OCH$_3$), 4.49 (d, *J* = 6.9 Hz, 1H, -OC*H*$_2$OCH$_3$), 4.20 (d, *J* = 9.4 Hz, 1H, 14-H), 3.93 – 3.89 (m, 1H, 11-H), 3.75 (dd, *J* = 8.0 Hz, 1H, 12-H), 3.39 (s, 3H, -OC*H*$_3$), 3.35 (s, 3H, -OC*H*$_3$), 3.33 (s, 3H, -OC*H*$_3$), 2.79 (d, *J* = 2.4 Hz, 1H, 9-H), 2.05 – 1.99 (m, 1H, 13-H), 1.98 – 1.71 (m, 3H, -C*H*$_2$CH$_3$), 1.59 – 1.52 (m, 1H, -C*H*$_2$CH$_3$), 1.05 (d, *J* = 6.9 Hz, 3H, 2´-H), 0.85 (t, *J* = 7.5 Hz, 3H, -C*H*$_3$), 0.81 (t, *J* = 7.4 Hz, 3H, -C*H*$_3$) ppm; $^{13}$**C NMR** (151 MHz, $CDCl_3$) δ = 181.80 (q, -*C*O$_2$H), 144.05 (q, 8-C), 113.74 (s, 1´-C), 96.72 (s, -OC*H*$_2$OCH$_3$), 96.32 (s, -OC*H*$_2$OCH$_3$), 95.56 (s, -OC*H*$_2$OCH$_3$), 79.27 (t, 12-C), 78.67 (t, 14-C), 74.50 (t, 11-C), 56.22 (p, -O*C*H$_3$), 56.18 (p, -O*C*H$_3$), 56.09 (p, -O*C*H$_3$), 52.72 (t, 9-C), 50.27 (q, cyclohexyl), 38.70 (t, 13-C), 26.66 (s, -*C*H$_2$CH$_3$), 24.40 (s, -*C*H$_2$CH$_3$), 14.48 (p, 2´-C), 8.87 (-CH$_2$*C*H$_3$), 8.38 (-CH$_2$*C*H$_3$) ppm; **HRMS-ESI** ($C_{20}H_{36}O_8$): calc. for $[M+H]^+$: 405.24829, found: 405.24832.

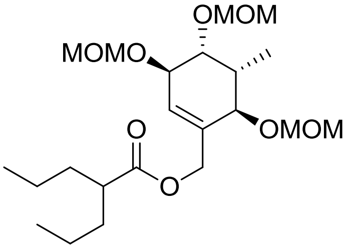


**49**

**Ester 49.** Ester **49** was prepared from alcohol **40** according to the *experimental procedure for the preparation of ester **43*** (quant. yield).

**R*f*** = 0.50 (petroleum ether:EtOAc = 3:1); $[\alpha]_D^{20}$ = +8.8 (*c* = 1.02 in $CHCl_3$); **$^1$H NMR** (400 MHz, $CDCl_3$) δ = 5.82 – 5.79 (m, 1H, 9-H), 4.85 – 4.47 (m, 8H, -OC*H*$_2$OCH$_3$, 1´-H), 4.07 (bd, *J* = 8.0 Hz, 1H, 11-H), 3.93 (dd, *J* = 8.0, 4.0 Hz, 1H, 12-H), 3.80 (d, *J* = 3.0 Hz, 1H, 14-H), 3.38 (s, 3H, -OCH$_2$OC*H*$_3$), 3.37 (s, 3H, -OCH$_2$OC*H*$_3$), 3.35 (s, 3H, -OCH$_2$OC*H*$_3$), 2.47 – 2.30 (m, 2H, -C*H*(CH$_2$CH$_2$CH$_3$)$_2$, 13-H), 1.66 – 1.51 (m, 2H, -CH(C*H*$_2$CH$_2$CH$_3$)$_2$), 1.46 – 1.34 (m, 2H, -CH(C*H*$_2$CH$_2$CH$_3$)$_2$), 1.33 – 1.19 (m, 4H, -CH(CH$_2$C*H*$_2$CH$_3$)$_2$), 0.92 (d, *J* = 7.3 Hz, 3H, 2´-H), 0.86 (t, *J* = 7.2 Hz, 3H, -CH(CH$_2$CH$_2$C*H*$_3$)$_2$), 0.86 (t, *J* = 7.2 Hz, 3H, -CH(CH$_2$CH$_2$C*H*$_3$)$_2$) ppm; **$^{13}$C NMR** (101 MHz, $CDCl_3$) δ = 176.25 (q, -*C*(O)OR), 133.82 (q, 8-C), 128.82 (t, 9-C), 97.16 (s, -*C*H$_2$OCH$_3$), 96.86 (s, -*C*H$_2$OCH$_3$), 96.60 (s, -*C*H$_2$OCH$_3$), 77.39 (t, 14-C), 77.18 (t, 12-C), 74.15 (t, 11-C), 64.51 (s, 1´-C), 56.05 (p, -O*C*H$_3$), 55.59 (p, 2C, -O*C*H$_3$), 45.50 (t, -*C*H(CH$_2$CH$_2$CH$_3$)$_2$), 36.45 (t, 13-C), 34.84 (s, -CH(*C*H$_2$CH$_2$CH$_3$)$_2$), 34.82 (s, -CH(*C*H$_2$CH$_2$CH$_3$)$_2$), 20.85 (s, -CH(CH$_2$*C*H$_2$CH$_3$)$_2$), 20.84 (s, -CH(CH$_2$*C*H$_2$CH$_3$)$_2$), 14.17 (p, 2C, -CH(CH$_2$CH$_2$*C*H$_3$)$_2$), 11.30 (p, 2´-C) ppm; **HRMS-ESI** ($C_{22}H_{40}O_8$): calc. for $[M+Na]^+$: 455.26154, found: 455.26067.

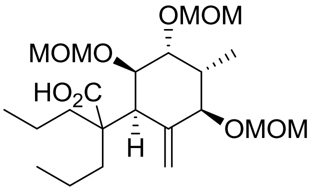


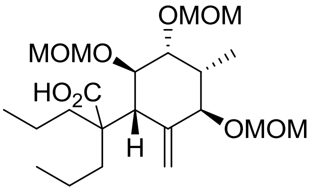


**50a** **50b**

**Carboxylic acids 50a and 50b** were prepared from ester **49** according to the *general procedure for the rearrangement of **29, 30, 45, 47, 49, 52, 54*** (95% yield, de = 72%).

**50a** *(major diastereomer):* **$R_f$** = 0.37 (petroleum ether: EtOAc = 1:1); **$^1$H NMR** (600 MHz, $CDCl_3$) δ = 9.77 (bs, 1H, $CO_2$*H*), 5.22 (s, 1H, 1´-$H_a$), 4.93 (s, 1H, 1´-$H_b$), 4.67 – 4.60 (m, 4H, -OC*H*$_2$O$CH_3$), 4.58 (d, *J* = 6.6 Hz, 1H, -OC*H*$_2$O$CH_3$), 4.50 (d, *J* = 6.9 Hz, 1H, -OC*H*$_2$O$CH_3$), 4.16 (d, *J* = 9.1 Hz, 1H, 14-H), 3.92 – 3.89 (m, 1H, 11-H), 3.74 (dd, *J* = 8.3 Hz, 1H, 12-H), 3.38 (s, 3H, -OC*H*$_3$), 3.35 (s, 3H, -OC*H*$_3$), 3.33 (s, 3H, -OC*H*$_3$), 2.80 (d, *J* = 2.5 Hz, 1H, 9-H), 2.04 – 1.98 (m, 1H, 13-H), 1.81 – 1.62 (m, 3H, alkyl), 1.81 – 1.62 (m, 3H, alkyl), 1.53 – 1.47 (m, 1H, alkyl), 1.36 – 1.09 (m, 4H, alkyl), 1.04 (d, *J* = 6.9 Hz, 3H, 2´-C), 0.90 (t, *J* = 7.2 Hz, 3H, -C*H*$_3$), 0.86 (t, *J* = 7.2, 3H, -C*H*$_3$) ppm; **$^{13}$C NMR** (151 MHz, $CDCl_3$) δ = 182.14 (q, -*C*$O_2$H), 143.95 (q, 8-C), 113.86 (s, 1´-C), 96.77 (s, -OC*H*$_2$O$CH_3$), 96.37 (s, -OC*H*$_2$O$CH_3$), 95.47 (s, -OC*H*$_2$O$CH_3$), 79.35 (t, 12-C), 78.65 (t, 14-C), 74.61 (t, 11-C), 56.19 (p, -O*C*$H_3$), 56.06 (p, 2C, -O*C*$H_3$), 53.30 (t, 9-C), 49.98 (q, cyclohexyl), 38.71 (t, 13-C), 36.43 (s, alkyl), 34.99 (s, alkyl), 17.69 (s, alkyl), 17.23 (s, alkyl), 14.89 (p, 2C, -*C*$H_3$), 14.84 (p, 2´-C) ppm; **HRMS-ESI** ($C_{22}H_{40}O_8$): calc. for $[M+H]^+$: 433.27959, found: 433.27953.

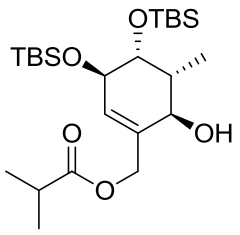


**S12**

**Isobutyrate S12.** To a solution of alcohol **41** (56 mg, 0.14 mmol) in $CH_2Cl_2$ (5 ml) were added EDC·HCl (35 mg, 0.18 mmol), isobutyric acid (13 µl, 0.15 mmol) and DMAP (4.0 mg, 0.03 mmol). The reaction mixture was stirred for 20 h at room temperature and then quenched with 1 M HCl (10 ml). The two layers were separated and the organic layer was washed with aqueous saturated $NaHCO_3$-solution (10 ml) and $H_2O$ (2 × 10 ml). The combined aqueous layers were extracted with $CH_2Cl_2$ (20 ml) and the combined organic layers were dried ($MgSO_4$) and evaporated under reduced pressure. Purification by flash chromatography on silica (petroleum ether/EtOAc 10:1) gave ester **S12** (53 mg, 81%) as a colourless oil.

**$R_f$** = 0.50 (petroleum ether:EtOAc = 9:1); $[\alpha]_D^{20}$ = –8.1 (*c* = 1.06 in $CHCl_3$); **$^1$H NMR** (400 MHz, $CDCl_3$) δ = 5.63 (d, *J* = 4.1 Hz, 1H, 9-H), 4.90 (d, *J* = 12.4 Hz, 1H, 1´-$H_a$), 4.43 (d, *J* = 12.4 Hz, 1H, 1´-$H_b$), 3.91 – 3.87 (m, 1H, 11-H), 3.85 (d, *J* = 7.7 Hz, 1H, 14-H), 3.66 – 3.62 (m, 1H, 12-H), 2.61 – 2.47 (m, 1H, -C*H*($CH_3$)$_2$), 2.27 (bs, 1H, -OH), 2.06 – 1.93 (m, 1H,

13-H), 1.14 (d, $J$ = 7.0 Hz, 6H, -CH(C$H_3$)$_2$), 1.05 (d, $J$ = 6.9 Hz, 3H, -CH(C$H_3$)$_2$), 0.85 (s, 9H, -SiC(C$H_3$)$_3$)), 0.83 (s, 9H, -SiC(C$H_3$)$_3$), 0.07 – -0.01 (m, 12H, -Si(C$H_3$)$_2$) ppm; **$^{13}$C NMR** (101 MHz, CDCl$_3$) δ = 177.59 (q, *C*=O), 137.82 (q, 8-C), 128.21 (t, 9-C), 75.49 (t, 12-C), 70.34 (t, 14-C), 69.39 (t, 11-C), 65.30 (s, 1´-C), 37.80 (t, 13-C), 34.30 (t, cyclohexyl), 26.07 (p, 3C, SiC(*C*H$_3$)$_3$), 26.00 (p, 3C, SiC(*C*H$_3$)$_3$), 19.22 (p, -CH(*C*H$_3$)$_2$), 19.15 (p, -CH(*C*H$_3$)$_2$), 18.28 (q, Si*C*(CH$_3$)$_3$), 18.21 (q, Si*C*(CH$_3$)$_3$), 14.34 (p, 2´-C), -4.02 (p, -Si(*C*H$_3$)$_2$), -4.17 (p, -Si(*C*H$_3$)$_2$), -4.26 (p, -Si(*C*H$_3$)$_2$), -4.59 (p, -Si(*C*H$_3$)$_2$) ppm; **HRMS-ESI** ($C_{24}H_{48}O_5Si_2$): calc. for $[M+Na]^+$: 495.29325, found: 495.29290.

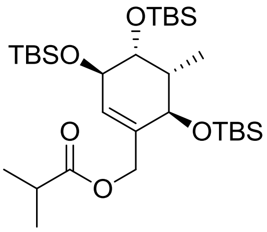


**52**

**Isobutyrate 52**. To a solution of alcohol **S12** (42 mg, 89 μmol) in DMF (3 ml) was added imidazole (75.6 mg, 1.11 mmol), TBSCl (133 mg, 888 μmol) and DMAP (5.0 mg, 44 μmol). The reaction mixture was stirred for 40 h at room temperature. $CH_2Cl_2$ (10 ml) and brine (10 ml) were added and the resulting two layers were separated. The aqueous layer was extracted with $CH_2Cl_2$ (2 × 10 ml) and the combined organic layes were dried ($MgSO_4$) and evaporated under reduced pressure. Purification by flash chromatography on silica (petroleum ether/EtOAc 20:1) gave ester **52** (50 mg, 96%) as a yellowish oil.

**R$_f$** = 0.51 (petroleum ether:EtOAc = 20:1); $[\boldsymbol{\alpha}]_{\mathbf{D}}^{\mathbf{20}}$ = –23.1 (*c* = 0.90 in CHCl$_3$); **$^1$H NMR** (400 MHz, CDCl$_3$) δ = 5.58 – 5.55 (m, 1H, 9-H), 4.54 – 4.46 (m, 2H, 1´-H$_a$, 1´-H$_b$), 4.00 (bd, $J$ = 7.1, 1H, 11-H), 3.92 (d, $J$ = 2.9, 1H, 14-H), 3.87 (dd, $J$ = 7.1, 3.8, 1H, 12-H), 2.64 – 2.46 (m, 1H, -C*H*(CH$_3$)$_2$), 2.05 – 1.90 (m, 1H, 13-H), 1.16 (d, $J$ = 7.0, 6H, -CH(C$H_3$)$_2$), 0.92 – 0.86 (m, 30H, -SiC(C$H_3$)$_3$), 2´-H), 0.09 (s, 3H, -Si(C$H_3$)$_2$), 0.07 (s, 3H, -Si(C$H_3$)$_2$), 0.06 (s, 12H, -Si(C$H_3$)$_2$) ppm; **$^{13}$C NMR** (101 MHz, CDCl$_3$) δ = 176.73 (q, *C*=O), 134.21 (q, 8-C), 129.61 (t, 9-C), 73.01 (t, 12-C), 71.62 (t, 14-C), 70.47 (t, 11-C), 64.95 (s, 1´-C), 41.79 (t, -*C*H(CH$_3$)$_2$), 34.33 (t, 13-C), 26.31 (p, 3C, SiC(*C*H$_3$)$_3$), 26.27 (p, 3C, SiC(*C*H$_3$)$_3$), 26.08 (p, 3C, SiC(*C*H$_3$)$_3$), 19.25 (p, -CH(*C*H$_3$)$_2$), 19.21 (p, -CH(*C*H$_3$)$_2$), 18.51 (q, Si*C*(CH$_3$)$_3$), 18.29 (q, Si*C*(CH$_3$)$_3$), 18.24 (q, Si*C*(CH$_3$)$_3$), 11.57 (p, 2´-C), -3.72 (p, -Si(*C*H$_3$)$_2$), -3.80

(p, -Si($C$H$_3$)$_2$), -4.10 (p, -Si($C$H$_3$)$_2$), -4.30 (p, -Si($C$H$_3$)$_2$), -4.35 (p, -Si($C$H$_3$)$_2$), -4.75 (p, -Si($C$H$_3$)$_2$) ppm; **HRMS-ESI** ($C_{30}H_{62}O_5Si_3$): calc. for $[M+Na]^+$: 609.37973, found: 609.37903.

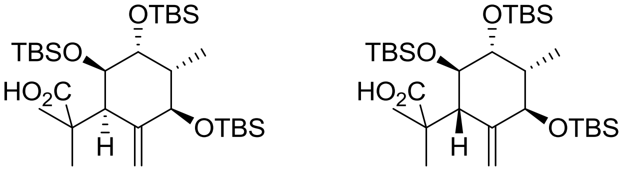


**53a** **53b**

**Carboxylic acids 53a and 53b** were prepared from ester **52** according to the *general procedure for the rearrangement of **29, 30, 45, 47, 49, 52, 54*** (quant., de = 94%).

**53a** (*major diastereomer*): $\mathbf{R_f}$ = 0.33 (petroleum ether:EtOAc = 15:1); $[\alpha]_D^{20}$ = +29.5 (*c* = 1.00 in $CHCl_3$); **$^1$H NMR** (600 MHz, $CDCl_3$) δ = 5.28 – 5.27 (m, 1H, 1´-H$_a$), 4.76 (s, 1H, 1´-H$_b$), 4.18 (d, *J* = 11.0 Hz, 1H, 14-H), 3.77 (d, *J* = 2.4 Hz, 1H, 11-H), 3.63 (bs, 1H, 12-H), 2.92 (bs, 1H, 9-H), 1.93 (dqd, *J* = 11.0, 7.0, 2.5 Hz, 1H, 13-H), 1.28 (s, 3H, -C$H_3$), 1.25 (s, 3H, -C$H_3$), 0.94 (d, *J* = 7.0 Hz, 3H, 2´-H), 0.92 (s, 9H, -SiC(C$H_3$)$_3$), 0.91 (s, 9H, -SiC(C$H_3$)$_3$), 0.81 (s, 9H, -SiC(C$H_3$)$_3$), 0.06 (s, 3H, -Si(C$H_3$)$_2$), 0.06 (s, 3H, -Si(C$H_3$)$_2$), 0.04 (s, 3H, -Si(C$H_3$)$_2$), 0.03 (s, 3H, -Si(C$H_3$)$_2$), 0.01 (s, 3H, -Si(C$H_3$)$_2$), 0.00 (s, 3H, -Si(C$H_3$)$_2$) ppm; **$^{13}$C NMR** (151 MHz, $CDCl_3$) δ = 183.99 (q, -$C$(O)OH), 147.34 (q, 8-C), 113.06 (s, 1´-C), 78.07 (t, 12-C), 73.26 (t, 11-C), 71.75 (t, 14-C), 59.43 (t, 9-C), 46.06 (q, -$C$(CH$_3$)$_2$(CO$_2$H)), 41.14 (t, 13-C), 26.57 (p, 3C, SiC($C$H$_3$)$_3$), 26.39 (p, -$C$H$_3$), 26.24 (p, 3C, SiC($C$H$_3$)$_3$), 25.90 (p, 3C, SiC($C$H$_3$)$_3$), 23.45 (p, -$C$H$_3$), 18.58 (q, Si$C$(CH$_3$)$_3$), 18.53 (q, Si$C$(CH$_3$)$_3$), 18.05 (q, Si$C$(CH$_3$)$_3$), 15.27 (p, 2´-C), -3.21 (p, -Si($C$H$_3$)$_2$), -3.94 (p, -Si($C$H$_3$)$_2$), -4.28 (p, -Si($C$H$_3$)$_2$), -4.36 (p, -Si($C$H$_3$)$_2$), -4.50 (p, -Si($C$H$_3$)$_2$), -4.65 (p, -Si($C$H$_3$)$_2$) ppm; **HRMS-ESI** ($C_{30}H_{62}O_5Si_3$): calc. for $[M+Na]^+$: 609.37944, found: 609.37973.

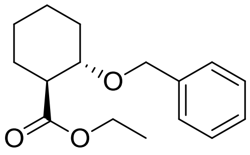

**S13**

**Ethyl (1*S*,2*S*)-2-(benzyloxy)cyclohexanecarboxylate S13.** To a stirred solution of ethyl (1*S*,2*S*)-2-(hydroxy)cyclohexanecarboxylate (0.86 g, 5 mmol) and benzyl trichloro-acetimidate (1.12 ml) in cyclohexane (7 ml) and $CH_2Cl_2$ (3.5 ml) at room temperature was added dropwise TfOH (71 µl). After 1 h, the mixture was filtered and the filter cake was washed with petroleum ether/EtOAc 9:1. The filtrate was washed with saturated aqueous $NaHCO_3$-solution (20 ml) and water (20 ml), filtered and evaporated in vacuo to afford 1.7 g of a yellow oil which was purified by flash chromatography on silica gel (petroleum ether/EtOAc 96:4) affording a pale yellow oil (1.16 g, 88%).

**$R_f$** = 0.23 (petroleum ether:EtOAc = 19:1); **$^1$H NMR** (400 mHz, $CDCl_3$) δ = 7.34 – 7.24 (m, 5H), 4.61 (d, *J* = 11.5 Hz, 1H), 4.48 (d, *J* = 11.5 Hz, 1H), 4.19 – 4.09 (m, 2H), 3.63 (td, *J* = 9.9, 4.5 Hz, 1H), 2.46 – 2.39 (m, 1H), 2.18 – 2.11 (m, 1H), 1.98 – 1.89 (m, 1H), 1.82 – 1.75 (m, 1H), 1.72 – 1.65 (m, 1H), 1.56 – 1.43 (m, 1H), 1.31 – 1.16 (m, 3H), 1.24 (t, *J* = 7.2 Hz, 3H) ppm; **HRMS** ($C_{16}H_{22}O_3$): calc. for $[M+H]^+$: 263.1642, found: 263.1643.

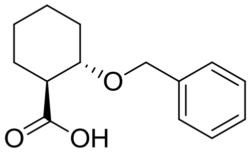

**S14**

**(1*S*,2*S*)-2-(Benzyloxy)cyclohexanecarboxylic acid S14.** To a stirred solution of **S13** (1.16 g, 4.42 mmol) in MeOH (10 ml) at room temperature was added LiOH (2 M, 6.6 ml). After 20 h, the mixture was extracted with EtOAc (50 ml). The organic layer was acidified to pH < 2 and extracted with EtOAc (3 × 50 ml). The combined organic layers were dried ($MgSO_4$) and concentrated in vacuo to afford 1.06 g of yellow oil which was purified by flash chromatography on silica gel (petroleum ether/$Et_2O$ 9:1) to give compound **S14** (0.42 g, 41%) as a pale yellow oil.

**R*f*** = 0.04 (petroleum ether:$Et_2O$ = 9:1); $[\alpha]_D^{20}$ = +80.2 (*c* = 0.65 in $CHCl_3$); **$^1$H NMR** (400 mHz, $CDCl_3$) δ = 7.33 – 7.22 (m, 5H), 4.65 (d, *J* = 11.5 Hz, 1H), 4.52 (d, *J* = 11.5 Hz, 1H), 3.66 – 3.59 (m, 1H), 2.50 – 2.43 (m, 1H), 2.22 – 2.15 (m, 1H), 2.09 – 2.01 (m, 1H), 1.84 – 1.77 (m, 1H), 1.76 – 1.67 (m, 1H), 1.55 – 1.44 (m, 1H), 1.32 – 1.21 (m, 3H) ppm; **$^{13}$C NMR** (100 MHz, $CDCl_3$) δ = 180.0, 138.5, 128.6 (2C), 128.0 (2C), 127.8, 78.5, 71.2, 49.9, 30.8, 28.6, 24.9, 24.2 ppm; **HRMS** ($C_{14}H_{18}O_3$): calc. for $[M]^+$: 234.1250, found: 234.1253.

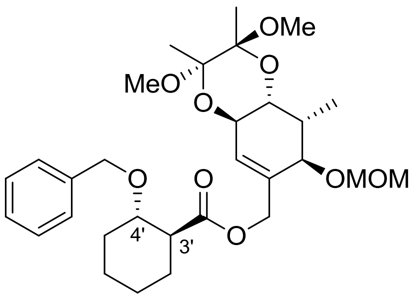


**S15**

**β-Alkoxy ester S15.** To a solution of alcohol **19** (200 mg, 602 μmol) in $CH_2Cl_2$ (20 ml) were added carboxylic acid **S14** (240 mg, 1.02 mmol), DCC (186 mg, 903 μmol) and DMAP (29 mg, 0.24 mmol). The reaction mixture was stirred for 5 h at room temperature and then evaporated under reduced pressure. Purification by flash chromatography on silica (petroleum ether/EtOAc 5:1) gave ester **S15** (318 mg, 96%) as a colourless oil.

**R*f*** = 0.21 (petroleum ether:EtOAc = 5:1); $[\alpha]_D^{20}$ = + 82.5 (*c* = 2.0 in $CH_2Cl_2$); **$^1$H NMR** (400 MHz, $CDCl_3$) δ = 7.30 – 7.14 (m, 5H, Ar-H), 5.74 (s, 1H, 9-H), 4.68 – 4.42 (m, 6H, -OC$H_2$Ar, -OC$H_2$O$CH_3$, 1´-$H_a$, 1´-$H_b$), 4.18 (d, *J* = 9.3 Hz, 1H, 11-H), 3.93 (dd, *J* = 9.3, 4.0 Hz, 1H, 12-H), 3.77 (s, 1H, 14-H), 3.60 (td, *J* = 9.8, 4.4 Hz, 1H, 4´-H), 3.32 (s, 3H, -$OCH_2OCH_3$), 3.18 (s, 6H, -O$CH_3$), 2.48 – 2.38 (m, 1H, 3´-H), 2.26 – 2.17 (m, 1H, 13-H), 2.13 – 2.05 (m, 1H, cyclohexyl), 2.00 – 1.96 (m, 1H, cyclohexyl), 1.94 – 1.86 (m, 1H, cyclohexyl), 1.78 – 1.70 (m, 1H, cyclohexyl), 1.70 – 1.60 (m, 1H, cyclohexyl), 1.52 – 1.40 (m, 1H, cyclohexyl), 1.26 (s, 3H, -$CH_3$), 1.25 (s, 3H, -$CH_3$), 1.24 – 1.17 (m, 2H, cyclohexyl), 0.89 (d, *J* = 7.3 Hz, 3H, 2´-H) ppm; **$^{13}$C NMR** (101 MHz, $CDCl_3$) δ = 174.58 (q, *C*=O), 138.83, 133.40, 128.19 (2C), 128.05, 127.47 (2C), 127.35 (Ar-C, 8-C, 9-C), 100.37 (q, BDA), 100.18 (q, BDA), 96.64 (s, -O$CH_2OCH_3$), 78.47 (t, 4´-C), 77.58 (t, 14-C), 70.85 (s, -O$CH_2$R), 67.42 (t, 12-C), 64.66 (-O$CH_2$R, 11-C), 55.78 (p, -$OCH_2OCH_3$), 50.35 (t, 3´-C), 47.73 (p, -$CH_3$), 47.56 (p, -$CH_3$), 35.53 (t, 13-C), 30.62 (s, cyclohexyl), 28.82 (s, cyclohexyl), 24.7

(s, cyclohexyl), 24.02 (s, cyclohexyl), 17.85 (p, 2C, -*C*$H_3$), 10.67 (p, 2´-C) ppm; **HRMS-ESI** ($C_{30}H_{44}O_9$): calc. for $[M+NH_4]^+$: 566.33236, found: 566.33229.

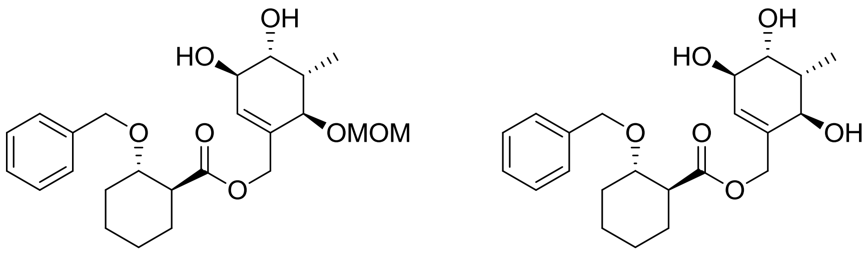

**S16** **S17**

**Diol S16 and triol S17.** A solution of **S15** (220 mg, 401 μmol) in a mixture of $CH_2Cl_2$ (1.2 ml), TFA (1.2 ml) and $H_2O$ (0.2 ml) was stirred for 10 min at room temperature. $H_2O$ (5 ml) and $CH_2Cl_2$ (5 ml) were added and the two layers were separated. The aqueous layer was extracted with $CH_2Cl_2$ (3 × 10 ml) and the combined organic layers were washed with aqueous saturated $NaHCO_3$-solution (20 ml) and brine (10 ml), dried ($MgSO_4$) and evaporated under reduced pressure. Purification by flash chromatography on silica (petroleum ether/EtOAc 1:1 to EtOAc) gave diol **S16** (109 mg, 63%) and triol **S17** (23 mg, 14%).

**Diol S16:** $\mathbf{R}_f$ = 0.50 (EtOAc); **$^1$H NMR** (500 MHz, $CDCl_3$) δ = 7.37 – 7.22 (m, 5H, Ar-H), 5.77 (s, 1H, 9-H), 4.74 (d, *J* = 7.0 Hz, 1H, -OC*H*$_2$O$CH_3$), 4.69 – 4.57 (m, 4H, -OC*H*$_2$Ar, -OC*H*$_2$O$CH_3$, 1´-$H_a$), 4.50 (d, *J* = 11.6 Hz, 1H, 1´-$H_b$), 4.00 (d, *J* = 8.1 Hz, 1H, 11-H), 3.89 (dd, *J* = 8.1, 4.1 Hz, 1H, 12-H), 3.86 (d, *J* = 1.1 Hz, 1H, 14-H), 3.67 (td, *J* = 9.8, 4.2 Hz, 1H, 4´-H), 3.39 (s, 3H, -OC*H*$_3$), 2.66 (bs, 2H, -OH), 2.54 – 2.47 (m, 1H, 3´-H), 2.34 – 2.27 (m, 1H, 13-H), 2.21 – 2.14 (m, 1H, cyclohexyl), 2.01 – 1.94 (m, 1H, cyclohexyl), 1.85 – 1.78 (m, 1H, cyclohexyl), 1.75 – 1.68 (m, 1H, cyclohexyl), 1.56 – 1.45 (m, 1H, cyclohexyl), 1.37 – 1.22 (m, 3H, cyclohexyl), 0.90 (d, *J* = 7.3 Hz, 3H, 2´-H) ppm; **$^{13}$C NMR** (126 MHz, $CDCl_3$) δ = 174.85 (q, *C*=O), 139.00, 133.17, 129.77, 128.44 (2C), 127.72 (2C), 127.63 (Ar-C, 8-C, 9-C), 96.46 (s, -O*C*$H_2$O$CH_3$), 78.82 (t, 4´-C), 76.83 (t, 14-C), 72.20 (t, 12-C), 71.08 (s, -O*C*$H_2$Ar), 70.04 (t, 11-C), 64.67 (s, 1´-C), 56.17 (p, -O*C*$H_3$), 50.56 (t, 3´-C), 37.66 (t, 13-C), 30.79 (s, cyclohexyl), 29.07 (s, cyclohexyl), 24.91 (s, cyclohexyl), 24.22 (s, cyclohexyl), 10.39 (p, 2´-C) ppm; **HRMS-ESI** ($C_{24}H_{34}O_7$): calc. for 452.26428 $[M+NH_4]^+$, found 452.26428.

**Triol S17:** **R$_f$** = 0.23 (EtOAc); $[\alpha]_D^{20}$ = +30.7 (*c* = 1.0 in $CHCl_3$); **$^1$H NMR** (500 MHz, $CDCl_3$) δ = 7.36 – 7.25 (m, 5H, Ar-H), 5.70 (s, 1H, 9-H), 4.70 – 4.58 (m, 3H, -OC*H*$_2$Ar, 1´-H$_a$, 1´-H$_b$), 4.49 (d, *J* = 11.6 Hz, 1H, -OC*H*$_2$Ar), 3.99 (bd, *J* = 7.7 Hz, 1H, 11-H), 3.92 (dd, *J* = 7.7, 3.9 Hz, 1H, 12-H), 3.80 (d, *J* = 2.4 Hz, 1H, 14-H), 3.70 – 3.62 (m, 1H, 4´-H), 3.54 (bs, 3H, -OH), 2.54 – 2.45 (m, 1H, 3´-H), 2.20 – 2.14 (m, 1H, cyclohexyl), 2.12 – 2.06 (m, 1H, 13-H), 1.99 – 1.94 (m, 1H, cyclohexyl), 1.84 – 1.78 (m, 1H, cyclohexyl), 1.74 – 1.68 (m, 1H, cyclohexyl), 1.55 – 1.46 (m, 1H, cyclohexyl), 1.34 – 1.21 (m, 3H, cyclohexyl), 0.87 (d, *J* = 7.3 Hz, 3H, 2´-H) ppm; **$^{13}$C NMR** (126 MHz, $CDCl_3$) δ = 175.24 (q, *C*=O), 138.81, 134.92 (2 x q, Ar-C, 8-C), 129.18 (t, 9-C), 128.51 (t, 2C, Ar-C), 127.88 (t, 2C, Ar-C), 127.77 (t, Ar-C), 79.01 (t, 4´-C), 71.74 (t, 12-C), 71.29 (t, 14-C), 71.05 (s, -O*C*H$_2$Ar), 69.76 (t, 11-C), 65.51 (s, 1´-C), 50.62 (t, 3´-C), 40.77 (t, 13-H), 30.79 (s, cyclohexyl), 29.03 (s, cyclohexyl), 24.88 (s, cyclohexyl), 24.24 (s, cyclohexyl), 10.74 (p, 2´-C) ppm; **HRMS-ESI** ($C_{22}H_{30}O_6$): calc. for $[M+Na]^+$: 413.19346, found: 413.19339.

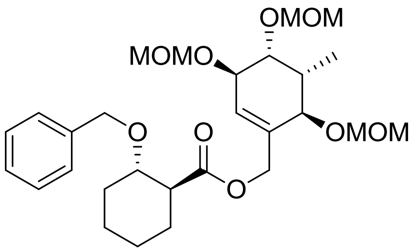


**56**

**MOM ether 56.** To a solution of diol **S16** (80 mg, 0.18 mmol) in $CH_2Cl_2$ (7 ml) were added DIPEA (164 µl, 921 µmol) and MOMCl (70 µl, 0.92 mmol). The reaction mixture was heated for 18 h under reflux and then quenched with $H_2O$ (10 ml). The resulting two layers were separated and the aqueous layer was extracted with $CH_2Cl_2$ (3 × 10 ml). The combined organic layers were washed with brine (20 ml), dried ($MgSO_4$) and evaporated under reduced pressure. Purification by flash chromatography on silica (petroleum ether/EtOAc 4:1) gave MOM ether **56** (89 mg, 93%) as a colourless oil.

**R$_f$** = 0.23 (petroleum ether:EtOAc = 4:1); $[\alpha]_D^{20}$ = +26.8 (*c* = 1.00 in $CH_2Cl_2$); **$^1$H NMR** (500 MHz, $CDCl_3$) δ = 7.35 – 7.23 (m, 5H, Ar-H), 5.85 – 5.83 (m, 1H, 9-H), 4.84 – 4.48 (m, 10H, -OC*H*$_2$Ar, -OC*H*$_2$OCH$_3$, 1´-H$_a$, 1´-H$_b$), 4.09 (bd, *J* = 7.7 Hz, 1H, 11-H), 3.97 (dd, *J* = 7.7, 4.0 Hz, 1H, 12-H), 3.85 (d, *J* = 2.9 Hz, 1H, 14-H), 3.66 (td, *J* = 9.9, 4.5 Hz, 1H, 4´-H), 3.42 (s, 3H, -OC*H*$_3$), 3.41 (s, 3H, -OC*H*$_3$), 3.40 (s, 3H, -OC*H*$_3$), 2.50 (ddd, *J* = 16.3, 9.9,

4.1 Hz, 1H, 3´-H), 2.46 – 2.40 (m, 1H, 13-H), 2.21 – 2.12 (m, 1H, cyclohexyl), 2.00 – 1.94 (m, 1H, cyclohexyl), 1.84 – 1.78 (m, 1H, cyclohexyl), 1.74 – 1.68 (m, 1H, cyclohexyl), 1.57 – 1.47 (m, 1H, cyclohexyl), 1.38 – 1.20 (m, 3H, cyclohexyl), 0.95 (d, *J* = 7.3 Hz, 3H, 2´-H) ppm; **$^{13}$C NMR** (126 MHz, $CDCl_3$) δ = 174.77 (q, *C*=O), 139.02, 133.79, 128.66, 128.41 (2C), 127.72 (2C), 127.58 (Ar-C, 8-C, 9-C), 97.15 (s, -O*C*$H_2OCH_3$), 96.84 (s, -O*C*$H_2OCH_3$), 96.59 (s, -O*C*$H_2OCH_3$), 78.73 (t, 4´-C), 77.40 (t, 14-C), 77.20 (t, 12-C), 74.14 (t, 11-C), 71.19 (s, -O*C*$H_2$R), 64.85 (s, -O*C*$H_2$R), 56.06 (p, -O*C*$H_3$), 55.61 (p, 2C, -O*C*$H_3$), 50.50 (t, 3´-C), 36.49 (t, 13-H), 30.83 (s, cyclohexyl), 29.05 (s, cyclohexyl), 24.92 (s, cyclohexyl), 24.22 (s, cyclohexyl), 11.37 (p, 2´-C) ppm; **HRMS-ESI** ($C_{28}H_{42}O_9$): calc. for $[M+NH_4]^+$: 540.31671, found: 540.31669.

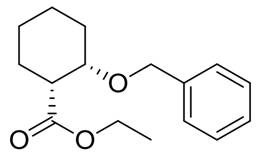

**S18**

**Ethyl (1*R*,2*S*)-2-(benzyloxy)cyclohexanecarboxylate S18.** To a stirred solution of ethyl (1*R*,2*S*)-2-(hydroxy)cyclohexanecarboxylate (1.10 g, 6.4 mmol) and benzyl trichloro-acetimidate (1.42 ml) in cyclohexane (8 ml) and $CH_2Cl_2$ (4 ml) at 0 °C was added dropwise TfOH (90 µl). After 30 min, the mixture was allowed to warm to room temperature and stirred for 1 h. The reaction mixture was then washed with saturated aqueous $NaHCO_3$-solution (20 ml) and water (20 ml), filtered and evaporated in vacuo to afford 2.8 g of a yellow solid which was purified by flash chromatography on silica gel (petroleum ether/EtOAc 96:4) affording a clear oil (1.46 g, 88%).

**R$_f$** = 0.25 (petroleum ether:EtOAc = 19:1); $[\boldsymbol{\alpha}]_{\mathbf{D}}^{\mathbf{20}}$ = +43.0 (*c* = 1.05 in $CHCl_3$); **$^1$H NMR** (400 mHz, $CDCl_3$) δ = 7.35 – 7.19 (m, 5H), 4.59 (d, *J* = 12.0 Hz, 1H), 4.42 (d, *J* = 12.0 Hz, 1H), 4.17 – 4.03 (m, 3H), 2.47 – 2.42 (m, 1H), 2.11 – 2.04 (m, 1H), 1.97 – 1.86 (m, 1H), 1.82 – 1.72 (m, 2H), 1.68 – 1.56 (m, 1H), 1.47 – 1.22 (m, 3H), 1.20 (t, *J* = 7.1 Hz, 3H) ppm; **$^{13}$C NMR** (100 MHz, $CDCl_3$) δ = 173.9, 139.2, 128.4 (2C), 127.5 (2C), 127.5, 74.8, 70.7, 60.3, 47.2, 28.6, 24.8, 23.0, 20.3, 14.4 ppm; **HRMS** ($C_{16}H_{22}O_3$): calc. for $[M]^+$: 262.1563, found: 262.1561.

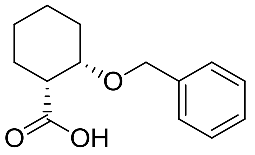


**S19**

**(1*R*,2*S*)-2-(Benzyloxy)cyclohexanecarboxylic acid S19.** To a stirred solution of **S18** (1.31 g, 5 mmol) in EtOH (25 ml) at room temperature was added LiOH (1.5 M, 6.7 ml, 10 mmol). The mixture was stirred for 20 h. After evaporation in vacuo the remaining aqueous solution was acidified to pH = 7 and extracted with $CH_2Cl_2$ (3 × 20 ml). The combined organic layers were dried over magnesium sulfate, filtered and evaporated in vacuo to afford 0.93 g of a clear oil which was purified by flash chromatography on silica gel (petroleum ether/EtOAc 9:1) to give compound **S19** (0.51 g, 44%). The previous aqueous layer was acidified to pH 2 and extracted with $CH_2Cl_2$ (3 × 20 ml). The combined organic layers were dried ($MgSO_4$), filtered and evaporated in vacuo to afford the desired product **S19** (0.30 g, 26%; total: 69%).

$\mathbf{R}_f$ = 0.08 (petroleum ether:EtOAc = 9:1); $[\alpha]_D^{20}$ = +33.5 (*c* = 0.95 in $CH_3CN$); **$^1$H NMR** (400 mHz, $CDCl_3$) δ = 7.36 – 7.26 (m, 5H), 4.67 (d, *J* = 11.7 Hz, 1H), 4.52 (d, *J* = 11.7 Hz, 1H), 4.00 – 3.95 (m, 1H), 2.67 – 2.61 (m, 1H), 2.09 – 2.00 (m, 1H), 1.95 – 1.86 (m, 1H), 1.71 – 1.61 (m, 3H), 1.61 – 1.52 (m, 1H), 1.44 – 1.32 (m, 2H) ppm; **$^{13}$C NMR** (100 MHz, $CDCl_3$) δ = 178.2, 138.2, 128.6 (2C), 127.9, 127.8 (2C), 75.2, 70.9, 46.5, 28.5, 24.2, 23.8, 20.9 ppm; **HRMS** ($C_{14}H_{18}O_3$): calc. for $[M]^+$: 234.1250, found: 234.1247.

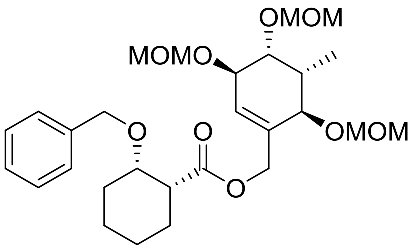


**57**

**β-Alkoxy ester 57.** To a solution of alcohol **40** (195 mg, 637 μmol) in $CH_2Cl_2$ (4 ml) were added a solution of carboxylic acid **S19** (179 mg, 764 μmol) in $CH_2Cl_2$ (1 ml), EDC·HCl (158 mg, 827 μmol) and DMAP (19 mg, 0.16 mmol). The reaction mixture was stirred for 21 h at room temperature. 1 M HCl (10 ml) was added and the resulting two layers were separated. The organic layer was washed with aqueous saturated $NaHCO_3$-solution (10 ml)

and $H_2O$ (2 × 10 ml). The combined aqueous layers were extracted with $CH_2Cl_2$ (20 ml) and the combined organic layers were dried ($MgSO_4$) and evaporated under reduced pressure. Purification by flash chromatography on silica (petroleum ether/EtOAc 3:1 to 2:1) gave **57** (317 mg, 95%) as a colourless oil.

**R*f*** = 0.37 (petroleum ether:EtOAc = 2:1); $[\alpha]_D^{20}$ = +26.7 (*c* = 1.0 in $CHCl_3$); **$^1$H NMR** (400 MHz, $CDCl_3$) δ = 7.30 – 7.16 (m, 5H, Ar-H), 5.77 – 5.74 (m, 1H, 9-H), 4.72 (d, *J* = 6.8 Hz, 1H, -OC*H*$_2$OCH$_3$), 4.70 (d, *J* = 6.7 Hz, 1H, -OC*H*$_2$OCH$_3$), 4.68 – 4.64 (m, 2H, 1´-H$_b$, -OC*H*$_2$OCH$_3$), 4.62 (d, *J* = 7.1 Hz, 1H, -OC*H*$_2$OCH$_3$), 4.59 – 4.48 (m, 4H, -OC*H*$_2$OCH$_3$, -OC*H*$_2$-Ar), 4.38 (d, *J* = 12.0 Hz, 1H, 1´-H$_a$), 4.03 – 3.98 (m, 2H, 11-H, 4´-H), 3.90 (dd, *J* = 7.8, 3.9 Hz, 1H, 12-H), 3.76 (d, *J* = 3.1 Hz, 1H, 14-H), 3.34 (d, *J* = 0.7 Hz, 3H, -OCH$_2$OC*H*$_3$), 3.33 (d, *J* = 0.7 Hz, 3H, -OCH$_2$OC*H*$_3$), 3.32 (d, *J* = 0.6 Hz, 3H, -OCH$_2$OC*H*$_3$), 2.46 (dt, *J* = 11.5, 3.5 Hz, 1H, 3´-H), 2.36 (ddq, *J* = 3.1, 3.9, 7.2, 1H, 13-H), 2.07 – 1.98 (m, 1H, cyclohexyl), 1.95 – 1.82 (m, 1H, cyclohexyl), 1.78 – 1.67 (m, 2H, cyclohexyl), 1.63 – 1.51 (m, 1H, cyclohexyl), 1.42 – 1.28 (m, 2H, cyclohexyl), 1.27 – 1.16 (m, 1H, cyclohexyl), 0.87 (d, *J* = 7.2 Hz, 3H, 2´-H) ppm; **$^{13}$C NMR** (126 MHz, $CDCl_3$) δ = 173.31 (q, *C*=O), 139.17, 133.80 (q, 2C, Ar-C, 8-C), 128.34 (t, 2C, Ar-C), 128.26 (t, 9-C), 127.39 (t, Ar-C), 127.32 (t, 2C, Ar-C), 97.13 (s, -*C*H$_2$-OMe), 96.82 (s, -*C*H$_2$-OMe), 96.54 (s, -*C*H$_2$-OMe), 77.55 (t, 14-C), 77.15 (t, 12-C), 74.98, 74.19 (t, 2C, 11-C, 4´-C), 70.55 (s, 1´-C), 64.68 (s, -OC*H*$_2$-Ar), 56.01 (p, -O*C*H$_3$), 55.57 (p, -O*C*H$_3$), 55.55 (p, -O*C*H$_3$), 47.22 (t, 3´-C), 36.47 (t, 13-C), 28.53 (s, cyclohexyl), 24.67 (s, cyclohexyl), 23.12 (s, cyclohexyl), 20.29 (s, cyclohexyl), 11.34 (p, 2´-C) ppm; **HRMS-ESI** ($C_{28}H_{42}O_9$): calc. for $[M+NH_4]^+$: 540.31671, found: 540.31615.

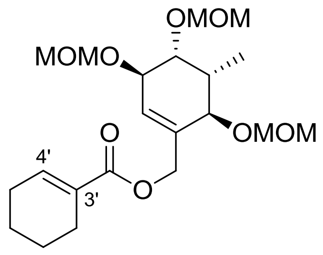


**58**

**Elimination product 58. R*f*** = 0.46 (petroleum ether:EtOAc = 2:1); $[\alpha]_D^{20}$ = +10.7 (*c* = 1.0 in $CHCl_3$); **$^1$H NMR** (500 MHz, $CDCl_3$) δ = 6.99 – 6.95 (m, 1H, 4´-H), 5.82 – 5.80 (m, 1H, 9-H), 4.78 (d, *J* = 6.8 Hz, 1H, -OC*H*$_2$OCH$_3$), 4.75 – 4.62 (m, 6H, -OC*H*$_2$OCH$_3$, 1´-H$_a$), 4.58

(d, $J$ = 13.2 Hz, 1H, 1´-$H_b$), 4.07 (d, $J$ = 7.8 Hz, 1H, 11-H), 3.95 (dd, $J$ = 7.8, 4.0 Hz, 1H, 12-H), 3.83 (d, $J$ = 3.0 Hz, 1H, 14-H), 3.38 (s, 3H, -$OCH_2OCH_3$), 3.37 (s, 3H, -$OCH_2OCH_3$), 3.36 (s, 3H, -$OCH_2OCH_3$), 2.41 (qdd, $J$ = 7.3, 4.0, 3.0 Hz, 1H, 13-H), 2.26 – 2.20 (m, 2H, cyclohexyl), 2.19 – 2.12 (m, 2H, cyclohexyl), 1.68 – 1.52 (m, 4H, cyclohexyl), 0.94 (d, $J$ = 7.3 Hz, 3H, 2´-H) ppm; $^{13}$**C NMR** (126 MHz, $CDCl_3$) δ = 167.24 (q, *C*=O), 140.31 (t, 4´-C), 134.01, 130.37 (2 x q, 3´-C, 8-C), 128.21 (t, 9-C), 97.12 (s, -$OCH_2OCH_3$), 96.95 (s, -$OCH_2OCH_3$), 96.62 (s, -$OCH_2OCH_3$), 77.40 (t, 14-C), 77.23 (t, 12-C), 74.38 (t, 11-C), 64.72 (s, 1´-C), 56.07 (p, -$OCH_2OCH_3$), 55.62 (p, -$OCH_2OCH_3$), 55.60 (p, -$OCH_2OCH_3$), 36.52 (t, 13-C), 26.02 (s, cyclohexyl), 24.34 (s, cyclohexyl), 22.26 (s, cyclohexyl), 21.63 (s, cyclohexyl), 11.40 (p, 2´-C) ppm; **HRMS-ESI** ($C_{21}H_{34}O_8$): calc. for $[M+H]^+$: 415.23264, found: 415.23242.

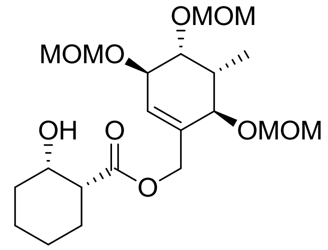


**59**

**β-Hydroxy ester 59.** To a solution of **57** (215 mg, 411 μmol) in a mixture of $CH_2Cl_2$ (9.5 ml) and $H_2O$ (0.5 ml) at room temperature was added DDQ (373 mg, 1.65 mmol). The reaction mixture was heated for 3.5 h under reflux. Aqueous saturated $NaHCO_3$-solution (10 ml) was added and the solution was filtered. The filter cake was washed with $CH_2Cl_2$ (2 × 20 ml) and the filtrate was washed with $H_2O$ (20 ml) and brine (20 ml), dried ($MgSO_4$) and evaporated under reduced pressure. Purification by flash chromatography on silica (petroleum ether/EtOAc 2:1) gave β-hydroxy ester **59** (160 mg, 90%) as a colourless oil.

$\mathbf{R}_f$ = 0.32 (petroleum ether:EtOAc = 1:1); $[\alpha]_D^{20}$ = + 4.6 ($c$ = 1.0 in $CHCl_3$); $^1$**H NMR** (400 MHz, $CDCl_3$) δ = 5.83 – 5.80 (m, 1H, 9-H), 4.78 (d, $J$ = 6.8 Hz, 1H, -$OCH_2OCH_3$), 4.75 – 4.67 (m, 5H, 4 x -$OCH_2OCH_3$, 1´-$H_a$), 4.63 (d, $J$ = 7.1 Hz, 1H, -$OCH_2OCH_3$), 4.52 (d, $J$ = 12.8 Hz, 1H, 1´-$H_b$), 4.19 – 4.14 (m, 1H, 4´-H), 4.07 (bd, $J$ = 7.6 Hz, 1H, 11-H), 3.92 (dd, $J$ = 7.6, 3.8 Hz, 1H, 12-H), 3.83 (d, $J$ = 3.2 Hz, 1H, 14-H), 3.38 (s, 6H, 2 x -$OCH_2OCH_3$), 3.36 (s, 3H, -$OCH_2OCH_3$), 3.10 (bs, 1H, OH), 2.51 – 2.44 (m, 1H, 3´-H), 2.38 (ddq, $J$ = 3.2, 3.8, 7.2 Hz, 1H, 13-H), 1.93 – 1.80 (m, 2H, cyclohexyl), 1.75 – 1.59 (m, 2H, cyclohexyl), 1.51 –

1.37 (m, 2H, cyclohexyl), 1.34 – 1.18 (m, 2H, cyclohexyl), 0.94 (d, *J* = 7.2 Hz, 3H, 2´-H) ppm; **$^{13}$C NMR** (126 MHz, $CDCl_3$) δ = 175.12 (q, *C*=O), 133.67 (q, 8-C), 129.13 (t, 9-C), 97.19 (s, -*C*$H_2$-OMe), 96.96 (s, -*C*$H_2$-OMe), 96.65 (s, -*C*$H_2$-OMe), 77.37 (2 x t, 14-C, 12-C), 74.14 (t, 11-C), 66.99 (t, 4´-C), 65.10 (s, 1´-C), 56.26 (p, -O*C*$H_3$), 55.68 (p, 2C, -O*C*$H_3$), 47.26 (t, 3´-C), 36.49 (t, 13-C), 32.23 (s, cyclohexyl), 25.02 (s, cyclohexyl), 23.81 (s, cyclohexyl), 20.20 (s, cyclohexyl), 11.60 (p, 2´-C) ppm; **HRMS-ESI** ($C_{21}H_{36}O_9$): calc. for $[M+NH_4]^+$: 450.26976, found: 450.26953.

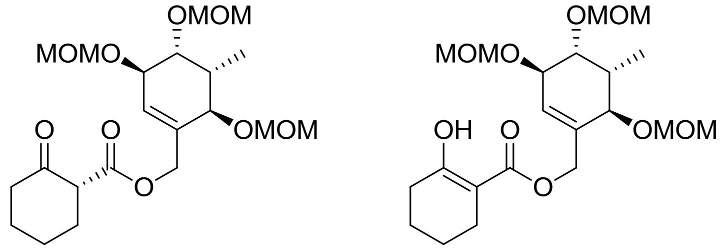


**60** **S20**

**β-Keto ester 60 and Enol ether S20**. To a solution of alcohol **59** (58 mg, 0.13 mmol) in $CH_2Cl_2$ (3 ml) was added Dess–Martin periodinane (85 mg, 0.20 mmol). The reaction mixture was stirred for 1 h at room temperature and then evaporated under reduced pressure. Purification by flash chromatography on silica (petroleum ether/EtOAc 2:1) gave a tautomeric mixture (ca. 10:1) of β-keto ester **60** and enol ether **S20** (40 mg, 69% combined yield) as a colourless oil.

**β-Keto ester 60: R$_f$** = 0.50 (Petroleum ether:EtOAc = 1:1) **$^1$H NMR** (400 MHz, $CDCl_3$) δ = 5.84 – 5.80 (m, 1H, 9-H), 4.80 – 4.58 (m, 8H, -OC*$H_2$*$OCH_3$, 1´-$H_a$, 1´-$H_b$), 4.09 – 4.05 (m, 1H, 11-H), 3.94 (dd, *J* = 7.8, 4.0 Hz, 1H, 12-H), 3.82 (d, *J* = 3.1 Hz, 1H, 14-H), 3.38 (s, 3H, -OC*$H_3$*), 3.37 (s, 3H, -OC*$H_3$*), 3.36 (s, 3H, -OC*$H_3$*), 2.48 – 2.37 (m, 2H, 13-H, 3´-H), 2.28 – 2.17 (m, 3H, cyclohexyl), 1.71 – 1.54 (m, 3H, cyclohexyl), 1.31 – 1.17 (m, 2H, cyclohexyl), 0.94 (d, *J* = 7.2 Hz, 3H, 2´-H) ppm; **$^{13}$C NMR** (101 MHz, $CDCl_3$) δ = 205.87 (q, *C*=O), 172.83 (q, -*C*(O)OR), 133.73 (q, 8-C), 128.30 (t, 9-C), 97.11 (s, -O*C*$H_2OCH_3$), 97.00 (s, -O*C*$H_2OCH_3$), 96.61 (s, -O*C*$H_2OCH_3$), 77.34 (t, 14-C), 77.19 (t, 12-C), 74.40 (t, 11-C), 64.53 (s, 1´-C), 56.09 (p, -O*C*$H_3$), 55.62 (p, -O*C*$H_3$), 55.61 (p, -O*C*$H_3$), 41.78 (t, 3´-C), 36.47 (t, 13-C), 29.33 (s, cyclohexyl), 22.58 (s, cyclohexyl), 22.54 (s, cyclohexyl), 22.07 (s, cyclohexyl), 11.40 (p, 2´-C) ppm; **HRMS-ESI** ($C_{21}H_{34}O_9$): calc. for $[M+NH_4]^+$: 448.25411, found: 448.25375.

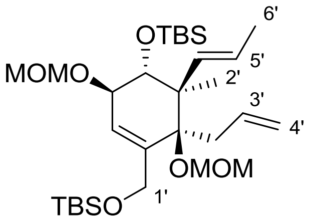


**S21**

***Bis*-MOM ether S21.** To a solution of diol **64** (53 mg, 0.11 mmol) in THF (2 ml) in a Schlenk-tube were added DIPEA (588 µl, 3.29 mmol), MOMCl (167 µl, 2.20 mmol) and NaI (82 mg, 0.55 mmol). The reaction mixture was heated for 20 h at 65 °C in a sealed tube. Additional DIPEA (588 µl, 3.29 mmol) and MOMCl (167 µl, 2.20 mmol) were added and the reaction mixture was stirred another 24 h at 65 °C in a sealed tube. Additional DIPEA (1.18 ml, 6.58 mmol) and MOMCl (167 µl, 2.20 mmol) were added and the reaction mixture was stirred another 24 h at 65 °C in a sealed tube. Although the starting material was not completely consumed, the reaction was quenched with $H_2O$ (10 ml) and the layers were separated. The aqueous layer was extracted with $CH_2Cl_2$ (3 × 10 ml) and the combined organic layers were dried ($MgSO_4$) and evaporated under reduced pressure. Purification by flash chromatography on silica (petroleum ether/EtOAc 20:1) gave *bis*-MOM ether **S21** (22 mg, 35%) as a colourless oil.

**$R_f$** = 0.32 (EtOAc:petroleum ether = 1:20); $[\alpha]_D^{20}$ = +27.9 (*c* = 1.00 in $CHCl_3$); **$^1$H NMR** (500 MHz, $CDCl_3$) δ = 5.96 – 5.94 (m, 1H, 9-H), 5.84 – 5.73 (m, 1H, 3´-H), 5.58 (dd, *J* = 15.7, 1.1 Hz, 1H, 17-H), 5.45 (dq, *J* = 15.7, 6.3 Hz, 1H, 5´-H), 5.16 – 5.11 (m, 1H, 4´-H), 5.10 – 5.06 (m, 1H, 4´-H), 5.05 (d, *J* = 7.4 Hz, 1H, C*H*$_2$-OMe), 4.72 (d, *J* = 6.8 Hz, 1H, C*H*$_2$-OMe), 4.67 (d, *J* = 6.8 Hz, 1H, C*H*$_2$-OMe), 4.43 (d, *J* = 7.4 Hz, 1H, C*H*$_2$-OMe), 4.32 – 4.27 (m, 1H, 1´-H), 4.22 – 4.17 (m, 1H, 1´-H), 3.92 (d, *J* = 7.7 Hz, 1H, 12-H), 3.82 – 3.78 (m, 1H, 11-H), 3.39 (s, 3H, -OC*H*$_3$), 3.32 (s, 3H, -OC*H*$_3$), 2.78 – 2.66 (m, 2H, 15-H), 1.70 (dd, *J* = 6.3, 1.1 Hz, 3H, 6´-H), 1.03 (s, 3H, 2´-H), 0.90 (s, 9H, -SiC(C*H*$_3$)$_3$), 0.80 (s, 9H, -SiC(C*H*$_3$)$_3$), 0.05 (s, 3H, -Si(C*H*$_3$)$_2$), 0.05 (s, 3H, -Si(C*H*$_3$)$_2$), -0.03 (s, 3H, -Si(C*H*$_3$)$_2$), -0.07 (s, 3H, -Si(C*H*$_3$)$_2$) ppm; **$^{13}$C NMR** (126 MHz, $CDCl_3$) δ = 140.57 (q, 8-C), 135.83 (t, 3´-C), 135.43 (t, 17-C), 126.50 (t, 5´-C), 123.16 (t, 9-C), 116.90 (s, 4´-C), 98.73 (s, -*C*H$_2$-OMe), 91.75 (s, -*C*H$_2$-OMe), 84.90 (q, 14-C), 81.14 (t, 11-C), 75.33 (t, 12-C), 62.02 (s, 1´-C), 55.79 (p, -O*C*H$_3$), 55.61 (p, -O*C*H$_3$), 52.95 (q, 13-C), 38.39 (s, 15-C), 26.22 (p, 3C, -SiC(*C*H$_3$)$_3$), 26.15 (p, 3C, -SiC(*C*H$_3$)$_3$), 19.05 (p, 6´-C), 18.57 (q, -Si*C*(CH$_3$)$_3$), 18.35 (q, -Si*C*(CH$_3$)$_3$), 13.41 (p,

2´-C), -3.34 (p, -Si($CH_3$)$_2$), -3.92 (p, -Si($CH_3$)$_2$), -5.14 (p, -Si($CH_3$)$_2$), -5.18 (p, -Si($CH_3$)$_2$) ppm; **HRMS-ESI** ($C_{30}H_{58}O_6Si_2$): calc. for $[M+NH_4]^+$: 588.41102, found: 588.41086.

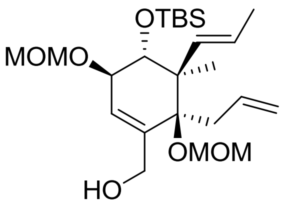


**69**

**Alcohol 69.** To a solution of silyl ether **S21** (15 mg, 26 μmol) in THF (1 ml) in a PET-falcon tube at 0 °C was added HF·pyridine (65% HF in pyridine; 12 μl). The reaction mixture was allowed to warm to room temperature and stirred for 15 h. The reaction mixture was cooled to 0 °C and additional HF·pyridine (65% HF in pyridine, 30 μl) was added. The solution was stirred another 5 h at room temperature. Aqueous saturated $NaHCO_3$-solution (10 ml) was slowly added and the resulting two layers were separated. The aqueous layer was extracted with EtOAc (3 × 10 ml) and the combined organic layers were washed with brine (20 ml), dried ($MgSO_4$) and evaporated under reduced pressure. Purification by flash chromatography on silica (petroleum ether/EtOAc 4:1) gave alcohol **69** (10 mg, 83%) as a colourless oil.

**R*f*** = 0.38 (EtOAc:petroleum ether = 1:3); $[\alpha]_D^{20}$ = +39.0 (*c* = 1.70 in $CHCl_3$); **$^1$H NMR** (400 MHz, $CDCl_3$) δ = 5.92 (d, *J* = 2.6 Hz, 1H, 9-H), 5.85 – 5.68 (m, 1H, 3´-H), 5.57 (d, *J* = 15.5 Hz, 1H, 17-H), 5.48 (ddd, *J* = 15.5, 11.8, 5.7 Hz, 1H, 5´-H), 5.17 – 5.06 (m, 3H, 4´-$H_a$, 4´-$H_b$, -OC$H_2$OMe), 4.70 (d, *J* = 6.7 Hz, 1H, -OC$H_2$OMe), 4.66 (d, *J* = 6.7 Hz, 1H, -OC$H_2$OMe), 4.49 (d, *J* = 7.3 Hz, 1H, -OC$H_2$OMe), 4.30 (d, *J* = 12.2 Hz, 1H, 1´-$H_a$), 3.93 – 3.86 (m, 2H, 1´-$H_b$, 12-H), 3.81 (dd, *J* = 7.6, 2.6 Hz, 1H, 11-H), 3.38 (s, 3H, -OC$H_3$), 3.36 (s, 3H, -OC$H_3$), 2.75 (d, *J* = 7.4 Hz, 2H, 15-$H_a$, 15-$H_b$), 1.71 (d, *J* = 5.9 Hz, 3H, 6´-H), 1.07 (s, 3H, 2´-H), 0.80 (s, 9H, -SiC(C$H_3$)$_3$), -0.03 (s, 3H, -Si(C$H_3$)$_2$), -0.06 (s, 3H, -Si(C$H_3$)$_2$) ppm; **$^{13}$C NMR** (101 MHz, $CDCl_3$) δ = 140.65 (q, 8-C), 135.15 (t, 3´-C), 135.03 (t, 17-C), 128.43 (t, 9-C), 127.04 (t, 5´-C), 117.55 (s, 4´-C), 98.73 (s, -$C$H$_2$-OMe), 92.04 (s, -$C$H$_2$-OMe), 86.86 (q, 14-C), 80.98 (t, 11-C), 75.35 (t, 12-C), 64.68 (s, 1´-C), 56.11 (p, -O$C$H$_3$), 55.58 (p, -O$C$H$_3$), 52.76 (q, 13-C), 38.49 (s, 15-C), 26.18 (p, 3C, -SiC($C$H$_3$)$_3$), 19.07 (p, 6´-C), 18.33 (q, -Si$C$(CH$_3$)$_3$), 13.33 (p, 2´-C), -3.38 (p, -Si($C$H$_3$)$_2$), -3.96 (p, -Si($C$H$_3$)$_2$) ppm; **HRMS-ESI** ($C_{24}H_{44}O_6Si$): calc. for $[M+Na]^+$: 479.27994, found: 479.27897.

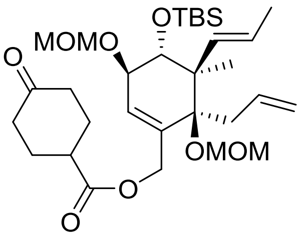


**70**

**Ester 70.** To as stirred solution of alcohol **69** (17 mg, 37 μmol) in $CH_2Cl_2$ (5 ml) were added DMAP (1 mg, 9 μmol), EDC·HCl (10 mg, 52 μmol) and 4-oxocyclohexanecarboxylic acid **25** (7.0 mg, 48 μmol). The reaction mixture was stirred for 24 h at room temperature. 1 M HCl (10 mL) was added and the resulting two layers were separated. The organic layer was washed with aqueous saturated $NaHCO_3$-solution (10 ml) and $H_2O$ (2 × 10 ml). The combined aqueous layers were extracted $CH_2Cl_2$ (20 ml) and the combined organic layers were dried ($MgSO_4$) and evaporated under reduced pressure. Purification by flash chromatography on silica (petroleum ether/EtOAc 4:1) gave ester **70** (18 mg, 83%) as colourless oil.

**R$_f$** = 0.40 (petroleum ether:EtOAc = 4:1); $[\boldsymbol{\alpha}]_{\mathbf{D}}^{\mathbf{20}}$ = +19.1 (*c* = 1.03 in $CHCl_3$); **$^1$H NMR** (500 MHz, $CDCl_3$) δ = 5.90 – 5.87 (m, 1H, 9-H), 5.85 – 5.73 (m, 1H, 3´-H), 5.57 (dd, *J* = 15.8, 1.0 Hz, 1H, 17-H), 5.48 (dq, *J* = 16.0, 6.2 Hz, 1H, 5´-H), 5.18 – 5.13 (m, 1H, 4´-$H_a$), 5.12 – 5.09 (m, 1H, 4´-$H_b$), 5.07 (d, *J* = 7.5 Hz, 1H, -OC$H_2$OC$H_3$), 4.72 (bs, 2H, 1´-H), 4.70 (d, *J* = 6.8 Hz, 1H, -OC$H_2$OC$H_3$), 4.66 (d, *J* = 6.8 Hz, 1H, -OC$H_2$OC$H_3$), 4.44 (d, *J* = 7.5 Hz, 1H, -OC$H_2$OC$H_3$), 3.92 (d, *J* = 7.6 Hz, 1H,12-H), 3.80 – 3.76 (m, 1H, 11-H), 3.36 (s, 3H, -OC$H_3$), 3.33 (s, 3H, -OC$H_3$), 2.80 – 2.69 (m, 3H, 15-H, cyclohexyl), 2.51 – 2.42 (m, 2H, cyclohexyl), 2.37 -2.28 (m, 2H, cyclohexyl), 2.25 – 2.16 (m, 2H, cyclohexyl), 2.09 – 1.97 (m, 2H, cyclohexyl), 1.70 (dd, *J* = 6.2, 1.0 Hz, 3H, 6´-H), 1.03 (s, 3H, 2´-H), 0.80 (s, 9H, -SiC(C$H_3$)$_3$), -0.04 (s, 3H, -Si(C$H_3$)$_2$), -0.07 (s, 3H, -Si(C$H_3$)$_2$) ppm; **$^{13}$C NMR** (126 MHz, $CDCl_3$) δ = 210.17 (q, C=O), 173.86 (q, -OC=O), 136.72 (q, 8-C), 135.28 (t, 3´-C), 134.96 (t, 17-C), 127.44 (t, 9-C), 126.98 (t, 5´-C), 117.57 (s, 4-C), 98.91 (s, -O*C*$H_2$OC$H_3$), 91.92 (s -O*C*$H_2$OC$H_3$), 84.73 (q, 14-C), 81.16 (t, 11-C), 75.11 (t, 12-C), 64.09 (s, 1´-C), 56.08 (p, -OC$H_2$O*C*$H_3$), 55.56 (p, -OC$H_2$O*C*$H_3$), 52.77 (q, 13-C), 41.06 (s, 15-C), 39.95 (t, cyclohexyl), 38.49 (s, 2C, cyclohexyl), 28.71 (s, 2C, cyclohexyl), 26.16 (p, 3C, -SiC(*C*$H_3$)$_3$), 19.04 (p, 6´-C), 18.32 (q, -Si*C*(C$H_3$)$_3$), 13.37 (p, 2´-C), -3.40 (p, -Si(*C*$H_3$)$_2$), -3.98 (p, -Si(*C*$H_3$)$_2$) ppm; **HRMS-ESI** ($C_{31}H_{52}O_8Si$): calc. for $[M+Na]^+$: 603.33237, found: 603.33182.

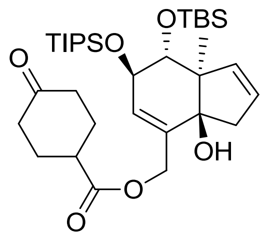


**S22**

**Silyl ether S22.** To a solution of diol **71** (21 mg, 47 μmol) in pyridine (2 ml) were added DMAP (6.8 mg, 56 μmol) and TIPSOTf (50 μl, 0.19 mmol). The reaction mixture was stirred for 16 h at room temperature. Additional TIPSOTf (100 μl, 373 μmol) was added and the reaction mixture was stirred for another 6 h at room temperature. The excess of TIPSOTf was quenched with MeOH (5 ml). The resulting solution was stirred for 20 min at room temperature and then 1 M HCl (10 ml) and $CH_2Cl_2$ (10 ml) were added. The two layers were separated and the organic layer was washed with a 1:1 mixture of aqueous saturated $NaHCO_3$-solution and brine (10 ml). The aqueous layer was extracted with $CH_2Cl_2$ (2 × 10 ml) and the combined organic layers were dried ($MgSO_4$) and evaporated under reduced pressure. The crude product was solved in $CH_2Cl_2$ (2 ml) and concentrated acetic acid (13 μl) was added. The resulting solution was stirred for 2.5 h at room temperature and then evaporated under reduced pressure. Purification by flash chromatography on silica (petroleum ether/EtOAc 20:1 to 7:1) gave silylether **S22** (17 mg, 60%) as a colourless oil.

**R$_f$** = 0.31 (EtOAc:petroleum ether = 1:4); $[\boldsymbol{\alpha}]_{\mathbf{D}}^{\mathbf{20}}$ = –58.4 (*c* = 1.60 in $CHCl_3$); **$^1$H NMR** (400 MHz, $CDCl_3$) δ = 5.79 – 5.75 (m, 1H, 9-H), 5.66 – 5.62 (m, 1H, 17-H), 5.26 – 5.22 (m, 1H, 16-H), 4.83 (d, *J* = 12.8 Hz, 1H, 1´-H$_a$), 4.64 (d, *J* = 12.8 Hz, 1H, 1´-H$_b$), 4.10 – 4.07 (m, 1H, 11-H), 3.70 (d, *J* = 3.2 Hz, 1H, 12-H), 2.72 – 2.63 (m, 1H, cyclohexyl), 2.55 – 2.36 (m, 4H, cyclohexyl), 2.32 – 2.22 (m, 2H, cyclohexyl), 2.19 – 2.08 (m, 2H, cyclohexyl), 2.02 – 1.89 (m, 3H, 2 x cyclohexyl, -OH), 1.07 (s, 3H, 2´-H), 1.00 - 0.96 (m, 21H, -Si(CH(C*H*$_3$)$_2$)$_3$, -Si(C*H*(CH$_3$)$_2$)$_3$), 0.79 (s, 9H, -SiC(C*H*$_3$)$_3$), 0.03 – -0.01 (m, 6H, -Si(C*H*$_3$)$_2$) ppm; **$^{13}$C NMR** (101 MHz, $CDCl_3$) δ = 210.18 (q, *C*=O), 173.92 (q, *C*(O)OR), 139.68 (t, 17-C), 138.66 (q, 8-C), 126.95 (t, 9-C), 122.59 (t, 16-C), 80.84 (q, 14-C), 76.64 (t, 12-C), 69.11 (t, 11-C), 64.40 (s, 1´-C), 53.02 (q, 13-C), , 41.03 (s, 15-C), 39.98 (t, cyclohexyl), 39.94 (s, 2C, cyclohexyl), 28.76 (s, cyclohexyl), 28.66 (s, cyclohexyl), 26.11 (p, 3C, SiC(*C*H$_3$)$_3$), 18.98 (p, 2´-C), 18.40, 18.30 (p, 6C, -Si(CH(*C*H$_3$)$_2$)$_3$), 18.19 (q, Si*C*(CH$_3$)$_3$), 12.82 (t, 3C, Si(C*H*(CH$_3$)$_2$)$_3$), -4.12 (p, Si(*C*H$_3$)$_2$), -4.42 (p, Si(*C*H$_3$)$_2$) ppm; **HRMS-ESI** ($C_{33}H_{58}O_6Si_2$): calc. for $[M+NH_4]^+$: 624.41102, found: 624.41085.

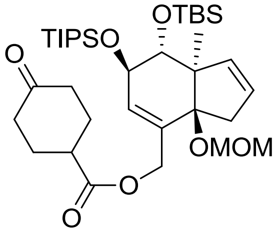

**72**

**MOM ether 72.** To a solution of alcohol **S22** (16 mg, 26 μmol) in THF (2 ml) in a Schlenk-tube were added DIPEA (38 μl, 0.21 mmol), MOMCl (10 μl, 0.13 mmol) and NaI (8.0 mg, 53 μmol). The reaction mixture was stirred for 5 min at room temperature and 19 h at 50 °C in a sealed tube. DIPEA (80 μl, 0.44 mmol) and MOMCl (50 μl, 0.65 mmol) were added and the reaction mixture was stirred another 6 h at 50 °C. The reaction was quenched with $H_2O$ (5 ml) and EtOAc (5 ml) and the resulting two layers were separated. The aqueous layer was extracted with EtOAc (2 × 10 ml) and the combined organic layers were washed with brine (20 ml), dried ($MgSO_4$) and evaporated under reduced pressure. Purification by flash chromatography on silica (petroleum ether/EtOAc 5:1) gave MOM ether **72** (5.0 mg, 29%) and alcohol **S22** (6.5 mg, 41%) as colourless oils.

$\mathbf{R}_f$ = 0.30 (EtOAc:petroleum ether = 1:7); $[\alpha]_D^{20}$ = –77.2 (*c* = 0.50 in $CHCl_3$); **$^1$H NMR** (400 MHz, $CDCl_3$) δ = 6.02 (d, *J* = 4.6 Hz, 1H, 9-H), 5.55 – 5.51 (m, 1H, 17-H), 5.31 – 5.27 (m, 1H, 16-H), 4.86 (d, *J* = 6.6 Hz, 1H, -OC$H_2$OC$H_3$), 4.81 (d, *J* = 13.3 Hz, 1H, 1´-$H_a$), 4.72 (d, *J* = 13.3 Hz, 1H, 1´-$H_b$), 4.55 (d, *J* = 6.6 Hz, 1H, -OC$H_2$OC$H_3$), 4.21 (dd, *J* = 4.6, 3.3 Hz, 1H, 11-H), 3.64 (d, *J* = 3.3 Hz, 1H, 12-H), 3.34 (s, 3H, -OC$H_3$), 2.83 – 2.76 (m, 1H, 15-$H_a$), 2.76 – 2.69 (m, 1H, cyclohexyl), 2.68 – 2.61 (m, 1H, 15-$H_b$), 2.51 – 2.42 (m, 2H, cyclohexyl), 2.38 – 2.27 (m, 2H, cyclohexyl), 2.24 – 2.15 (m, 2H, cyclohexyl), 2.07 – 1.95 (m, 2H, cyclohexyl), 1.04 (s, 3H, 2´-H), 1.04 – 0.99 (m, 21H, -Si(CH(C$H_3$)$_2$)$_3$, -Si(C*H*(CH$_3$)$_2$)$_3$), 0.87 (s, 9H, -SiC(C$H_3$)$_3$), 0.09 (s, 3H, -Si(C$H_3$)$_2$), 0.06 (s, 3H, -Si(C$H_3$)$_2$) ppm; **$^{13}$C NMR** (101 MHz, $CDCl_3$) δ = 210.18 (q, *C*=O), 173.98 (q, -*C*(O)OR), 140.37 (t, 17-C), 136.87 (q, 8-C), 136.46 (t, 16-C), 130.08 (t, 9-C), 94.32 (s, -O*C*H$_2$OCH$_3$), 86.48 (q, 14-C), 77.44 (t, 12-C), 69.51 (t, 11-C), 64.10 (s, 1´-C), 56.07 (p, -OCH$_3$), 55.24 (q, 13-C), 43.77 (s, 15-C), 41.07 (t, cyclohexyl), 39.98 (s, 2C, cyclohexyl), 28.74 (s, 2C, cyclohexyl), 26.26 (p, 3C, SiC(*C*H$_3$)$_3$), 18.44, 18.35 (7C, -Si(CH(*C*H$_3$)$_2$)$_3$, -Si*C*(CH$_3$)$_3$), 12.97 (4C, Si(C*H*(CH$_3$)$_2$)$_3$, 2´-C), -4.06 (p, Si(*C*H$_3$)$_2$), -4.11 (p, Si(*C*H$_3$)$_2$) ppm; **HRMS-ESI** ($C_{35}H_{62}O_7Si_2$): calc. for $[M+H]^+$: 651.41068, found: 651.41002.

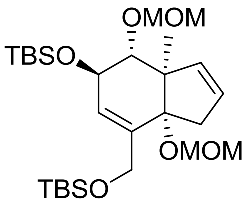


**76**

***Bis*-MOM ether 76.** To a solution of diol **75** (348 mg, 790 µmol) in THF (5 ml) in a Schlenk-tube were added DIPEA (4.23 ml, 23.7 mmol), MOMCl (1.2 ml, 15.8 mmol) and NaI (591 mg, 3.95 mmol). The Schlenk-tube was sealed and the reaction mixture was heated for 20 h at 65 °C. The reaction was quenched with $H_2O$ (10 ml) and EtOAc (10 ml) and the resulting two layers were separated. The aqueous layer was extracted with EtOAc (3 × 10 ml) and the combined organic layers were washed with brine (20 ml), dried ($MgSO_4$) and evaporated under reduced pressure. Purification by flash chromatography on silica (petroleum ether/EtOAc 20:1 to 15:1) gave **76** (373 mg, 89%) as a white solid.

$\mathbf{R}_f$ = 0.38 (petroleum ether:EtOAc = 15:1); $[\alpha]_D^{20}$ = –14.3 (*c* = 1.06 in $CHCl_3$); **$^1$H NMR** (400 MHz, $CDCl_3$) δ = 5.80 – 5.76 (m, 1H, 17-H), 5.72 – 5.68 (m, 1H, 16-H), 5.68 – 5.66 (m, 1H, 9-H), 4.81 (d, *J* = 6.5 Hz, 1H, -OC$H_2$OC$H_3$), 4.70 (d, *J* = 6.8 Hz, 1H, -OC$H_2$OCH$_3$), 4.62 (d, *J* = 6.5 Hz, 1H, -OC$H_2$OCH$_3$), 4.52 (d, *J* = 6.8 Hz, 1H, -OC$H_2$OCH$_3$), 4.42 – 4.35 (m, 1H, 1´-H$_a$), 4.29 – 4.25 (m, 1H, 11-H), 4.13 – 4.06 (m, 1H, 1´-H$_b$), 3.36 (s, 3H, -OC$H_3$), 3.34 (s, 3H, -OC$H_3$), 3.18 (d, *J* = 8.2 Hz, 1H, 12-H), 2.93 – 2.86 (m, 1H, 15-H$_a$), 2.68 – 2.62 (m, 1H, 15-H$_b$), 1.08 (s, 3H, 2´-H), 0.90 (s, 9H, -SiC(C$H_3$)$_3$), 0.88 (s, 9H, -SiC(C$H_3$)$_3$), 0.09 (s, 3H, -Si(C$H_3$)$_2$), 0.07 (s, 3H, -Si(C$H_3$)$_2$), 0.05 (s, 6H, -Si(C$H_3$)$_2$) ppm; **$^{13}$C NMR** (101 MHz, $CDCl_3$) δ = 139.78 (t, 17-C), 139.47 (q, 8-C), 126.33 (t, 16-C), 124.98 (t, 9-C), 98.78 (s, -O*C*H$_2$OCH$_3$), 93.35 (s, -O*C*H$_2$OCH$_3$), 88.31 (q, 14-C), 86.30 (t, 12-C), 70.60 (t, 11-C), 61.99 (s, 1´-C), 57.87 (q, 13-C), 56.23 (p, -OCH$_2$O*C*H$_3$), 55.93 (p, -OCH$_2$O*C*H$_3$), 42.32 (s, 15-C), 26.15 (p, 3C, -SiC(*C*H$_3$)$_3$), 26.09 (p, 3C, -SiC(*C*H$_3$)$_3$), 18.52 (q, -Si*C*(CH$_3$)$_3$), 18.31 (q, -Si*C*(CH$_3$)$_3$), 13.49 (p, 2´-C), -4.40 (p, -Si(*C*H$_3$)$_2$), -4.49 (p, -Si(*C*H$_3$)$_2$), -5.16 (p, -Si(*C*H$_3$)$_2$), -5.17 (p, -Si(*C*H$_3$)$_2$) ppm; **HRMS-ESI** ($C_{27}H_{52}O_6Si_2$): calc. for $[M+Na]^+$: 551.31946, found: 551.31849.

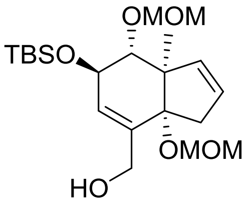


**S23**

**Alcohol S23.** To a solution of silyl ether **76** (365 mg, 690 μmol) in THF (13 ml) in a PET-falcon tube at 0 °C was added HF·pyridine (65% HF in pyridine; 270 μl). The reaction mixture was stirred for 1 h at 0 °C and 16 h at room temperature. The solution was cooled to 0 °C and HF·pyridine (65% HF in pyridine; 135 μl) was added. The reaction mixture was stirred for 1 h at 0 °C and 6 h at room temperature. Aqueous saturated $NaHCO_3$-solution (15 ml) and EtOAc (10 ml) were added and the resulting two layers were separated. The aqueous layer was extracted with EtOAc (3 × 20 ml) and the combined organic layers were washed with brine (30 ml), dried ($MgSO_4$) and evaporated under reduced pressure. Purification by flash chromatography on silica (petroleum ether/EtOAc 3:1) gave the alcohol **S23** (217 mg, 76%) as a colourless oil.

**R*f*** = 0.46 (petroleum ether:EtOAc = 2:1); $[\alpha]_D^{20}$ = –46.2 (*c* = 1.23 in $CHCl_3$); **$^1$H NMR** (500 MHz, $CDCl_3$) δ = 5.75 – 5.72 (m, 1H, 17-H), 5.70 – 5.67 (m, 1H, 9-H), 5.65 – 5.61 (m, 1H, 16-H), 4.76 (d, *J* = 6.6 Hz, 1H, -OC$H_2$OCH$_3$), 4.65 (s, 2H, -OC$H_2$OCH$_3$), 4.59 (d, *J* = 6.6 Hz, 1H, -OC$H_2$OCH$_3$), 4.26 – 4.22 (m, 1H, 11-H), 4.19 (d, *J* = 13.0 Hz, 1H, 1´-H$_a$), 4.04 (d, *J* = 13.0 Hz, 1H, 1´-H$_b$), 3.36 (s, 3H, -OC$H_3$), 3.34 (s, 3H, -OC$H_3$), 3.21 (d, *J* = 7.5 Hz, 1H, 12-H), 2.92 – 2.86 (m, 1H, 15-H$_a$), 2.76 (bs, 1H, OH), 2.63 – 2.57 (m, 1H, 15-H$_b$), 1.07 (s, 3H, 2´-H), 0.86 (s, 9H, -SiC(C$H_3$)$_3$), 0.07 (s, 3H, -Si(C$H_3$)$_2$), 0.06 (s, 3H, -Si(C$H_3$)$_2$) ppm; **$^{13}$C NMR** (126 MHz, $CDCl_3$) δ = 139.92 (q, 8-C), 139.90 (t, 17-C), 129.46 (t, 9-C), 125.50 (t, 16-C), 98.58 (s, -O*C*H$_2$OCH$_3$), 93.61 (s, -O*C*H$_2$OCH$_3$), 89.41 (q, 14-C), 85.50 (t, 12-C), 69.91 (t, 11-C), 63.29 (s, 1´-C), 57.20 (q, 13-C), 56.20 (p, -OCH$_2$O*C*H$_3$), 56.01 (p, -OCH$_2$O*C*H$_3$), 42.77 (s, 15-C), 26.05 (p, 3C, -SiC(*C*H$_3$)$_3$), 18.23 (q, -Si*C*(CH$_3$)$_3$), 14.25 (p, 2´-C), -4.43 (p, -Si(*C*H$_3$)$_2$), -4.44 (p, -Si(*C*H$_3$)$_2$) ppm; **HRMS-ESI** ($C_{21}H_{38}O_6Si$): calc. for $[M+Na]^+$: 437.23299, found: 437.23280.

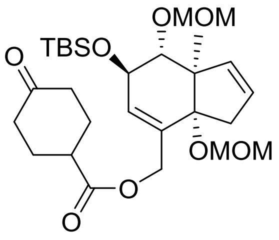

**77**

**Ester 77.** To a solution of the alcohol **S23** (51 mg, 0.12 mmol) in $CH_2Cl_2$ (5 ml) were added EDC·HCl (33 mg, 0.17 mmol), 4-oxocyclohexanecarboxylic acid (21 mg, 0.15 mmol) and DMAP (3.8 mg, 31 µmol). The reaction mixture was stirred for 19 h at room temperature. 1 M HCl (5 ml) was added and the resulting two layers were separated. The organic layer was washed with aqueous saturated $NaHCO_3$-solution (5 ml) and $H_2O$ (2 × 5 ml). The combined aqueous layers were extracted with $CH_2Cl_2$ (10 ml) and the combined organic layers were dried ($MgSO_4$) and evaporated under reduced pressure. Purification by flash chromatography on silica (petroleum ether/EtOAc 3:1) gave ester **77** (61 mg, 92%) as a white solid.

$\mathbf{R}_f$ = 0.26 (petroleum ether:EtOAc = 4:1); $[\alpha]_D^{20}$ = –22.7 (*c* = 1.0 in $CHCl_3$); **$^1$H NMR** (400 MHz, $CDCl_3$) δ = 5.78 (dd, *J* = 6.0, 1.5 Hz, 1H, 17-H), 5.72 – 5.68 (m, 1H, 16-H), 5.58 (bs, 1H, 9-H), 4.79 (d, *J* = 6.5 Hz, 1H, -OC$H_2$OCH$_3$), 4.72 (s, 2H, 1´-H), 4.71 (d, *J* = 7.0 Hz, 1H, -OC$H_2$OCH$_3$), 4.60 (d, *J* = 6.5 Hz, 1H, -OC$H_2$OCH$_3$), 4.54 (d, *J* = 7.0 Hz, 1H, -OC$H_2$OCH$_3$), 4.25 (dd, *J* = 8.0, 1.5 Hz, 1H, 11-H), 3.35 (s, 3H, -OCH$_2$OC$H_3$), 3.34 (s, 3H, -OCH$_2$OC$H_3$), 3.21 (d, *J* = 8.0 Hz, 1H, 12-H), 2.94 (dd, *J* = 17.9, 1.5 Hz, 1H, 15-$H_a$), 2.80 – 2.72 (m, 1H, cyclohexyl), 2.75 – 2.68 (m, 1H, 15-$H_b$), 2.51 – 2.41 (m, 2H, cyclohexyl), 2.39 – 2.27 (m, 2H, cyclohexyl), 2.26 – 2.16 (m, 2H, cyclohexyl), 2.08 – 1.95 (m, 2H, cyclohexyl), 1.08 (s, 3H, 2´-H), 0.87 (s, 9H, -SiC(C$H_3$)$_3$), 0.07 (s, 3H, -Si(C$H_3$)$_2$), 0.06 (s, 3H, -Si(C$H_3$)$_2$) ppm; **$^{13}$C NMR** (101 MHz, $CDCl_3$) δ = 209.99 (q, *C*=O), 173.78 (q, -*C*(O)OR), 139.76 (t, 17-C), 135.58 (q, 8-C), 128.86 (t, 9-C), 126.18 (t, 16-C), 98.68 (s, -OC$H_2$OCH$_3$), 93.53 (s, -OC$H_2$OCH$_3$), 88.03 (q, 14-C), 85.55 (t, 12-C), 70.07 (t, 11-C), 64.03 (s, 1´-C), 57.63 (q, 13-C), 56.27 (p, -OCH$_2$OC$H_3$), 56.14 (p, -OCH$_2$OC$H_3$), 42.23 (s, 15-C), 41.07 (t, cyclohexyl), 39.94 (s, 2C, cyclohexyl), 28.77 (s, cyclohexyl), 28.74 (s, cyclohexyl), 26.01 (p, 3C, -SiC(*C*H$_3$)$_3$), 18.27 (q, -Si*C*(CH$_3$)$_3$), 13.65 (p, 2´-C), -4.39 (p, -Si(*C*H$_3$)$_2$), -4.57 (p, -Si(*C*H$_3$)$_2$) ppm; **HRMS-ESI** ($C_{28}H_{46}O_8Si$): calc. for $[M+NH_4]^+$: 556.33002, found: 556.32963.

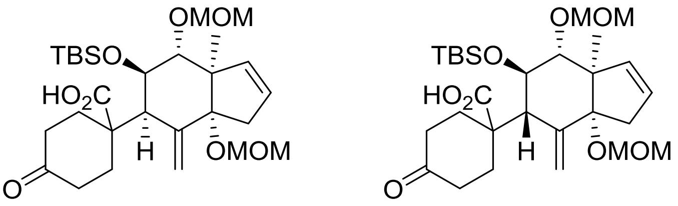

**78** **79**

**Carboxylic acids 78 and 79.** A mixture of TMSCl (285 µl, 2.25 mmol) and $NEt_3$ (285 µl, 2.05 mmol) in toluene (1 ml) was centrifuged at 10 000 rpm for 5 min at room temperature. The supernatant (1 ml) of this centrifuged mixture was slowly added to a solution of allyl ester **77** (60.5 mg, 112 µmol) in toluene (4 ml) in a Schlenk-tube at –78 °C. LiHMDS (1 M solution in THF, 369 µl, 369 µmol) was slowly added. The reaction mixture was stirred for 1 h at –78 °C then gradually warmed to 65 °C within 6 h. In a sealed tube the reaction mixture was then stirred for 43 h at 65 °C. The reaction mixture was allowed to cool to room temperature, 1 M HCl (10 ml) was added and the resulting solution was stirred for 45 min at room temperature. The two layers were separated and the aqueous layer was extracted with EtOAc (2 × 20 ml) and the combined organic layers were washed with brine (20 ml), dried ($MgSO_4$) and evaporated under reduced pressure, to give the crude product as a mixture of diastereomers (dr ca. 1.3:1, calculated from the $^1$H NMR of the crude product in $CDCl_3$). Purification by flash chromatography on silica (petroleum ether/EtOAc 3:1; 0.1% acetic acid to petroleum ether/EtOAc 1:1; 0.1% acetic acid) gave the carboxylic acids **78** and **79** (47 mg, 78% combined yield).

**78:** $\mathbf{R}_f$ = 0.42 (EtOAc:petroleum ether = 3:1; acetic acid); $[\boldsymbol{\alpha}]_{\mathbf{D}}^{\mathbf{20}}$ = +1.9 (*c* = 1.60 in $CHCl_3$); **$^1$H NMR** (600 MHz, $CDCl_3$) δ = 5.48 – 5.45 (m, 1H, 16-H), 5.39 (s, 1H, 1´-$H_a$), 5.40 – 5.36 (m, 1H, 17-H), 5.38 (s, 1H, 1´-$H_b$), 4.72 (d, *J* = 6.9 Hz, 1H, -OC$H_2$OC$H_3$), 4.66 (d, *J* = 7.5 Hz, 1H, -OC$H_2$OC$H_3$), 4.65 (d, *J* = 6.9 Hz, 1H, -OC$H_2$OC$H_3$), 4.43 (d, *J* = 7.5 Hz, 1H, -OC$H_2$OC$H_3$), 4.15 (d, *J* = 3.0 Hz, 1H, 11-H), 3.57 (d, *J* = 3.0 Hz, 1H, 12-H), 3.41 (s, 3H, -OC$H_3$), 3.38 (s, 3H, -OC$H_3$), 3.25 (bs, 1H, 9-H), 2.86 (dd, *J* = 16.1, 3.2 Hz, 1H, 15-$H_a$), 2.85 – 2.78 (m, 1H, cyclohexyl), 2.64 – 2.57 (m, 2H, 15-$H_b$, cyclohexyl), 2.43 – 2.24 (m, 4H, cyclohexyl), 2.07 – 1.95 (m, 2H, cyclohexyl), 1.18 (s, 3H, 2´-H), 0.79 (s, 9H, -SiC(C$H_3$)$_3$), 0.12 (s, 3H, -Si(C$H_3$)$_2$), 0.04 (s, 3H, -Si(C$H_3$)$_2$) ppm; **$^{13}$C NMR** (151 MHz, $CDCl_3$) δ = 211.20 (q, *C*=O), 179.37 (q, *C*$O_2$H), 141.69 (q, 8-C), 139.80 (t, 17-C), 125.97 (t, 16-C), 115.08 (s, 1´-C), 97.95 (s, -O*C*$H_2$OC$H_3$), 91.81 (s, -O*C*$H_2$OC$H_3$), 89.86 (q, 14-C), 81.39 (t, 12-C), 71.82 (t, 11-C), 56.42 (p, -O*C*$H_3$), 55.48 (p, -O*C*$H_3$), 54.78 (q, 13-C), 48.26 (q, cyclohexyl), 45.71 (t, 9-C), 39.07 (s, cyclohexyl), 38.08 (s, cyclohexyl), 36.71 (s, 15-C),

35.61 (s, cyclohexyl), 30.24 (s, cyclohexyl), 26.17 (p, 3C, -SiC(*C*$H_3$)$_3$), 18.67 (p, 2´-C), 18.14 (q, -Si*C*($CH_3$)$_3$), -1.58 (p, -Si(*C*$H_3$)$_2$), -5.04 (p, -Si(*C*$H_3$)$_2$) ppm; **HRMS-ESI** ($C_{28}H_{46}O_8Si$): calc. for $[M+Na]^+$: 561.28542, found: 561.28499.

**79:** $\mathbf{R_f}$ = 0.45 (EtOAc:petroleum ether = 3:1; acetic acid); $[\alpha]_D^{20}$= –30.0 (*c* = 1.22 in $CHCl_3$); **$^1$H NMR** (500 MHz, $CDCl_3$) δ = 5.58 – 5.53 (m, 2H, 16-H, 17-H), 5.07 (s, 1H, 1´-$H_a$), 4.98 (s, 1H, 1´-$H_b$), 4.76 (d, *J* = 7.3 Hz, 1H, -OC*H*$_2$OCH$_3$), 4.71 (d, *J* = 7.3 Hz, 1H, -OC*H*$_2$OCH$_3$), 4.70 (d, *J* = 7.0 Hz, 1H, -OC*H*$_2$OCH$_3$), 4.58 (d, *J* = 7.0 Hz, 1H, -OC*H*$_2$OCH$_3$), 4.34 (dd, *J* = 7.1, 3.2 Hz, 1H, 11-H), 3.41 (s, 3H, -OCH$_2$OC*H*$_3$), 3.38 – 3.32 (m, 4H, -OCH$_2$OC*H*$_3$, 12-H), 3.28 (d, *J* = 17.5 Hz, 1H, 15-H), 2.81 (d, *J* = 7.1 Hz, 1H, 9-H), 2.65 – 2.52 (m, 3H, 15-H, cyclohexyl), 2.48 – 2.21 (m, 5H, cyclohexyl), 2.05 – 1.94 (m, 1H, cyclohexyl), 1.12 (s, 3H, 2´-H), 0.85 (s, 9H, -SiC(C*H*$_3$)$_3$), 0.16 (s, 3H, -Si(C*H*$_3$)$_2$), 0.08 (s, 3H, -Si(C*H*$_3$)$_2$) ppm; **$^{13}$C NMR** (126 MHz, $CDCl_3$) δ = 211.25 (q, *C*=O), 180.44 (q, *C*O$_2$H), 147.80* (q, 8-C), 138.67* (t, 17-C), 125.66 (t, 16-C), 109.07* (s, 1´-C), 97.88 (s, -O*C*H$_2$CH$_3$), 94.04 (s, -O*C*H$_2$CH$_3$), 89.28 (q, 14-C), 85.58 (t, 12-C), 72.87 (t, 11-C), 58.07* (q, 13-C), 55.98 (p, -O*C*H$_3$), 55.91 (p, -O*C*H$_3$), 52.70* (t, 9-C), 48.37* (q, cyclohexyl), 41.08* (s, 15-C), 38.54 (s, cyclohexyl), 38.25 (s, cyclohexyl), 34.44 (s, cyclohexyl), 29.93 (s, cyclohexyl), 26.36 (p, 3C, -SiC(*C*H$_3$)$_3$), 20.35* (p, 2´-C), 18.28 (q, -Si*C*(CH$_3$)$_3$), 1.24 (p, -Si(*C*H$_3$)$_2$), -2.07 (p, -Si(*C*H$_3$)$_2$) ppm. (* peak from HSQC and HMBC; in $^{13}$C-NMR not resolved); **HRMS-ESI** ($C_{28}H_{46}O_8Si$): calc. for $[M-H]^-$: 537.28892, found: 537.28823.

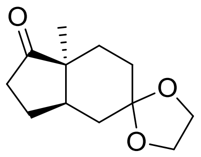

**S24**

**Ketal S24.** To a solution of Hajos–Parrish ketone **80** (90%, 13.46 g, 73.8 mmol) in THF (80 ml) was added ethylene glycol (160 ml) and Pd/C (10%, 802 mg). A catalytic amount of concentrated HCl was added to the mixture to adjust the pH value to pH 5–6. The suspension was stirred at room temperature under an atmosphere of hydrogen overnight. The mixture was diluted with $CH_2Cl_2$ (150 ml) and the catalyst was removed by filtration through Celite. Water (240 ml) was added and the aqueous phase was extracted with $CH_2Cl_2$ (3 × 150 ml). The combined organic layer was washed with water (240 ml) and brine (320 ml) and dried over $MgSO_4$. Removal of the solvent in vacuo gave the crude product. Purification by flash

chromatography on silica gel with petroleum ether/EtOAc 9:1 as eluent afforded ketal **S24** (15.08 g, 97%) as a colourless oil.

**R*f*** = 0.51 (petroleum ether:EtOAc = 2:1); $[\alpha]_D^{20}$ = +79.6 (*c* = 1.0 in $CHCl_3$); **$^1$H NMR** (400 MHz, $CDCl_3$) δ = 3.98 – 3.88 (m, 4H), 2.34 – 2.25 (m, 2H), 2.17 (dq, *J* = 8.4, 6.0 Hz, 1H), 2.04 (dt, *J* = 14.4, 7.9 Hz, 1H), 1.94 (dd, *J* = 9.7, 7.1 Hz, 1H), 1.90 – 1.77 (m, 2H), 1.64 – 1.53 (m, 1H), 1.48 – 1.36 (m, 3H), 1.05 (s, 3H) ppm; **$^{13}$C NMR** (101 MHz, $CDCl_3$) δ = 221.86, 108.58, 64.42, 64.26, 48.16, 42.36, 36.05, 34.46, 31.14, 28.12, 23.70, 21.81 ppm; **HRMS-ESI** ($C_{12}H_{18}O_3$): calc. for $[M+H]^+$: 211.13287, found: 211.13288.

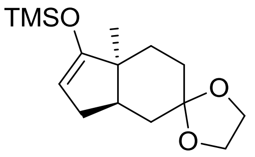


**81**

**Silyl enol ether 81.** To a solution of $iPr_2NH$ (12.0 ml, 85.0 mmol) in THF (220 ml) was added *n*-BuLi (2.5 M in hexanes, 34.0 ml, 85.0 mmol) at −78 °C and the mixture was stirred at 0 °C for 30 min. It was cooled to −78 °C and a solution of ketal **S24** (8.94 g, 42.5 mmol) in THF (90 ml) was added dropwise. After 1 h at −78 °C TMSCl (10.8 ml, 85.0 mmol) was added. The mixture was allowed to warm to room temperature and stirred for another 30 min. The reaction mixture was quenched with aqueous saturated $NaHCO_3$-solution (200 ml) and water (200 ml) and the aqueous layer was extracted with EtOAc (3 × 200 ml). The combined organic layers were washed with brine (250 ml), dried ($MgSO_4$) and evaporated under reduced pressure to give the crude product. Purification by flash chromatography on silica gel with petroleum ether/EtOAc 20:1 as eluent gave silyl enol ether **81** (12.0 g, quant.) as a colourless oil.

**R*f*** = 0.73 (petroleum ether:EtOAc = 9:1); $[\alpha]_D^{20}$ = +32.7 (*c* = 1.0 in $CHCl_3$); **$^1$H NMR** (400 MHz, $CDCl_3$) δ = 4.42 (s, 1H, 7-H), 3.96 – 3.87 (m, 4H, -O$CH_2$$CH_2$O-), 2.31 (ddd, *J* = 14.4, 6.8, 1.8 Hz, 1H, 6-$H_a$), 2.00 (td, *J* = 11.5, 7.1 Hz, 1H, 5-H), 1.94 – 1.86 (m, 1H, 6-$H_b$), 1.80 – 1.66 (m, 2H, cyclohexyl), 1.62 – 1.38 (m, 4H, cyclohexyl), 1.00 (s, 3H, -C$H_3$), 0.22 – 0.17 (m, 9H, -Si(C$H_3$)$_3$) ppm; **$^{13}$C NMR** (101 MHz, $CDCl_3$) δ = 160.05 (q, 29-C), 109.71 (q, 1-C), 97.62 (t, 7-C), 64.20 (s, -O*C*$H_2$$CH_2$O-), 64.11 (s, -O$CH_2$*C*$H_2$O-), 44.44 (q, 4-C), 42.31 (t, 5-C), 36.81 (s, cyclohexyl), 32.31 (s, 6-C), 31.36 (s, cyclohexyl), 30.01 (s, cyclohexyl),

24.09 (p, -$CH_3$), 0.15 (p, 3 C, Si($CH_3$)$_3$) ppm; **HRMS-ESI** ($C_{15}H_{26}O_3Si$): calc. for $[M+H]^+$: 283.17240, found: 283.17233.

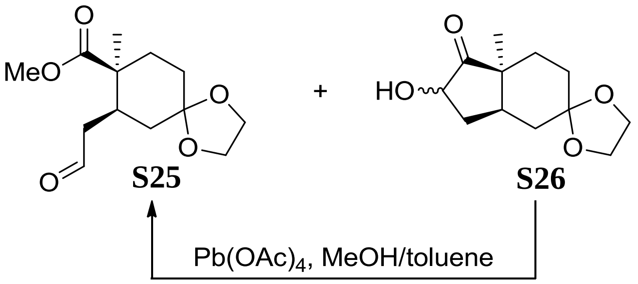


**Ester aldehyde S25:** A stream of ozone was bubbled through a solution of silyl enol ether **81** (10.76 g, 38.1 mmol) in $CH_2Cl_2$ (400 ml) at −78 °C. When the blue colour persisted, DMS (30 ml) was added. After stirring for 1 h at −78 °C, the reaction was allowed to warm to room temperature. Stirring was continued at room temperature for 1 h before the solvent was evaporated under reduced pressure. The crude residue was taken up in $CH_2Cl_2$/MeOH (1:1, 180 ml) and $TMSCHN_2$ (2 M in $Et_2O$, 21 ml, 41.9 mmol) was added dropwise. After stirring for 30 min at room temperature, a small amount of AcOH was added to quench the excess $TMSCHN_2$. Then the solvent was evaporated under reduced pressure. Purification by flash chromatography on silica gel with petroleum ether/EtOAc 6:1 to 1:1 gave ester aldehyde **S25** (5.77 g, 59%) as a colourless oil and acyloin **S26** (1.79 g, 21%, diastereomeric mixture of varying ratios) as a side product.

**S25:** $\mathbf{R_f}$ = 0.41 (petroleum ether:EtOAc = 2:1); $[\alpha]_D^{20}$ = +7.4 (*c* = 1.0 in $CHCl_3$); **$^1$H NMR** (400 MHz, $C_6D_6$) δ = 9.36 (dd, *J* = 1.9, 0.9 Hz, 1H, -C*H*O), 3.57 – 3.46 (m, 4H, -OC*H$_2$*C*H$_2$*O-), 3.21 (s, 3H, -$CO_2C$*H$_3$*), 2.47 (ddd, *J* = 18.1, 10.0, 2.0 Hz, 1H, 6-$H_a$), 2.38 – 2.30 (m, 2H, 6-$H_b$, 5-H), 2.07 – 1.99 (m, 1H, 3-$H_a$), 1.99 – 1.91 (dd, *J* = 13.2, 11.4 Hz, 1H, 10-$H_a$), 1.89 – 1.76 (m, 2H, 10-$H_b$, 2-$H_a$), 1.71 – 1.61 (m, 2H, 2-$H_b$, 3-$H_b$), 0.97 (s, 3H, -C*H$_3$*) ppm; **$^{13}$C NMR** (101 MHz, $C_6D_6$) δ = 200.42 (t, -*C*HO), 175.57 (q, -*C*$O_2CH_3$), 108.59 (q, 1-C), 64.42 (s, -O*C*$H_2CH_2O$-), 64.23 (s, -O$CH_2$*C*$H_2O$-), 51.02 (p, -$CO_2$*C*$H_3$), 46.27 (s, 6-C), 45.08 (q, 4-C), 38.02 (s, 10-C), 37.58 (t, 5-C), 35.21 (s, 6-C), 33.03 (s, 2-C), 24.76 (p, -*C*$H_3$) ppm; **HRMS-ESI** ($C_{13}H_{20}O_5$): calc. for $[M+H]^+$: 257.13835, found: 257.13825.

**S26** (major diastereomer): $\mathbf{R_f}$ = 0.18 (petroleum ether:EtOAc = 2:1); **$^1$H NMR** (400 MHz, $C_6D_6$) δ = 3.81 (t, *J* = 7.6 Hz, 1H), 3.46 – 3.39 (m, 4H), 2.06 – 1.97 (m, 3H), 1.86 – 1.77 (m, 1H), 1.64 (ddd, *J* = 14.0, 5.9, 1.4 Hz, 1H), 1.50 – 1.41 (m, 2H), 1.38 – 1.28 (m, 1H), 1.18

(ddd, $J$ = 14.0, 9.4, 0.8 Hz, 1H), 0.94 (s, 3H) ppm; **$^{13}$C NMR** (101 MHz, $C_6D_6$) δ = 220.72, 108.44, 72.47, 64.24, 64.11, 47.16, 38.68, 37.12, 32.98, 31.76, 29.48, 23.19 ppm; **HRMS-ESI** ($C_{12}H_{18}O_4$): calc. for $[M^{\bullet}]^+$: 226.11996, found: 226.12009.

Acyloin **S26** was recycled as follows: To a solution of acyloin **S26** (926 mg, 4.09 mmol) in MeOH (45 ml) and toluene (15 ml) was added portionwise $Pb(OAc)_4$ (4.54 g, 10.2 mmol) over 30 min. The mixture was stirred at room temperature for further 20 min and then quenched with water (30 ml). The aqueous phase was extracted with EtOAc and the combined organic layers were washed with water, diluted aqueous $NaHCO_3$-solution and brine, dried over $MgSO_4$ and concentrated in vacuo. Purification by flash chromatography on silica gel with petroleum ether/EtOAc 4:1 afforded ester aldehyde **S25** (678 mg, 65%).

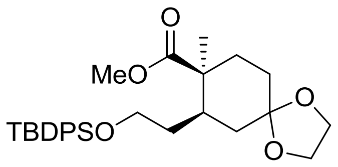


**82**

**Silyl ether 82.** To a solution of ester aldehyde **S25** (11.2 g, 43.7 mmol) in MeOH (250 ml) at 0 °C was added $NaBH_4$. After stirring for 30 min at room temperature, the reaction mixture was diluted with EtOAc (600 ml) and washed with half saturated brine (150 ml). The aqueous phase was extracted with EtOAc (2 × 300 ml) and the combined organic layers were dried ($MgSO_4$) and concentrated under reduced pressure to give the crude product, which was dried under high vacuum and used for the next step without further purification.

To a solution of the crude alcohol, imidazole (4.46 g, 65.5 mmol) and DMAP (1.07 g, 8.74 mmol) in $CH_2Cl_2$ (400 ml) was added TBDPSCl (22.7 ml, 87.4 mmol). The resulting solution was stirred at room temperature for 30 min. After addition of saturated aqueous $NH_4Cl$-solution (200 ml), the two layers were separated and the aqueous phase was extracted with $CH_2Cl_2$ (2 × 200 ml). The combined organic layers were washed with brine (300 ml), dried ($MgSO_4$) and concentrated under reduced pressure. Purification by flash chromatography on silica gel with petroleum ether/EtOAc 10:1 as eluent gave silyl ether **82** (16.8 g, 78%) as a colourless oil.

**$R_f$** = 0.35 (petroleum ether:EtOAc = 9:1); $[\alpha]_D^{20}$ = −7.4 ($c$ = 1.0 in $CHCl_3$); **$^1$H NMR** (400 MHz, $C_6D_6$) δ = 7.84 – 7.76 (m, 4H, Ar-H), 7.27 – 7.20 (m, 6H, Ar-H), 3.78 – 3.66 (m, 2H,

7-H), 3.60 – 3.47 (m, 4H, -OC$H_2$C$H_2$O-), 3.26 (s, 3H, -$CO_2$C$H_3$), 2.16 – 1.91 (m, 6H, 10-H, 6-$H_a$, 5-H, 3-$H_a$, 2-$H_a$), 1.79 – 1.64 (m, 3H, 6-$H_b$, 3-$H_b$, 2-$H_b$), 1.23 (s, 3H, -C$H_3$), 1.19 (s, 9H, -SiC(C$H_3$)$_3$) ppm; **$^{13}$C NMR** (101 MHz, $C_6D_6$) δ = 175.82 (q, -*C*$O_2CH_3$), 136.09 (2C), 136.04 (2C), 134.44, 134.42, 129.91, 129.90, 128.06 (4C) (Ar-C), 109.20 (q, 1-C), 64.38 (s, -O*C*$H_2CH_2$O-), 64.27 (s, -O$CH_2$*C*$H_2$O-), 62.88 (s, 7-C), 50.84 (p, -$CO_2$*C*$H_3$), 45.56 (q, 4-C), 40.56 (t, 5-C), 37.69 (s, 10-C), 35.77 (s, 3-C), 34.39 (s, 6-C), 32.87 (s, 2-C), 27.15 (p, 3C, -SiC(*C*$H_3$)$_3$), 25.21 (p, -*C*$H_3$), 19.52 (q, -Si*C*($CH_3$)$_3$) ppm; **HRMS-ESI** ($C_{29}H_{40}O_5Si$): calc. for $[M+H]^+$: 497.27178, found: 497.27155.

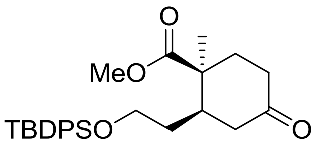


**S27**

**Ketone S27.** To a solution of silyl ether **82** (15.1 g, 30.4 mmol) in $CH_2Cl_2$ (250 ml) and water (50 ml) was added $HClO_4$ (70%, 10 ml). The mixture was stirred at room temperature until TLC showed full conversion of the starting material (ca. 6 h). Then water (200 ml) was added, the layers were separated and the aqueous phase was extracted with $Et_2O$ (2 × 200 ml). The combined organic layers were washed with water (200 ml), aqueous saturated $NaHCO_3$-solution (200 ml) and brine (200 ml), dried ($MgSO_4$) and evaporated under reduced pressure to give crude ketone **S27** which was dried under high vacuum and used for the next step without further purification.

For analytical purpose a small amount was purified by flash chromatography on silica gel with petroleum ether/EtOAc 9:1 as eluent.

$\mathbf{R}_f$ = 0.51 (petroleum ether:EtOAc = 4:1); $[\alpha]_D^{20}$ = +12.7 (*c* = 1.0 in $CHCl_3$); **$^1$H NMR** (400 MHz, $CDCl_3$) δ = 7.66 – 7.61 (m, 4H, Ar-H), 7.46 – 7.35 (m, 6H, Ar-H), 3.69 (s, 3H, -$CO_2$C$H_3$), 3.68 – 3.56 (m, 2H, 7-H), 2.53 – 2.39 (m, 3H, 10-H, 2-$H_a$), 2.37 – 2.23 (m, 2H, 2-$H_b$, 3-$H_a$), 2.07 – 1.97 (m, 1H, 5-H), 1.87 – 1.67 (m, 2H, 3-$H_b$, 6-$H_a$), 1.44 (ddd, *J* = 14.2, 9.9, 4.7 Hz, 1H, 6-$H_b$), 1.34 (s, 3H, -C$H_3$), 1.04 (s, 9H, -SiC(C$H_3$)$_3$) ppm; **$^{13}$C NMR** (101 MHz, $CDCl_3$) δ = 210.91 (q, 1-C), 176.14 (q, -*C*$O_2CH_3$), 135.67 (4C), 133.82, 133.79, 129.83, 129.81, 127.86 (2C), 127.83 (2C) (Ar-C), 61.54 (s, 7-C), 51.87 (p, -$CO_2$*C*$H_3$), 45.16 (q, 4-C), 42.61 (s, 10-C), 41.74 (t, 5-C), 38.31 (s, 2-C), 34.98 (s, 3-C), 34.37 (s, 6-C), 27.01

(p, 3C, -SiC(C$H_3$)$_3$), 24.02 (p, -*C*$H_3$), 19.33 (q, -Si*C*(C$H_3$)$_3$) ppm; **HRMS-ESI** ($C_{27}H_{36}O_4Si$): calc. for $[M+H]^+$: 453.24556, found: 453.24589.

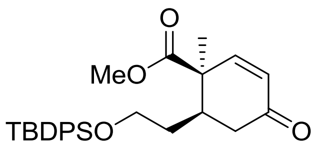


**83**

**Enone 83.** To a solution of crude ketone **S27** (30.4 mmol) in THF (300 ml) was added LiHMDS (1 M solution in THF, 45.6 ml, 45.6 mmol) at −78 °C. After stirring for 1 h at −78 °C, TMSCl (5.79 ml, 45.6 mmol) was added. The mixture was allowed to warm to room temperature and stirred for 30 min. Aqueous saturated $NaHCO_3$-solution (200 ml) and water (100 ml) were added and the aqueous phase was extracted with EtOAc (3 × 200 ml). The combined organic layers were washed with brine (250 ml), dried ($MgSO_4$) and concentrated under reduced pressure to give crude silyl enol ether, which was dried under high vacuum and used in the next step without further purification.

To a solution of the crude silyl enol ether in DMSO (80 ml) was added $Pd(OAc)_2$ (1.02 g, 4.56 mmol) at room temperature. The reaction mixture was stirred under an atmosphere of oxygen overnight and then quenched with water (250 ml). The aqueous phase was extracted with EtOAc (3 × 200 ml). The combined organic layers were washed with brine (250 ml), dried ($MgSO_4$) and concentrated under reduced pressure. Purification of the crude product by flash chromatography on silica gel with petroleum ether/EtOAc 13:1 as eluent gave enone **83** (9.81 g, 72% over 3 steps) as a white solid.

$\mathbf{R_f}$ = 0.60 (petroleum ether:EtOAc = 4:1); $[\alpha]_D^{20}$ = +57.0 (*c* = 1.0 in $CHCl_3$); **$^1$H NMR** (400 MHz, $CDCl_3$) δ = 7.71 – 7.60 (m, 4H, Ar-H), 7.47 – 7.34 (m, 6H, Ar-H), 6.79 (d, *J* = 10.1 Hz, 1H, 3-H), 6.01 (d, *J* = 10.1 Hz, 1H, 2-H), 3.69 (s, 3H, -$CO_2$C*$H_3$*), 3.75 – 3.60 (m, 2H, 7-H), 2.55 – 2.45 (m, 2H, 10-H), 2.38 – 2.29 (m, 1H, 5-H), 1.86 – 1.76 (m, 1H, 6-$H_a$), 1.45 (s, 3H, -C*$H_3$*), 1.38 – 1.28 (m, 1H, 6-$H_b$) 1.07 – 1.03 (s, 9H, -SiC(C*$H_3$*)$_3$) ppm; **$^{13}$C NMR** (101 MHz, $CDCl_3$) δ = 198.74 (q, 1-C), 173.16 (q, -*C*$O_2CH_3$), 151.46 (t, 3-C), 135.61 (2 C), 135.60 (2 C), 133.65, 133.62, 129.86, 129.83 (Ar-C), 128.96 (t, 2-C), 127.85 (2C), 127.82 (2C) (Ar-C), 61.10 (s, 7-C), 52.35 (p, -$CO_2$*C*$H_3$), 47.91 (q, 4-C), 39.67 (t, 5-C), 38.94 (s, 10-C), 33.73

(s, 6-C), 26.97 (p, 3C, -SiC(*C*H$_3$)$_3$), 23.99 (p, -*C*H$_3$), 19.28 (q, -Si*C*(CH$_3$)$_3$) ppm; **HRMS-ESI** ($C_{27}H_{34}O_4Si$): calc. for $[M-H]^-$: 449.21536, found: 449.22136; **m.p.:** 76 °C.

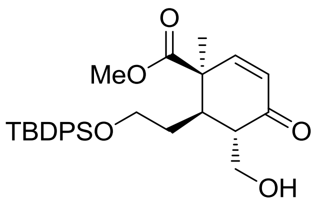

**S28**

**Alcohol S28.** To a stirred solution of *n*-BuLi (2.5 M in hexanes, 22.9 ml, 57.4 mmol) in THF (120 ml) was added dropwise iPr$_2$NH (8.11 ml, 57.4 mmol) at −10 °C. The solution was allowed to stir at 0 °C for 30 min and then cooled to −78 °C. Enone **83** (8.62 g, 19.1 mmol) in THF (25 ml) was added and stirring was continued for 1 h. 1*H*-Benzotriazole-1-methanol in THF (175 ml) was added dropwise and the reaction mixture was kept 2 h at −78 °C. The reaction was quenched with water (85 ml) and extracted with $Et_2O$ (2 × 250 ml). The combined organic layers were washed successively with 4 M NaOH (85 ml) and brine (85 ml), dried ($MgSO_4$) and concentrated in vacuo. Purification by flash chromatography on silica gel with petroleum ether/EtOAc 9:1 to 6:1 as eluent gave alcohol **S28** (6.10 g, 67%) as a colourless oil.

**R$_f$** = 0.28 (petroleum ether:EtOAc = 4:1); $[\alpha]_D^{20}$ = +134.1 (*c* = 1.0 in CHCl$_3$); **$^1$H NMR** (400 MHz, C$_6$D$_6$) δ = 7.81 – 7.72 (m, 4H, Ar-H), 7.27 – 7.20 (m, 6H, Ar-H), 5.99 (d, *J* = 10.0 Hz, 1H, 3-H), 5.87 (d, *J* = 10.0 Hz, 1H, 2-H), 4.11 (ddd, *J* = 11.3, 7.1, 2.8 Hz, 1H, -C*H*$_2$OH), 3.66 (dddd, *J* = 15.0, 10.2, 7.9, 5.9 Hz, 2H, 7-H), 3.43 (dt, *J* = 11.1, 5.4 Hz, 1H, -C*H*$_2$OH), 3.15 (s, 3H, -CO$_2$C*H*$_3$), 2.81 (ddd, *J* = 12.6, 5.0, 2.8 Hz, 1H, 10-H), 2.40 (dd, *J* = 7.0, 6.0 Hz, 1H, -CH$_2$O*H*), 1.95 (ddd, *J* = 12.6, 6.1, 2.4 Hz, 1H, 5-H), 1.90 – 1.81 (m, 1H, 6-H$_a$), 1.63 – 1.53 (m, 1H, 6-H$_b$), 1.18 (s, 3H, -C*H*$_3$), 1.15 (s, 9H, -SiC(C*H*$_3$)$_3$) ppm; **$^{13}$C NMR** (101 MHz, C$_6$D$_6$) δ = 200.85 (q, 1-C), 172.42 (q, -*C*O$_2$CH$_3$), 150.93 (t, 3-C), 136.11 (2C), 136.04 (2C), 134.03, 133.98, 130.09 (2C) (Ar-C), 129.22 (t, 2-C), 128.16 (4C, Ar-C), 63.84 (s, 7-C), 59.70 (s, -*C*H$_2$OH), 51.74, 51.73 (10-C, -CO$_2$*C*H$_3$), 48.95 (q, 4-C), 40.32 (t, 5-C), 34.32 (s, 6-C), 27.14 (p, 3C, -SiC(*C*H$_3$)$_3$), 24.43 (p, -*C*H$_3$), 19.42 (q, -Si*C*(CH$_3$)$_3$) ppm; **HRMS-ESI** ($C_{28}H_{36}O_5Si$): calc. for $[M+H]^+$: 481.24048, found: 481.24087.

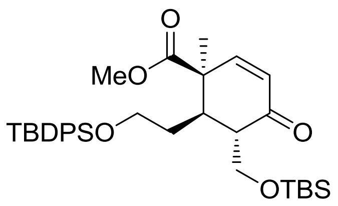


**84**

**Silyl ether 84.** To a solution of alcohol **S28** (1.39 g, 2.89 mmol) in DMF (25 ml) were added imidazole (591 mg, 8.67 mmol) and TBSCl (654 mg, 4.34 mmol). The resulting mixture was stirred at room temperature overnight. Then 1 M HCl (25 ml) and EtOAc (40 ml) were added, the two layers were separated and the aqueous phase was extracted with EtOAc (2 × 40 ml). The combined organic layers were washed with saturated aqueous $NaHCO_3$-solution (40 ml) and brine (40 ml), dried ($MgSO_4$) and concentrated under reduced pressure. Purification by flash chromatography on silica gel with petroleum ether/EtOAc 15:1 as eluent gave silylether **84** (1.55 g, 90%) as a colourless oil.

$\mathbf{R}_f$ = 0.50 (petroleum ether:EtOAc = 9:1); $[\alpha]_D^{20}$ = +103.0 (*c* = 1.0 in $CHCl_3$); **$^1$H NMR** (400 MHz, $C_6D_6$) δ = 7.83 – 7.78 (m, 4H, Ar-H), 7.29 – 7.22 (m, 6H, Ar-H), 5.99 (d, *J* = 10.0 Hz, 1H, 3-H), 5.94 (d, *J* = 10.0 Hz, 1H, 2-H), 4.43 (dd, *J* = 10.3, 2.5 Hz, 1H, -C$H_2$OTBS), 3.98 (td, *J* = 10.1, 5.3 Hz, 1H, 7-$H_a$), 3.77 (td, *J* = 9.8, 7.0 Hz, 1H, 7-$H_b$), 3.54 (dd, *J* = 10.3, 2.7 Hz, 1H, -C$H_2$OTBS), 3.16 (s, 3H, -$CO_2CH_3$), 2.70 (dt, *J* = 12.0, 2.5 Hz, 1H, 10-H), 2.17 (ddd, *J* = 11.9, 5.8, 2.5 Hz, 1H, 5-H), 2.07 – 1.96 (m, 1H, 6-$H_a$), 1.85 (ddt, *J* = 11.2, 9.7, 5.6 Hz, 1H, 6-$H_b$), 1.18 (s, 12H, -$CH_3$, -SiC($CH_3$)$_3$), 0.88 (s, 9H, -SiC($CH_3$)$_3$), 0.09 (s, 3H, -Si($CH_3$)$_2$), 0.01 (s, 3H, -Si($CH_3$)$_2$) ppm; **$^{13}$C NMR** (101 MHz, $C_6D_6$) δ = 197.60 (q, 1-C), 172.74 (q, -$CO_2CH_3$), 150.10 (t, 3-C), 136.04 (2C), 136.00 (2C), 134.34, 134.25, 130.07 (2C) (Ar-C), 130.01 (t, 2-C), 128.16 (4C, Ar-C), 64.56 (7-C), 59.57 (s, -$CH_2$OTBS), 52.47 (t, 10-C), 51.71 (p, -$CO_2CH_3$) , 48.52 (q, 4-C), 39.80 (t, 5-C), 35.25 (s, 6-C), 27.18 (p, 3C, -SiC($CH_3$)$_3$), 26.17 (p, 3C, -SiC($CH_3$)$_3$), 24.33 (p, -$CH_3$), 19.43 (q, -SiC($CH_3$)$_3$), 18.50 (q, -SiC($CH_3$)$_3$), -5.26 (p, -Si($CH_3$)$_2$), -5.29 (p, -Si($CH_3$)$_2$) ppm; **HRMS-ESI** ($C_{34}H_{50}O_5Si_2$): calc. for $[M+H]^+$: 595.32695, found: 595.32966.

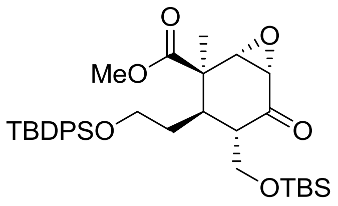

**S29**

**Epoxide S29.** To a solution of compound **84** (1.50 g, 2.52 mmol) in MeOH (50 ml) were added 6 M NaOH (1.7 ml, 10.1 mmol) and $H_2O_2$ (35% in $H_2O$, 0.81 ml, 7.56 mmol) at 0 °C. After stirring for 1 h at 0 °C half saturated aqueous NaCl-solution containing $Na_2S_2O_3$ (50 ml) was added. The aqueous phase was extracted with EtOAc (3 × 50 ml) and the combined organic layers were washed with brine (70 ml), dried over $MgSO_4$ and the solvents were evaporated in vacuo. The crude product was purified by flash chromatography on silica gel with petroleum ether/EtOAc 15:1 as eluent to afford epoxide **S29** (1.23 g, 80%) as a colourless oil.

$\mathbf{R}_f$ = 0.42 (petroleum ether:EtOAc = 9:1); $[\alpha]_D^{20}$ = −26.1 (*c* = 1.0 in $CHCl_3$); **$^1$H NMR** (400 MHz, $C_6D_6$) δ = 7.81 – 7.74 (m, 4H, Ar-H), 7.28 – 7.21 (m, 6H, Ar-H), 4.15 (dd, *J* = 10.2, 3.2 Hz, 1H, -C$H_2$OTBS), 3.82 (td, *J* = 9.9, 5.1 Hz, 1H, 7-$H_a$), 3.70 (ddd, *J* = 10.0, 8.9, 7.4 Hz, 1H, 7-$H_b$), 3.58 (dd, *J* = 10.2, 4.1 Hz, 1H, -C$H_2$OTBS), 3.22 (d, *J* = 4.1 Hz, 1H, 2-H), 3.13 (s, 3H, -$CO_2CH_3$), 2.80 (d, *J* = 4.1 Hz, 1H, 3-H), 2.25 (ddd, *J* = 9.7, 5.9, 3.4 Hz, 1H, 5-H), 2.16 (dt, *J* = 10.5, 3.6 Hz, 1H, 10-H), 2.08 – 1.96 (m, 1H, 6-$H_a$), 1.34 (ddd, *J* = 14.4, 7.3, 3.8 Hz, 1H, 6-$H_b$), 1.29 (s, 3H, -C$H_3$), 1.17 (s, 9H, -SiC(C$H_3$)$_3$), 0.94 (s, 9H, -SiC(C$H_3$)$_3$), 0.06 (s, 3H, -Si(C$H_3$)$_2$), -0.02 (s, 3H, -Si(C$H_3$)$_2$) ppm; **$^{13}$C NMR** (101 MHz, $C_6D_6$) δ = 204.88 (q, 1-C), 172.86 (q, -$CO_2CH_3$), 136.01 (2C), 135.97 (2C), 134.22, 134.10, 130.10 (2C), 128.18 (2C), 128.17 (2C) (Ar-C), 63.95 (s, 7-C), 62.55 (s, -$CH_2$OTBS), 60.91 (t, 3-C), 56.94 (t, 2-C), 54.69 (t, 10-C), 51.49 (p, -$CO_2CH_3$), 46.88 (q, 4-C), 35.00 (s, 6-C), 34.11 (t, 5-C), 27.16 (p, 3C, -SiC($CH_3$)$_3$), 26.06 (p, 3C, -SiC($CH_3$)$_3$), 21.64 (p, -$CH_3$), 19.42 (q, -Si$C$($CH_3$)$_3$), 18.39 (q, -Si$C$($CH_3$)$_3$), -5.32 (p, -Si($CH_3$)$_2$), -5.34 (p, -Si($CH_3$)$_2$) ppm; **HRMS-ESI** ($C_{34}H_{50}O_6Si_2$): calc. for $[M+H]^+$: 611.32187, found: 611.32238.

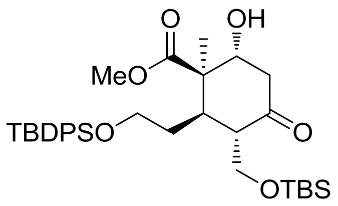


**S30**

**Alcohol S30.** To a solution of $(PhSe)_2$ (1.95 g, 6.26 mmol) in EtOH (70 ml) was added $NaBH_4$ (474 mg, 12.5 mmol) at room temperature. When the solution turned colourless, it was cooled to 0 °C and AcOH (0.12 ml, 2.09 mmol) was added followed by a solution of epoxide **S29** (2.55 g, 4.17 mmol) in EtOH (50 ml). After 10 min at 0 °C, the reaction was diluted with EtOAc (150 ml) and washed with half saturated brine (100 ml). The aqueous phase was extracted with EtOAc (2 × 100 ml) and the combined organic layers were dried ($MgSO_4$) and concentrated in vacuo to give the crude product. Purification by flash chromatography on silica gel with petroleum ether/EtOAc 6:1 as eluent gave alcohol **S30** (2.37 g, 93%) as a colourless oil.

**$R_f$** = 0.45 (petroleum ether:EtOAc = 4:1); $[\alpha]_D^{20}$ = −19.8 (*c* = 1.0 in $CHCl_3$); **$^1$H NMR** (400 MHz, $C_6D_6$) δ = 7.80 – 7.73 (m, 4H, Ar-H), 7.28 – 7.20 (m, 6H, Ar-H), 4.18 (dd, *J* = 9.6, 3.0 Hz, 1H, -C$H_2$OTBS), 3.91 (dt, *J* = 7.7, 3.7 Hz, 1H, 3-H), 3.80 (ddd, *J* = 10.5, 8.1, 4.9 Hz, 1H, 7-$H_a$), 3.71 – 3.60 (m, 2H, 7-$H_b$, -C$H_2$OTBS), 3.15 (s, 3H, -$CO_2CH_3$), 3.03 (d, *J* = 8.0 Hz, 1H, -O*H*), 2.57 (dt, *J* = 8.3, 2.8 Hz, 1H, 10-H), 2.49 – 2.37 (m, 3H, 2-H, 5-H), 1.98 – 1.88 (m, 1H, 6-$H_a$), 1.80 (dtd, *J* = 12.7, 7.5, 5.1 Hz, 1H, 6-$H_b$), 1.39 (s, 3H, -C$H_3$), 1.17 (s, 9H, -SiC(C$H_3$)$_3$), 0.91 (s, 9H, -SiC(C$H_3$)$_3$), 0.08 (s, 3H, -Si(C$H_3$)$_2$), 0.04 (s, 3H, -Si(C$H_3$)$_2$) ppm; **$^{13}$C NMR** (101 MHz, $C_6D_6$) δ = 208.39 (q, 1-C), 175.54 (q, -*C*$O_2CH_3$), 136.00 (4C), 134.24 (2C), 130.10, 130.06, 128.15 (4C) (Ar-C), 72.54 (t, 3-C), 64.53 (s, -*C*$H_2$OTBS), 63.60 (s, 7-C), 55.71 (t, 10-C), 51.58 (q, 4-C), 51.29 (p, -$CO_2$*C*$H_3$), 46.02 (s, 2-C), 36.53 (s, 6-C), 35.87 (t, 5-C), 27.22 (p, 3C, -SiC(*C*$H_3$)$_3$), 26.14 (p, 3C, -SiC(*C*$H_3$)$_3$), 20.96 (p, -*C*$H_3$), 19.46 (q, -Si*C*($CH_3$)$_3$), 18.61 (q, -Si*C*($CH_3$)$_3$), -5.36 (p, -Si(*C*$H_3$)$_2$), -5.41 (p, -Si(*C*$H_3$)$_2$) ppm; **HRMS-ESI** ($C_{34}H_{52}O_6Si_2$): calc. for $[M+H]^+$: 613.33752, found: 613.33758.

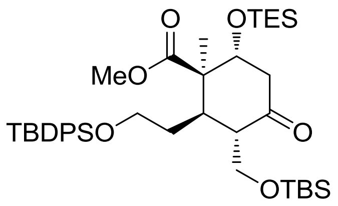

**85**

**Silyl ether 85.** To a solution of alcohol **S30** (1.00 g, 1.63 mmol) in DMF (35 ml) were added imidazole (1.11 g, 16.3 mmol) and TESCl (2.19 ml, 13.1 mmol) at room temperature. The mixture was warmed to 40 °C and stirred for 2 h. The reaction was quenched by the addition of aqueous saturated $NH_4Cl$-solution (30 ml) and the aqueous phase was extracted with EtOAc (3 × 20 ml). The combined organic layers were washed with brine (40 ml), dried ($MgSO_4$) and evaporated under reduced pressure. Purification by flash chromatography on silica gel with petroleum ether/EtOAc 20:1 as eluent gave silyl ether **85** (1.18 g, 99%) as a colourless oil.

$\mathbf{R_f}$ = 0.54 (petroleum ether:EtOAc = 9:1); $[\alpha]_D^{20}$ = −15.1 (*c* = 1.0 in $CHCl_3$); **$^1$H NMR** (400 MHz, $C_6D_6$) δ = 7.85 – 7.78 (m, 4H, Ar-H), 7.28 – 7.20 (m, 6H, Ar-H), 4.25 (dd, *J* = 10.4, 2.2 Hz, 1H, -C$H_2$OTBS), 4.22 (t, *J* = 3.1 Hz, 1H, 3-H), 4.06 – 3.97 (m, 1H, 7-$H_a$), 3.78 (dd, *J* = 17.8, 8.4 Hz, 1H, 7-$H_b$), 3.65 (dd, *J* = 10.4, 3.5 Hz, 1H, -C$H_2$OTBS), 3.25 (s, 3H, -$CO_2$C$H_3$), 2.77 (dt, *J* = 11.5, 2.8 Hz, 1H, 10-H), 2.45 – 2.30 (m, 3H, 2-H, 5-H), 2.20 – 2.10 (m, 2H, 6-H), 1.37 (s, 3H, -C$H_3$), 1.20 (s, 9H, -SiC(C$H_3$)$_3$), 0.97 – 0.89 (m, 18H, -SiC(C$H_3$)$_3$, -Si($CH_2$C$H_3$)$_3$), 0.58 – 0.46 (m, 6H, -Si(C$H_2$$CH_3$)$_3$), 0.17 (s, 3H, -Si(C$H_3$)$_2$), 0.08 (s, 3H, -Si(C$H_3$)$_2$) ppm; **$^{13}$C NMR** (101 MHz, $C_6D_6$) δ = 205.83 (q, 1-C), 175.35 (q, -*C*$O_2$$CH_3$), 136.09 (2C), 136.05 (2C), 134.48, 134.43, 129.97, 129.95, 128.09 (4C) (Ar-C), 75.03 (t, 3-C), 65.19 (s, 7-C), 58.61 (s, -*C*$H_2$OTBS), 55.31 (t, 10-C), 53.01 (q, 4-C), 51.38 (p, -$CO_2$*C*$H_3$), 47.26 (s, 2-C), 36.22 (t, 5-C), 35.88 (s, 6-C), 27.22 (p, 3C, -SiC(*C*$H_3$)$_3$), 26.31 (p, 3C, -SiC(*C*$H_3$)$_3$), 22.06 (p, -*C*$H_3$), 19.46 (q, -Si*C*($CH_3$)$_3$), 18.57 (q, -Si*C*($CH_3$)$_3$), 7.12 (p, 3C, -Si($CH_2$*C*$H_3$)$_3$), 5.16 (s, 3C, -Si(*C*$H_2$$CH_3$)$_3$), -5.09 (p, -Si(*C*$H_3$)$_2$), -5.25 (p, -Si(*C*$H_3$)$_2$) ppm; **HRMS-ESI** ($C_{40}H_{66}O_6Si_3$): calc. for $[M+H]^+$: 727.42400, found: 727.42490.

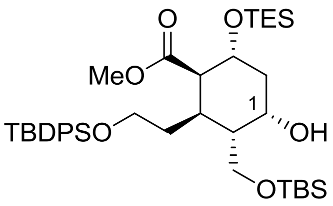

**S31**

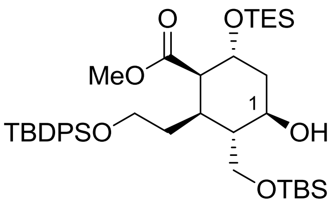

**91**

**Alcohols S31 and 91.** To a solution of silyl ether **85** (1.02 g, 1.40 mmol) in MeOH (50 ml) was added $NaBH_4$ (106 mg, 2.81 mmol) at 0 °C. After stirring for 2 h at 0 °C the mixture was quenched with aqueous saturated $NH_4Cl$-solution (40 ml). The aqueous phase was extracted with EtOAc (3 × 30 ml) and the combined organic layers were dried over $MgSO_4$ and concentrated under reduced pressure. Purification by flash chromatography on silica gel with petroleum ether/EtOAc 20:1 to 4:1 as eluent gave alcohols **S31** (654 mg, 64%) and **91** (331 mg, 32%) as colourless oils.

**S31:** $\mathbf{R_f}$ = 0.63 (petroleum ether:EtOAc = 9:1); $[\alpha]_D^{20}$ = +1.9 (*c* = 1.0 in $CHCl_3$); $^1$**H NMR** (400 MHz, $C_6D_6$) δ = 7.86 – 7.78 (m, 4H, Ar-H), 7.32 – 7.20 (m, 6H, Ar-H), 4.47 – 4.39 (m, 1H, 1-H), 4.31 (s, 1H, 3-H), 4.08 (t, *J* = 9.3 Hz, 1H, -C$H_2$OTBS), 3.96 (dd, *J* = 9.1, 4.4 Hz, 1H, -C$H_2$OTBS), 3.83 – 3.74 (m, 1H, 7-$H_a$), 3.71 (d, *J* = 10.0 Hz, 1H, -O*H*), 3.69 – 3.61 (m, 1H, 7-$H_b$), 3.24 (s, 3H, -$CO_2CH_3$), 2.42 – 2.32 (m, 1H, 10-H), 2.20 – 2.02 (m, 4H, 2-$H_a$, 5-H, 6-H), 1.71 – 1.63 (m, 1H, 2-$H_b$), 1.37 (s, 3H, -C$H_3$), 1.20 (s, 9H, -SiC(C$H_3$)$_3$), 1.02 (s, 9H, -SiC(C$H_3$)$_3$), 0.91 (t, *J* = 7.9 Hz, 9H, -Si(CH$_2$C$H_3$)$_3$), 0.63 – 0.46 (m, 6H, -Si(C$H_2$CH$_3$)$_3$), 0.19 (s, 3H, -Si(C$H_3$)$_2$), 0.14 (s, 3H, -Si(C$H_3$)$_2$) ppm; $^{13}$**C NMR** (101 MHz, $C_6D_6$) δ = 175.32 (q, -*C*O$_2$CH$_3$), 136.10 (2C), 136.05 (2C), 134.19, 134.15, 129.99 (2C), 128.15 (2C), 128.14 (2C) (Ar-C), 75.64 (t, 3-C), 66.86 (t, 1-C), 65.23 (s, 7-C), 63.45 (s, -*C*H$_2$OTBS), 53.02 (q, 4-C), 51.18 (p, -CO$_2$*C*H$_3$), 47.93 (t, 10-C), 35.87 (s, 2-C), 33.68 (s, 6-C), 32.65 (t, 5-C), 27.23 (p, 3C, -SiC(*C*H$_3$)$_3$), 26.33 (p, 3C, -SiC(*C*H$_3$)$_3$), 23.03 (p, -*C*H$_3$), 19.51 (q, -Si*C*(CH$_3$)$_3$), 18.68 (q, -Si*C*(CH$_3$)$_3$), 7.10 (p, 3C, -Si(CH$_2$*C*H$_3$)$_3$), 5.11 (s, 3C, -Si(*C*H$_2$CH$_3$)$_3$), -5.07 (p, -Si(*C*H$_3$)$_2$), -5.11 (p, -Si(*C*H$_3$)$_2$) ppm; **HRMS-ESI** ($C_{40}H_{68}O_6Si_3$): calc. for $[M+H]^+$: 729.43965, found: 729.44007.

**91:** $\mathbf{R_f}$ = 0.21 (petroleum ether:EtOAc = 9:1); $[\alpha]_D^{20}$ = −12.5 (*c* = 1.0 in $CHCl_3$); $^1$**H NMR** (400 MHz, $C_6D_6$) δ = 7.84 – 7.78 (m, 4H, Ar-H), 7.29 – 7.20 (m, 6H, Ar-H), 4.25 (s, 1H, 3-H), 4.21 (dd, *J* = 10.4, 4.2 Hz, 1H, 1-H), 4.16 (dd, *J* = 10.2, 2.8 Hz, 1H, -C$H_2$OTBS), 3.92 – 3.83 (m, 1H, 7-$H_a$), 3.74 – 3.61 (m, 2H, 7-$H_b$, -C$H_2$OTBS), 3.28 (s, 3H, -$CO_2CH_3$), 2.83 (bs, 1H, -O*H*), 2.24 (tdd, *J* = 11.8, 6.3, 2.9 Hz, 1H, 10-H), 2.15 (dt, *J* = 7.2, 4.4 Hz, 3H, 2-$H_a$,

6-H), 1.75 (dt, $J$ = 11.7, 3.8 Hz, 1H, 5-H), 1.71 – 1.61 (m, 1H, 2-$H_b$), 1.34 (s, 3H, -C$H_3$), 1.19 (s, 9H, -SiC(C$H_3$)$_3$), 0.96 (t, $J$ = 7.9 Hz, 9H, -Si(CH$_2$C$H_3$)$_3$), 0.90 (s, 9H, -SiC(C$H_3$)$_3$), 0.62 – 0.50 (m, 6H, -Si(C$H_2$CH$_3$)$_3$), 0.04 (s, 3H, -Si(C$H_3$)$_2$), -0.02 (s, 3H, -Si(C$H_3$)$_2$) ppm; **$^{13}$C NMR** (101 MHz, $C_6D_6$) δ = 175.47 (q, -$C$O$_2$CH$_3$), 136.07 (2C), 136.04 (2C), 134.39 (2C), 129.97, 129.96, 128.12 (4C) (Ar-C), 74.14 (t, 3-C), 68.40 (t, 1-C), 65.67 (s, 7-C), 64.80 (s, -$C$H$_2$OTBS), 53.08 (q, 4-C), 51.17 (p, -CO$_2$$C$H$_3$), 49.28 (t, 10-C), 39.87 (s, 6-C), 35.26 (s, 2-C), 34.80 (t, 5-C), 27.21 (p, 3C, -SiC($C$H$_3$)$_3$), 26.13 (p, 3C, -SiC($C$H$_3$)$_3$), 22.62 (p, -$C$H$_3$), 19.48 (q, -Si$C$(CH$_3$)$_3$), 18.37 (q, -Si$C$(CH$_3$)$_3$), 7.26 (p, 3C, -Si(CH$_2$$C$H$_3$)$_3$), 5.32 (s, 3C, -Si($C$H$_2$CH$_3$)$_3$), -5.41 (p, -Si($C$H$_3$)$_2$), -5.43 (p, -Si($C$H$_3$)$_2$) ppm; **HRMS-ESI** ($C_{40}H_{68}O_6Si_3$): calc. for $[M+H]^+$: 729.43965, found: 729.43996.

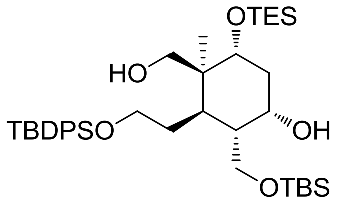


**S32**

**Diol S32.** To a solution of alcohol **S31** (338 mg, 0.46 mmol) in THF (25 ml) was added $LiBH_4$ (4 M in THF, 0.46 ml, 1.85 mmol) at room temperature. The resulting solution was heated to 65 °C and stirred for 2 d. Additional $LiBH_4$ (4 M in THF, 0.23 ml, 0.93 mmol) was added and the reaction mixture was stirred for another 2 d at 65 °C. After cooling to room temperature aqueous saturated $NH_4Cl$-solution (20 ml) and water (10 ml) were added and the layers were separated. The aqueous phase was extracted with EtOAc (3 × 50 ml). The combined organic layers were washed with brine (75 ml), dried ($MgSO_4$) and evaporated under reduced pressure. Purification by flash chromatography on silica gel with petroleum ether/EtOAc 20:1 to 4:1 as eluent gave diol **S32** (217 mg, 67%) as a colourless solid and starting material **S31** (93 mg, 28%).

**$R_f$** = 0.64 (petroleum ether:EtOAc = 4:1); $[\alpha]_D^{20}$ = +1.3 ($c$ = 1.0 in $CHCl_3$); **$^1$H NMR** (400 MHz, $C_6D_6$) δ = 7.88 – 7.77 (m, 4H, Ar-H), 7.34 – 7.20 (m, 6H, Ar-H), 4.36 – 4.27 (m, 1H, 1-H), 4.12 (s, 1H, 3-H), 4.02 (t, $J$ = 9.1 Hz, 1H, -C$H_2$OTBS), 3.95 (d, $J$ = 9.8 Hz, 1H, -O$H$), 3.80 (dd, $J$ = 9.1, 4.4 Hz, 1H, -C$H_2$OTBS), 3.78 – 3.70 (m, 1H, 7-$H_a$), 3.68 – 3.58 (m, 1H, 7-$H_b$), 3.29 (d, $J$ = 10.7 Hz, 1H, 29-$H_a$), 3.14 (d, $J$ = 10.7 Hz, 1H, 29-$H_b$), 2.03 (dt, $J$ = 14.6, 3.1 Hz, 1H, 2-$H_a$), 1.95 (ddd, $J$ = 12.1, 6.2, 1.9 Hz, 1H, 5-H), 1.89 – 1.77 (m, 1H, 6-$H_a$),

1.74 – 1.62 (m, 2H, 2-$H_b$, 10-H), 1.50 (dt, *J* = 11.7, 6.5 Hz, 1H, 6-$H_b$), 1.21 (s, 9H, -SiC(C$H_3$)$_3$), 1.06 (s, 3H, -C$H_3$), 1.03 (s, 9H, -SiC(C$H_3$)$_3$), 0.94 (t, *J* = 7.9 Hz, 9H, -Si(C$H_2$C$H_3$)$_3$), 0.63 – 0.51 (m, 6H, -Si(C$H_2$C$H_3$)$_3$), 0.19 (s, 3H, -Si(C$H_3$)$_2$), 0.13 (s, 3H, -Si(C$H_3$)$_2$) ppm; **$^{13}$C NMR** (101 MHz, $C_6D_6$) δ = 136.11 (2C), 136.05 (2C), 134.24, 134.20, 130.03 (2C), 128.15 (4C) (Ar-C), 74.50 (t, 3-C), 66.86 (t, 1-C), 65.12 (s, 7-C), 64.88 (s, 29-C), 63.71 (s, -*C*$H_2$OTBS), 47.05 (t, 10-C), 43.70 (q, 4-C), 34.21 (s, 2-C), 32.51 (t, 5-C), 31.55 (s, 6-C), 27.21 (p, 3C, -SiC(*C*$H_3$)$_3$), 26.33 (p, 3C, -SiC(*C*$H_3$)$_3$), 20.97 (p, -*C*$H_3$), 19.51 (q, -Si*C*(C$H_3$)$_3$), 18.67 (q, -Si*C*(C$H_3$)$_3$), 7.19 (p, 3C, -Si(C$H_2$*C*$H_3$)$_3$), 5.22 (s, 3C, -Si(*C*$H_2$C$H_3$)$_3$), -5.12 (p, 2C, -Si(*C*$H_3$)$_2$) ppm; **HRMS-ESI** ($C_{39}H_{68}O_5Si_3$): calc. for $[M+H]^+$: 701.44473, found: 701.44518; **m.p.:** 93 °C.

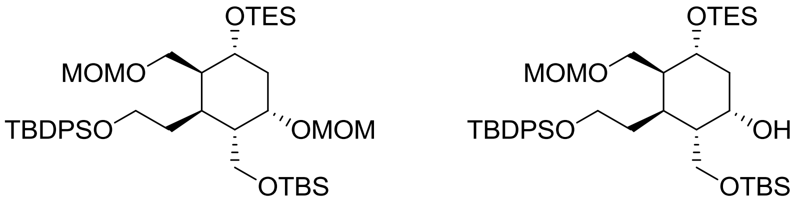


**86** **S33**

***Bis*-MOM ether 86 and MOM ether S33.** To a solution of diol **S32** (705 mg, 1.01 mmol) in THF (50 ml) were added DIPEA (5.25 ml, 30.1 mmol), MOMCl (1.53 ml, 20.1 mmol) and NaI (754 mg, 5.03 mmol). The mixture was stirred at 50 °C in a sealed tube for 4 d. The reaction was quenched by the addition of water (100 ml) and EtOAc (100 ml). The resulting layers were separated and the aqueous phase was extracted with EtOAc (2 × 100 ml). The combined organic layers were washed with brine (130 ml), dried over $MgSO_4$ and concentrated under reduced pressure. Purification by flash chromatography on silica gel with petroleum ether/EtOAc 30:1 as eluent gave *bis*-MOM ether **86** (558 mg, 70%) and MOM-ether **S33** (160 mg, 21%) as colourless oils.

**86:** **$R_f$** = 0.38 (petroleum ether:EtOAc = 20:1); $[\alpha]_D^{20}$ = +1.8 (*c* = 1.0 in $CHCl_3$); **$^1$H NMR** (400 MHz, $C_6D_6$) δ = 7.89 – 7.78 (m, 4H, Ar-H), 7.32 – 7.20 (m, 6H, Ar-H), 4.68 (s, 2H, -OC$H_2$OC$H_3$), 4.42 (s, *J* = 6.5 Hz, 2H, -OC$H_2$OC$H_3$), 4.16 – 4.08 (m, 1H, 1-H), 4.05 (dd, *J* = 9.9, 7.1 Hz, 1H, -C$H_2$OTBS), 3.95 – 3.80 (m, 4H, 3-H, 7-H, -C$H_2$OTBS), 3.58 (d, *J* = 9.4 Hz, 1H, 29-$H_a$), 3.48 (d, *J* = 9.4 Hz, 1H, 29-$H_b$), 3.27 (s, 3H, -OC$H_2$OC$H_3$), 3.20 (s, 3H, -OC$H_2$OC$H_3$), 2.36 – 2.27 (m, 1H, 5-H), 2.20 – 2.00 (m, 3H, 2-$H_a$, 6-$H_a$, 10-H), 1.91 (dt, *J* = 13.4, 4.0 Hz, 1H, 2-$H_b$), 1.66 (dtd, *J* = 13.9, 8.4, 5.5 Hz, 1H, 6-$H_b$), 1.28 (s, 3H, -C$H_3$),

1.21 (s, 9H, -SiC(C*H*$_3$)$_3$), 1.01 (t, *J* = 7.9 Hz, 9H, -Si(CH$_2$C*H*$_3$)$_3$), 0.95 (s, 9H, -SiC(C*H*$_3$)$_3$), 0.68 – 0.56 (m, 6H, -Si(C*H*$_2$CH$_3$)$_3$), 0.06 (s, 3H, -Si(C*H*$_3$)$_2$), 0.04 (s, 3H, -Si(C*H*$_3$)$_2$) ppm; **$^{13}$C NMR** (101 MHz, $C_6D_6$) δ = 136.10 (2C), 136.06 (2C), 134.48 (2C), 129.93 (2C), 128.10 (4C) (Ar-C), 97.17 (s, -O*C*H$_2$OCH$_3$), 95.22 (s, -O*C*H$_2$OCH$_3$), 73.76 (s, 29-C), 71.53 (2C, 1-C, 3-C), 65.09 (s, 7-C), 63.37 (s, -*C*H$_2$OTBS), 55.34 (p, -OCH$_2$O*C*H$_3$), 55.23 (p, -OCH$_2$O*C*H$_3$), 45.05 (t, 10-C), 42.92 (q, 4-C), 35.96 (t, 5-C), 33.93 (s, 2-C), 32.51 (s, 6-C), 27.24 (p, 3C, -SiC(*C*H$_3$)$_3$), 26.26 (p, 3C, -SiC(*C*H$_3$)$_3$), 20.22 (p, -*C*H$_3$), 19.53 (q, -Si*C*(CH$_3$)$_3$), 18.50 (q, -Si*C*(CH$_3$)$_3$), 7.34 (p, 3C, -Si(CH$_2$*C*H$_3$)$_3$), 5.58 (s, 3C, -Si(*C*H$_2$CH$_3$)$_3$), -5.13 (p, -Si(*C*H$_3$)$_2$), -5.19 (p, -Si(*C*H$_3$)$_2$) ppm; **HRMS-ESI** ($C_{43}H_{76}O_7Si_3$): calc. for $[M+H]^+$: 789.49716, found: 789.49819.

**S33: R$_f$** = 0.18 (petroleum ether:EtOAc = 20:1); $[\alpha]_D^{20}$ = +3.8 (*c* = 1.0 in $CHCl_3$); **$^1$H NMR** (400 MHz, $C_6D_6$) δ = 7.88 – 7.79 (m, 4H, Ar-H), 7.34 – 7.21 (m, 6H, Ar-H), 4.38 (m, 3H, 1 H, -OC*H*$_2$OCH$_3$), 4.19 (s, 1H, 3-H), 4.03 (t, *J* = 9.1 Hz, 1H, -C*H*$_2$OTBS), 3.96 (d, *J* = 10.0 Hz, 1H, -O*H*), 3.82 (dd, *J* = 9.2, 4.4 Hz, 1H, -C*H*$_2$OTBS), 3.79 – 3.71 (m, 1H, 7-H$_a$), 3.69 – 3.60 (m, 1H, 7-H$_b$), 3.44 (d, *J* = 9.7 Hz, 1H, 29-H$_a$), 3.30 (d, *J* = 9.7 Hz, 1H, 29-H$_b$), 3.12 (s, 3H, -OCH$_2$OC*H*$_3$), 2.13 – 1.99 (m, 2H, 2-H$_a$, 5-H), 1.96 – 1.83 (m, 1H, 6-H$_a$), 1.82 – 1.69 (m, 2H, 2-H$_b$, 10-H), 1.57 – 1.44 (m, 1H, 6-H$_b$), 1.21 (s, 9H, -SiC(C*H*$_3$)$_3$), 1.21 (s, 3H, -C*H*$_3$), 1.03 (s, 9H, -SiC(C*H*$_3$)$_3$), 0.95 (t, *J* = 7.9 Hz, 9H, -Si(CH$_2$C*H*$_3$)$_3$), 0.68 – 0.50 (m, 6H, -Si(C*H*$_2$CH$_3$)$_3$), 0.20 (s, 3H, -Si(C*H*$_3$)$_2$), 0.14 (s, 3H, -Si(C*H*$_3$)$_2$) ppm; **$^{13}$C NMR** (101 MHz, $C_6D_6$) δ = 136.13 (2C), 136.06 (2C), 134.23, 134.20, 130.02 (2C), 128.15 (4C) (Ar-C), 96.92 (s, -O*C*H$_2$OCH$_3$), 74.98 (t, 3-C), 70.60 (s, 29-C), 66.80 (t, 1-C), 65.11 (s, 7-C), 63.64 (s, -*C*H$_2$OTBS), 55.03 (p, -OCH$_2$O*C*H$_3$), 47.14 (t, 10-C), 43.14 (q, 4-C), 34.17 (s, 2-C), 32.43 (t, 5-C), 31.54 (s, 6-C), 27.22 (p, 3C, -SiC(*C*H$_3$)$_3$), 26.33 (p, 3C, -SiC(*C*H$_3$)$_3$), 21.86 (p, -*C*H$_3$), 19.51 (q, -Si*C*(CH$_3$)$_3$), 18.68 (q, -Si*C*(CH$_3$)$_3$), 7.18 (p, 3C, -Si(CH$_2$*C*H$_3$)$_3$), 5.23 (s, 3C, -Si(*C*H$_2$CH$_3$)$_3$), -5.13 (p, 2C, -Si(*C*H$_3$)$_2$) ppm; **HRMS-ESI** ($C_{41}H_{72}O_6Si_3$): calc. for $[M+H]^+$: 745.47095, found: 745.47186.

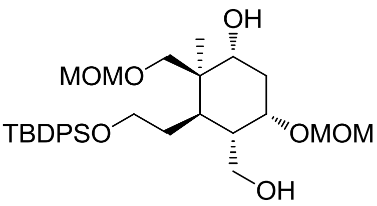


**S34**

**Diol S34**. *Bis*-MOM ether **86** (700 mg, 0.89 mmol) was stirred in a solution of 5% TFA in $CH_2Cl_2$ (7.5 ml) at room temperature. After 30 min the reaction was quenched by the addition of aqueous saturated $NaHCO_3$-solution (25 ml). The aqueous phase was extracted with $CH_2Cl_2$ (2 × 40 ml). The combined organic layers were washed with brine (50 ml), dried ($MgSO_4$) and concentrated in vacuo. Purification by flash chromatography on silica gel with petroleum ether/EtOAc 1:1 as eluent gave diol **S34** (369 mg, 74%) as a colourless oil.

$\mathbf{R}_f$ = 0.25 (petroleum ether:EtOAc = 1:1); $[\alpha]_D^{20}$ = +29.8 (*c* = 1.0 in $CHCl_3$); $^{1}$**H NMR** (400 MHz, $C_6D_6$) δ = 7.88 – 7.80 (m, 4H, Ar-H), 7.31 – 7.20 (m, 6H, Ar-H), 4.36 (s, 2H, -OC*H*$_2$OCH$_3$), 4.30 (d, *J* = 6.6 Hz, 1H, -OC*H*$_2$OCH$_3$), 4.16 (d, *J* = 6.6 Hz, 1H, -OC*H*$_2$OCH$_3$), 3.98 (s, 1H, 3-H), 3.95 – 3.90 (m, 1H, 1-H), 3.82 (td, *J* = 9.6, 5.2 Hz, 1H, 7-$H_a$), 3.75 – 3.66 (m, 1H, 7-$H_b$), 3.53 – 3.39 (m, 2H, -C*H*$_2$OH), 3.33 (d, *J* = 9.7 Hz, 1H, 29-$H_a$), 3.22 (d, *J* = 9.8 Hz, 1H, 29-H*b*), 3.11 (s, 3H, -OCH$_2$OC*H*$_3$), 3.01 (s, 3H, -OCH$_2$OC*H*$_3$), 2.03 – 1.88 (m, 2H, 2-$H_a$, 6-$H_a$), 1.74 (ddd, *J* = 8.5, 6.0, 2.0 Hz, 1H, 5-H), 1.60 (dt, *J* = 14.9, 2.9 Hz, 1H, 2-$H_b$), 1.47 (ddd, *J* = 15.6, 8.0, 3.7 Hz, 1H, 10-H), 1.39 – 1.32 (m, 1H, 6-$H_b$), 1.31 (s, 3H, -C*H*$_3$), 1.22 (s, 9H, -SiC(C*H*$_3$)$_3$) ppm; $^{13}$**C NMR** (101 MHz, $C_6D_6$) δ = 136.17 (2C), 136.15 (2C), 134.40 (2C), 130.01 (2C), 128.13 (4C) (Ar-C), 96.90 (s, -O*C*H$_2$OCH$_3$), 96.12 (s, -O*C*H$_2$OCH$_3$), 75.96 (t, 1-C), 72.54 (t, 3-C), 71.29 (s, 29-C), 65.13 (s, 7-C), 62.55 (s, -*C*H$_2$OH), 55.97 (p, -OCH$_2$O*C*H$_3$), 55.05 (p, -OCH$_2$O*C*H$_3$), 44.52 (t, 10-C), 42.70 (q, 4-C), 32.46 (t, 5-C), 31.84 (s, 6-C), 31.16 (s, 2-C), 27.23 (p, 3C, -SiC(*C*H$_3$)$_3$), 21.35 (p, -*C*H$_3$), 19.49 (q, -Si*C*(CH$_3$)$_3$) ppm; **HRMS-ESI** ($C_{31}H_{48}O_7Si$): calc. for $[M+H]^+$: 561.32421, found: 561.32482.

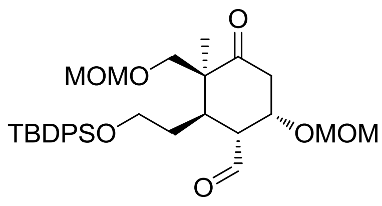


**S35**

**Aldehyde S35.** To a solution of diol **S34** (145 mg, 0.26 mmol) and $NaHCO_3$ in $CH_2Cl_2$ (25 ml) was added Dess–Martin periodinane (439 mg, 1.03 mmol) at 0 °C. The reaction mixture was stirred for 3 h at 0 °C. Aqueous $Na_2S_2O_3$-solution (35 ml) was added and the aqueous phase was extracted with $CH_2Cl_2$ (2 × 30 ml). The combined organic layers were washed with brine (35 ml), dried ($MgSO_4$) and concentrated under reduced pressure. Purification by flash chromatography on silica gel with petroleum ether/EtOAc 4:1 as eluent gave aldehyde **S35** (110 mg, 76%) as a colourless oil.

$\mathbf{R}_f$ = 0.32 (petroleum ether:EtOAc = 2:1); $[\alpha]_D^{20}$ = +4.6 (*c* = 1.0 in $CHCl_3$); **$^1$H NMR** (400 MHz, $C_6D_6$) δ = 9.76 (d, *J* = 3.0 Hz, 1H, -C*H*O),7.81 – 7.73 (m, 4H, Ar-H), 7.29 – 7.22 (m, 6H, Ar-H), 4.41 (d, *J* = 7.1 Hz, 1H, -OC$H_2$OC$H_3$), 4.24 (d, *J* = 7.1 Hz, 1H, -OC$H_2$OC$H_3$), 4.19 – 4.11 (m, 3H, 1-H, -OC$H_2$OC$H_3$), 3.77 – 3.68 (m, 1H, 7-$H_a$), 3.65 – 3.57 (m, 1H, 7-$H_b$), 3.40 (d, *J* = 9.7 Hz, 1H, 29-$H_a$), 3.12 (d, *J* = 9.8 Hz, 1H, 29-$H_b$), 3.08 (s, 3H, -OC$H_2$OC$H_3$), 3.00 (s, 3H, -OC$H_2$OC$H_3$), 2.73 – 2.63 (m, 2H, 2-$H_a$, 5-H), 2.56 (dt, *J* = 12.2, 2.8 Hz, 1H, 10-H), 2.44 (dd, *J* = 14.2, 2.9 Hz, 1H, 2-$H_b$), 1.74 (dtd, *J* = 11.4, 7.3, 4.4 Hz, 1H, 6-$H_a$), 1.48 – 1.38 (m, 1H, 6-$H_b$), 1.16 (s, 9H, -SiC(C$H_3$)$_3$), 1.12 (s, 3H, -C$H_3$) ppm; **$^{13}$C NMR** (101 MHz, $C_6D_6$) δ = 208.42 (q, 6-C), 203.69 (t, -*C*HO), 136.05 (4C), 134.00, 133.93, 130.15 (2C), 128.20 (4C) (Ar-C), 96.59 (s, -O*C*$H_2$OC$H_3$), 95.32 (s, -O*C*$H_2$OC$H_3$), 75.16 (t, 1-C), 71.61 (s, 29-C), 63.45 (s, 7-C), 56.10 (t, 10-C), 55.74 (p, -OC$H_2$O*C*$H_3$), 55.36 (p, -OC$H_2$O*C*$H_3$), 51.97 (q, 4-C), 43.74 (s, 2-C), 37.46 (t, 5-C), 32.69 (s, 6-C), 27.13 (p, 3C, -SiC(*C*$H_3$)$_3$), 19.42 (p, -*C*$H_3$), 19.11 (q, -Si*C*(C$H_3$)$_3$) ppm; **HRMS-ESI** ($C_{31}H_{44}O_7Si$): calc. for $[M+H]^+$: 557.29291, found: 557.29306.

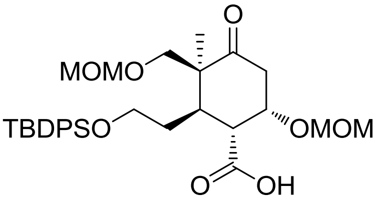


**87**

**Carboxylic acid 87.** To a solution of aldehyde **S35** (110 mg, 0.20 mmol) in *t*-BuOH (12 ml) and water (3 ml) were added 2-methyl-2-butene (0.42 ml, 3.95 mmol), $NaH_2PO_4$ (94.8 mg, 0.79 mmol) and $NaClO_2$ (80%, 112 mg, 0.99 mmol) at 0 °C. After 10 min, the mixture was allowed to warm to room temperature and stirred for 3.5 h. The reaction was quenched by the addition of aqueous saturated $NH_4Cl$-solution (15 ml). The aqueous phase was extracted with EtOAc (3 × 15 ml) and the combined organic layers were washed with brine (30 ml), dried ($MgSO_4$) and concentrated under reduced pressure. Purification by flash chromatography on silica gel (petroleum ether/EtOAc 2:1, 0.1% acetic acid) gave carboxylic acid **87** (103 mg, 91%) as a colourless oil.

$\mathbf{R_f}$ = 0.19 (petroleum ether:EtOAc = 1:1); $[\boldsymbol{\alpha}]_{\mathbf{D}}^{\mathbf{20}}$ = −6.0 (*c* = 1.0 in $CHCl_3$); **$^{1}$H NMR** (400 MHz, $C_6D_6$) δ = 7.85 – 7.77 (m, 4H, Ar-H), 7.31 – 7.21 (m, 6H, Ar-H), 4.50 (d, *J* = 7.1 Hz, 1H, -OC$H_2$OCH$_3$), 4.42 – 4.35 (m, 2H, 1-H, -OC$H_2$OCH$_3$), 4.18 – 4.06 (m, 3H, 7-H$_a$, -OC$H_2$OCH$_3$), 3.81 (td, *J* = 9.8, 6.5 Hz, 1H, 7-H$_b$), 3.40 (d, *J* = 9.8 Hz, 1H, 29-H$_a$), 3.20 (s, 3H, -OCH$_2$OC$H_3$), 3.15 – 3.08 (m, 2H, 10-H, 29-H$_b$), 3.01 (s, 3H, -OCH$_2$OC$H_3$), 2.75 (dd, *J* = 14.2, 3.8 Hz, 1H, 2-H$_a$), 2.57 (dd, *J* = 14.1, 2.9 Hz, 1H, 2-H$_b$), 2.46 (dt, *J* = 12.1, 4.5 Hz, 1H, 5-H), 1.98 – 1.84 (m, 1H, 6-H$_a$), 1.74 – 1.62 (m, 1H, 6-H$_b$), 1.19 (s, 9H, -SiC(C$H_3$)$_3$), 1.01 (s, 3H, -C$H_3$) ppm; **$^{13}$C NMR** (101 MHz, $C_6D_6$) δ = 208.75 (q, 3-C), 177.33 (q, -*C*O$_2$H), 136.13 (2C), 136.06 (2C), 134.36, 134.28, 130.07, 130.05, 128.17 (2C), 128.16 (2C) (Ar-C), 96.57 (s, -O*C*H$_2$OCH$_3$), 95.53 (s, -O*C*H$_2$OCH$_3$), 75.68 (t, 1-C), 71.85 (s, 29-C), 64.41 (s, 7-C), 55.72 (p, -OCH$_2$O*C*H$_3$), 55.40 (p, -OCH$_2$O*C*H$_3$), 51.75 (q, 4-C), 50.78 (t, 10-C), 44.07 (s, 2-C), 38.42 (t, 5-C), 33.87 (s, 6-C), 27.22 (p, 3C, -SiC(*C*H$_3$)$_3$), 19.46 (p, -*C*H$_3$), 19.39 (q, -Si*C*(CH$_3$)$_3$) ppm; **HRMS-ESI** ($C_{31}H_{44}O_8Si$): calc. for $[M+Na]^+$: 595.26977, found: 579.26878.

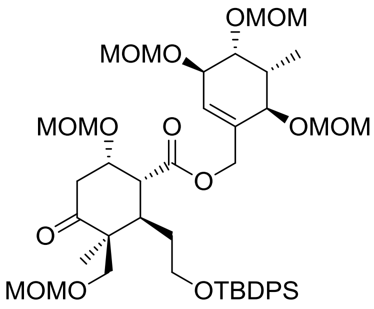

**88**

**Ester 88**. To a solution of carboxylic acid **87** (27 mg, 47.1 µmol) in $CH_2Cl_2$ (0.5 ml) was added EDC·HCl (20 mg, 0.10 mmol) and DMAP (6 mg, 51.9 µmol). After stirring for 30 min a solution of alcohol **40** (14 mg, 47.1 µmol) in $CH_2Cl_2$ (0.5 ml) was added. The reaction mixture was stirred at room temperature for 2 d. EtOAc (10 ml) was added and the organic layer was washed with 1 M HCl (10 ml), aqueous saturated $NaHCO_3$-solution (10 ml), water (10 ml) and brine (10 ml), dried ($MgSO_4$) and concentrated under reduced pressure. Purification by flash chromatography on silica gel with petroleum ether/EtOAc 4:1 to 2:1 as eluent gave ester **91** (21 mg, 52%) as a colourless oil.

$\mathbf{R}_f$ = 0.29 (petroleum ether:EtOAc = 1:1); $[\alpha]_D^{20}$ = −3.8 (*c* = 1.0 in $CHCl_3$); **$^1$H NMR** (400 MHz, $CDCl_3$) δ = 7.69 – 7.62 (m, 4H, Ar-H), 7.45 – 7.31 (m, 6H, Ar-H), 5.86 – 5.81 (m, 1H, 9-H), 4.83 – 4.64 (m, 6H, -OC$H_2$OC$H_3$), 4.59 – 4.52 (m, 3H, 1'-H, -OC$H_2$OC$H_3$), 4.51 – 4.45 (m, 3H, -OC$H_2$OC$H_3$), 4.34 (dd, *J* = 6.5, 3.2 Hz, 1H, 1-H), 4.08 (d, *J* = 7.5 Hz, 1H, 11-H), 3.96 (dd, *J* = 7.7, 3.9 Hz, 1H, 12-H), 3.86 (td, *J* = 10.0, 5.5 Hz, 1H, 7-$H_a$), 3.80 (d, *J* = 3.1 Hz, 1H, 14-H), 3.66 – 3.55 (m, 2H, 7-$H_b$, 29-$H_a$), 3.42 – 3.35 (m, 9H, -OC$H_2$OC$H_3$), 3.29 (s, 3H, -OC$H_2$OC$H_3$), 3.29 – 3.26 (m, 1H, 29-$H_b$), 3.26 (s, 3H, -OC$H_2$OC$H_3$), 3.15 (dd, *J* = 12.0, 2.9 Hz, 1H, 10-H), 2.76 (qd, *J* = 14.3, 3.7 Hz, 2H, 2-H), 2.44 (ddq, *J* = 10.8, 7.3, 3.6 Hz, 1H, 13-H), 2.22 (dt, *J* = 11.9, 4.7 Hz, 1H, 5-H), 1.82 (ddd, *J* = 15.2, 10.7, 5.6 Hz, 1H, 6-$H_a$), 1.49 (ddd, *J* = 14.4, 10.0, 5.1 Hz, 1H, 6-$H_b$), 1.03 (s, 9H, -SiC(C$H_3$)$_3$), 0.96 (d, *J* = 7.2 Hz, 3H, 18-H), 0.88 (s, 3H, 28-H) ppm; **$^{13}$C NMR** (101 MHz, $CDCl_3$) δ = 210.61 (q, 3-C), 171.84 (q, -*C*(O)OR), 135.75 (2C), 135.70 (2C), 134.09, 133.97 (Ar-C), 133.48 (q, 8-C), 129.78, 129.74 (Ar-C), 128.92 (t, 9-C), 127.80 (4C) (Ar-C), 97.26 (s, -O*C*$H_2$OC$H_3$), 96.83 (s, -O*C*$H_2$OC$H_3$), 96.78 (s, -O*C*$H_2$OC$H_3$), 96.54 (s, -O*C*$H_2$OC$H_3$), 95.61 (s, -O*C*$H_2$OC$H_3$), 77.78 (t, 14-C), 77.09 (t, 12-C), 75.15 (t, 1-C), 73.96 (t, 11-C), 71.87 (s, 29-C), 65.16 (s, 1'-C), 64.06 (s, 7-C), 56.05 (p, -OC$H_2$O*C*$H_3$), 55.88 (p, -OC$H_2$O*C*$H_3$), 55.85 (p, -OC$H_2$O*C*$H_3$), 55.59 (p, 2C, -OC$H_2$O*C*$H_3$), 51.66 (q, 4-C), 50.67 (t, 10-C), 44.45 (s, 2-C), 38.47 (t, 5-C), 36.51 (t, 13-C), 33.69 (s, 6-C), 27.04 (p, 3C, -SiC(*C*$H_3$)$_3$), 19.28 (q, -Si*C*(C$H_3$)$_3$), 19.06 (p, 28-C),

11.47 (p, 18-C) ppm; **HRMS-ESI** ($C_{45}H_{68}O_{14}Si$): calc. for $[M+H]^+$: 861.44511, found: 861.44645.

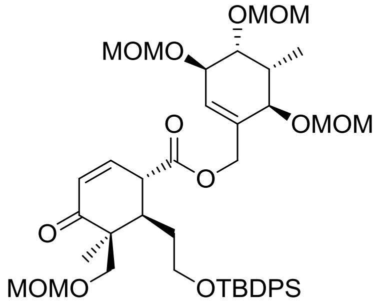


**90**

**Enone 90.** $\mathbf{R}_f$ = 0.29 (petroleum ether:EtOAc = 2:1); $[\alpha]_D^{20}$ = +52.9 (*c* = 1.0 in $CHCl_3$); **$^1$H NMR** (400 MHz, $CDCl_3$) δ = 7.67 – 7.57 (m, 4H, Ar-H), 7.47 – 7.32 (m, 6H, Ar-H), 6.73 (dd, *J* = 10.1, 3.4 Hz, 1H, 1-H), 6.02 (dd, *J* = 10.2, 2.0 Hz, 1H, 2-H), 5.84 – 5.75 (m, 1H, 9-H), 4.79 (d, *J* = 6.7 Hz, 1H, $-OCH_2OCH_3$), 4.77 – 4.60 (m, 6H, 30-$H_a$, $-OCH_2OCH_3$), 4.54 – 4.45 (m, 3H, 30-$H_b$, $-OCH_2OCH_3$), 4.07 (d, *J* = 7.7 Hz, 1H, 11-H), 3.94 (dd, *J* = 7.8, 3.9 Hz, 1H, 12-H), 3.79 (d, *J* = 3.1 Hz, 1H, 14-H), 3.72 – 3.57 (m, 4H, 7-H, 10-H, 29-$H_a$), 3.53 (d, *J* = 9.9 Hz, 1H, 29-$H_b$), 3.39 (s, 3H, $-OCH_2OCH_3$), 3.38 (s, 3H, $-OCH_2OCH_3$), 3.37 (s, 3H, $-OCH_2OCH_3$), 3.27 (s, 3H, $-OCH_2OCH_3$), 2.65 – 2.56 (m, 1H, 5-H), 2.47 – 2.37 (m, 1H, 13-H), 1.92 (dtd, *J* = 14.4, 7.5, 3.6 Hz, 1H, 6-$H_a$), 1.53 (dt, *J* = 21.1, 7.2 Hz, 1H, 6-$H_b$), 1.12 (s, 3H, 28-H), 1.03 (s, 9H, $-SiC(CH_3)_3$), 0.93 (d, *J* = 7.2 Hz, 3H, 18-H) ppm; **$^{13}$C NMR** (101 MHz, $CDCl_3$) δ = 201.85 (q, 3-C), 172.24 (q, -*C*O(O)R), 143.59 (t, 1-C), 135.71 (2C), 135.67 (2C), 133.68, 133.65 (Ar-C), 133.22 (q, 8-C), 129.87, 129.85 (Ar-C), 129.32 (t, 9-C), 128.76 (t, 2-C), 127.85 (4C, Ar-C), 97.12 (s, $-OCH_2OCH_3$), 96.88 (s, $-OCH_2OCH_3$), 96.78 (s, $-OCH_2OCH_3$), 96.55 (s, $-OCH_2OCH_3$), 77.36 (t, 14-C), 76.99 (t, 12-C), 73.99 (t, 11-C), 71.02 (s, 29-C), 65.76 (s, 1’-C), 63.16 (s, 7-C), 56.08 (p, $-OCH_2OCH_3$), 55.61 (p, $-OCH_2OCH_3$), 55.59 (p, $-OCH_2OCH_3$), 55.54 (p, $-OCH_2OCH_3$), 48.97 (q, 4-C), 46.83 (t, 10-C), 40.36 (t, 5-C), 36.34 (t, 13-C), 32.70 (s, 6-C), 26.96 (p, 3C, $-SiC(CH_3)_3$), 21.01 (p, 28-C), 19.26 (q, $-SiC(CH_3)_3$), 11.42 (p, 18-C) ppm; **HRMS-ESI** ($C_{43}H_{62}O_{12}Si$): calc. for $[M+H]^+$: 799.40833, found: 799.40954.

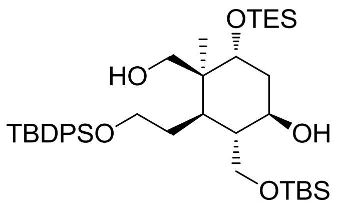


**S36**

**Diol S36.** To a solution of alcohol **91** (270 mg, 0.37 mmol) in THF (12 ml) was added $LiBH_4$ (4 M in THF, 0.28 ml, 1.11 mmol). The mixture was heated at reflux overnight. After cooling to room temperature aqueous saturated $NH_4Cl$-solution (20 ml) was added. The aqueous phase was extracted with EtOAc (3 × 20 ml). The combined organic layers were washed with brine (50 ml), dried ($MgSO_4$) and concentrated under reduced pressure. Purification by flash chromatography on silica gel with petroleum ether/EtOAc 4:1 as eluent gave diol **S36** (218 mg, 84%) as a colourless oil.

$\mathbf{R_f}$ = 0.24 (petroleum ether:EtOAc = 4:1); $[\alpha]_D^{20}$ = −9.4 (*c* = 1.0 in $CHCl_3$); **$^1$H NMR** (400 MHz, $C_6D_6$) δ = 7.85 – 7.79 (m, 4H, Ar-H), 7.30 – 7.22 (m, 6H, Ar-H), 4.18 (td, *J* = 10.9, 5.2 Hz, 1H, 1-H), 4.10 (s, 1H, 3-H), 4.07 (dd, *J* = 10.1, 2.7 Hz, 1H, -C$H_2$OTBS), 3.83 (td, *J* = 9.7, 5.8 Hz, 1H, 7-$H_a$), 3.68 (td, *J* = 9.4, 6.7 Hz, 1H, 7-$H_b$), 3.58 – 3.49 (m, 2H, 29-$H_a$, -C$H_2$OTBS), 3.27 (d, *J* = 10.6 Hz, 1H, 29-$H_b$), 2.08 (dt, *J* = 13.4, 4.5 Hz, 1H, 2-$H_a$), 1.96 – 1.80 (m, 2H, 2-$H_b$, 6-$H_a$), 1.71 (ddd, *J* = 11.9, 5.2, 2.8 Hz, 1H, 5-H), 1.54 – 1.41 (m, 2H, 6-$H_b$, 10-H), 1.20 (s, 9H, -SiC(C$H_3$)$_3$), 1.06 (s, 3H, -C$H_3$), 1.00 (t, *J* = 7.9 Hz, 9H, -Si(CH$_2$C$H_3$)$_3$), 0.92 (s, 9H, -SiC(C$H_3$)$_3$), 0.68 – 0.54 (m, 6H, -Si(C$H_2$CH$_3$)$_3$), 0.04 (s, 3H, -Si(C$H_3$)$_2$), -0.02 (s, 3H, -Si(C$H_3$)$_2$) ppm; **$^{13}$C NMR** (101 MHz, $C_6D_6$) δ = 136.04 (2C), 136.03 (2C), 134.43, 134.38, 130.01 (2C), 128.13 (2C), 128.11 (2C) (Ar-C), 73.12 (t, 3-C), 69.01 (t, 1-C), 65.48 (s, 7-C), 65.31, 65.27 (29-C, -*C*H$_2$OTBS), 48.36 (t, 10-C), 43.20 (q, 4-C), 37.96 (s, 2-C), 34.70 (t, 5-C), 32.63 (s, 6-C), 27.21 (p, 3C, -SiC(*C*H$_3$)$_3$), 26.11 (p, 3C, -SiC(*C*H$_3$)$_3$), 20.91 (p, -*C*H$_3$), 19.49 (q, -Si*C*(CH$_3$)$_3$), 18.35 (q, -Si*C*(CH$_3$)$_3$), 7.38 (p, 3C, -Si(CH$_2$*C*H$_3$)$_3$), 5.45 (s, 3C, -Si(*C*H$_2$CH$_3$)$_3$), -5.40 (p, -Si(*C*H$_3$)$_2$), -5.45 (p, -Si(*C*H$_3$)$_2$) ppm; **HRMS-ESI** ($C_{39}H_{68}O_5Si_3$): calc. for $[M+H]^+$: 701.44473, found: 701.44552.

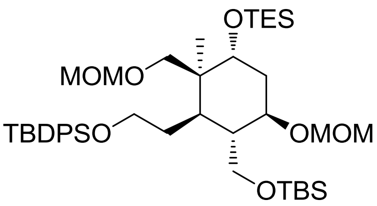


**S37**

***Bis*-MOM ether S37.** To a solution of diol **S36** (326 mg, 0.46 mmol) in THF (25 ml) were added DIPEA (2.43 ml, 14.0 mmol), MOMCl (0.71 ml, 9.30 mmol) and NaI (348 mg, 2.32 mmol). The mixture was stirred at 50 °C in a sealed tube overnight. The reaction was quenched by the addition of water (50 ml) and EtOAc (50 ml). The resulting layers were separated and the aqueous phase was extracted with EtOAc (2 × 50 ml). The combined organic layers were washed with brine (50 ml), dried ($MgSO_4$) and concentrated under reduced pressure. Purification by flash chromatography on silica gel with petroleum ether/EtOAc 30:1 as eluent gave *bis*-MOM ether **S37** (327 mg, 89%) as a colourless oil.

$\mathbf{R_f}$ = 0.26 (petroleum ether:EtOAc = 20:1); $[\alpha]_D^{20}$ = −2.5 (*c* = 1.0 in $CHCl_3$); **$^1$H NMR** (400 MHz, $C_6D_6$) δ = 7.88 – 7.81 (m, 4H, Ar-H), 7.31 – 7.21 (m, 6H, Ar-H), 4.81 (d, *J* = 6.5 Hz, 1H, -OC$H_2$OC$H_3$), 4.69 (d, *J* = 6.5 Hz, 1H, -OC*H*$_2$OCH$_3$), 4.41 (s, 2H, -OC*H*$_2$OCH$_3$), 4.16 (s, 1H, 3-H), 4.11 (td, *J* = 11.2, 5.2 Hz, 1H, 1-H), 4.03 (td, *J* = 10.3, 5.3 Hz, 1H, 7-$H_a$), 3.97 (dd, *J* = 9.9, 2.0 Hz, 1H, -C*H*$_2$OTBS), 3.82 (td, *J* = 10.4, 6.3 Hz, 1H, 7-$H_b$), 3.65 (d, *J* = 9.7 Hz, 1H, 29-$H_a$), 3.58 (d, *J* = 9.9 Hz, 1H, -C*H*$_2$OTBS), 3.39 (d, *J* = 9.6 Hz, 1H, 29-$H_b$), 3.25 (s, 3H, -OCH$_2$OC*H*$_3$), 3.17 (s, 3H, -OCH$_2$OC*H*$_3$), 2.37 (dt, *J* = 13.0, 4.6 Hz, 1H, 2-$H_a$), 2.05 – 1.91 (m, 3H, 2-$H_b$, 5-H, 6-$H_a$), 1.62 (qd, *J* = 10.8, 5.3 Hz, 1H, 6-$H_b$), 1.49 (t, *J* = 11.2 Hz, 1H, 10-H), 1.22 (s, 9H, -SiC(C*H*$_3$)$_3$), 1.14 (s, 3H, -C*H*$_3$), 1.04 (t, *J* = 7.9 Hz, 9H, -Si(CH$_2$C*H*$_3$)$_3$), 0.94 (s, 9H, -SiC(C*H*$_3$)$_3$), 0.74 – 0.56 (m, 6H, -Si(C*H*$_2$CH$_3$)$_3$), 0.03 (s, 3H, -Si(C*H*$_3$)$_2$), -0.04 (s, 3H, -Si(C*H*$_3$)$_2$) ppm; **$^{13}$C NMR** (101 MHz, $C_6D_6$) δ = 136.13 (2C), 136.10 (2C), 134.73, 134.65, 129.94 (2C), 128.20 (4C) (Ar-C), 97.57 (s, -O*C*H$_2$OCH$_3$), 97.00 (s, -O*C*H$_2$OCH$_3$), 73.98 (t, 1-C), 73.48 (t, 3-C), 71.17 (s, 29-C), 65.70 (s, 7-C), 59.09 (s, -*C*H$_2$OTBS), 55.14 (p, -OCH$_2$O*C*H$_3$), 55.04 (p, -OCH$_2$O*C*H$_3$), 47.15 (t, 10-C), 42.33 (q, 4-C), 36.29 (s, 2-C), 34.40 (t, 5-C), 32.75 (s, 6-C), 27.22 (p, 3C, -SiC(*C*H$_3$)$_3$), 26.21 (p, 3C, -SiC(*C*H$_3$)$_3$), 21.74 (p, -*C*H$_3$), 19.47 (q, -Si*C*(CH$_3$)$_3$), 18.33 (q, -Si*C*(CH$_3$)$_3$), 7.39 (p, 3C, -Si(CH$_2$*C*H$_3$)$_3$), 5.41 (s, 3C, -Si(*C*H$_2$CH$_3$)$_3$), -5.29 (p, -Si(*C*H$_3$)$_2$), -5.46 (p, -Si(*C*H$_3$)$_2$) ppm; **HRMS-ESI** ($C_{43}H_{76}O_7Si_3$): calc. for $[M+H]^+$: 789.49716, found: 789.49751.

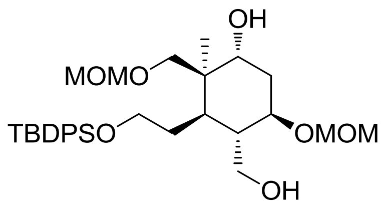


**S38**

**Diol S38**. *Bis*-MOM ether **S37** (547 mg, 0.69 mmol) was stirred in a solution of 8% TFA in $CH_2Cl_2$ (5.5 ml) at room temperature. After 30 min the reaction was quenched by the addition of aqueous saturated $NaHCO_3$-solution (20 ml). The aqueous phase was extracted with $CH_2Cl_2$ (2 × 30 ml). The combined organic layers were washed with brine (40 ml), dried ($MgSO_4$) and concentrated in vacuo. Purification by flash chromatography on silica gel with petroleum ether/EtOAc 1:1 as eluent gave diol **S38** (202 mg, 52%) as a colourless oil.

$\mathbf{R_f}$ = 0.29 (petroleum ether:EtOAc = 1:1); $[\boldsymbol{\alpha}]_{\mathbf{D}}^{\mathbf{20}}$ = −23.6 (*c* = 1.0 in $CHCl_3$); **$^{1}$H NMR** (400 MHz, $C_6D_6$) δ = 7.88 – 7.79 (m, 4H, Ar-H), 7.30 – 7.20 (m, 6H, Ar-H), 4.57 (d, *J* = 6.5 Hz, 1H, -OC$H_2$OCH$_3$), 4.52 (d, *J* = 6.5 Hz, 1H, -OC$H_2$OCH$_3$), 4.36 (s, 2H, -OC$H_2$OCH$_3$), 4.12 (td, *J* = 11.1, 5.0 Hz, 1H, 1-H), 4.00 (dd, *J* = 11.5, 1.9 Hz, 1H, -C$H_2$OH), 3.83 (dd, *J* = 13.2, 4.7 Hz, 2H, 3-H, 7-$H_a$), 3.77 – 3.67 (m, 1H, 7-$H_b$), 3.55 (dd, *J* = 11.6, 3.2 Hz, 1H, -C$H_2$OH), 3.49 (d, *J* = 9.8 Hz, 1H, 29-$H_b$), 3.22 (d, *J* = 9.7 Hz, 1H, 29-$H_b$), 3.13 (s, 3H, -OCH$_2$OC$H_3$), 3.11 (s, 3H, -OCH$_2$OC$H_3$), 2.13 – 2.04 (m, 1H, 2-$H_a$), 1.92 (ddd, *J* = 12.1, 5.2, 3.2 Hz, 1H, 5-H), 1.87 – 1.78 (m, 1H, 2-$H_b$), 1.78 – 1.68 (m, 1H, 6-$H_a$), 1.50 – 1.38 (m, 2H, 6-$H_b$, 10-H), 1.21 (s, 9H, -SiC(C$H_3$)$_3$), 1.00 (s, 3H, -C$H_3$) ppm; **$^{13}$C NMR** (101 MHz, $C_6D_6$) δ = 136.19 (2C), 136.16 (2C), 134.25, 134.21, 130.02, 130.01, 128.14 (2C), 128.13 (2C) (Ar-C), 96.88 (s, -O*C*H$_2$OCH$_3$), 96.13 (s, -O*C*H$_2$OCH$_3$), 73.78 (t, 1-C), 72.34 (t, 3-H), 70.97 (s, 29-C), 65.56 (s, 7-C), 60.06 (s, -*C*H$_2$OH), 55.32 (p, -OCH$_2$O*C*H$_3$), 55.01 (p, -OCH$_2$O*C*H$_3$), 46.75 (t, 10-C), 41.91 (q, 4-C), 35.31 (s, 2-C), 34.36 (t, 5-C), 31.41 (s, 6-C), 27.23 (p, 3C, -SiC(*C*H$_3$)$_3$), 20.94 (p, -*C*H$_3$), 19.47 (q, -Si*C*(CH$_3$)$_3$) ppm; **HRMS-ESI** ($C_{31}H_{48}O_7Si$): calc. for $[M+H]^+$: 561.32421, found: 561.32479.

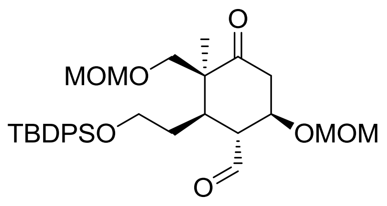


**S39**

**Aldehyde S49.** To a solution of diol **S38** (201 mg, 0.36 mmol) and $NaHCO_3$ (301 mg, 3.58 mmol) in $CH_2Cl_2$ (35 ml) was added Dess–Martin periodinane (608 mg, 1.43 mmol) at 0 °C. The reaction mixture was stirred for 3 h at 0 °C. Aqueous $Na_2S_2O_3$-solution (50 ml) was added and the aqueous phase was extracted with $CH_2Cl_2$ (2 × 40 ml). The combined organic layers were washed with brine (50 ml), dried ($MgSO_4$) and concentrated under reduced pressure. Purification by flash chromatography on silica gel with petroleum ether/EtOAc 6:1 as eluent gave aldehyde **S39** (172 mg, 86%) as a colourless oil.

$\mathbf{R}_f$ = 0.53 (petroleum ether:EtOAc = 2:1); $[\alpha]_D^{20}$ = −53.6 (*c* = 1.0 in $CHCl_3$); **$^1$H NMR** (400 MHz, $C_6D_6$) δ = 9.28 (d, *J* = 4.4 Hz, 1H, -C*H*O), 7.79 – 7.70 (m, 4H, Ar-H), 7.31 – 7.21 (m, 6H, Ar-H), 4.31 (d, *J* = 7.0 Hz, 1H, -OC$H_2$OC$H_3$), 4.21 (d, *J* = 7.0 Hz, 1H, -OC$H_2$OC$H_3$), 4.16 (s, 2H, -OC$H_2$OC$H_3$), 3.80 (td, *J* = 10.4, 5.2 Hz, 1H, 1-H), 3.58 (ddd, *J* = 10.6, 7.3, 5.0 Hz, 1H, 7-$H_a$), 3.52 – 3.44 (m, 2H, 7-$H_b$, 29-$H_a$), 3.19 (d, *J* = 9.8 Hz, 1H, 29-$H_b$), 3.02 (s, 3H, -$OCH_2OCH_3$), 3.01 (s, 3H, -$OCH_2OCH_3$), 2.97 – 2.88 (m, 2H, 2-$H_a$, 10-H), 2.70 (dd, *J* = 13.2, 11.1 Hz, 1H, 2-$H_b$), 1.75 – 1.65 (m, 1H, 6-$H_a$), 1.62 – 1.46 (m, 2H, 5-H, 6-$H_b$), 1.15 (s, 9H, -SiC$(CH_3)_3$), 1.09 (s, 3H, -$CH_3$) ppm; **$^{13}$C NMR** (101 MHz, $C_6D_6$) δ = 207.32 (q, 3-C), 201.96 (t, -*C*HO), 136.08 (2C), 136.05 (2C), 134.00, 133.84, 130.24, 130.20, 128.23 (4C) (Ar-C), 96.58 (s, -O*C*$H_2OCH_3$), 95.19 (s, -O*C*$H_2OCH_3$), 73.48 (t, 1-C), 71.01 (s, 29-C), 63.15 (s, 7-C), 58.55 (t, 10-C), 55.45 (p, -$OCH_2OCH_3$), 55.31 (p, -$OCH_2OCH_3$), 51.74 (q, 4-C), 45.01 (s, 2-C), 38.00 (t, 5-C), 31.69 (s, 6-C), 27.16 (p, 3C, -SiC$(CH_3)_3$), 19.40 (p, -$CH_3$), 19.14 (q, -Si*C*$(CH_3)_3$) ppm; **HRMS-ESI** ($C_{31}H_{44}O_7Si$): calc. for $[M+Na]^+$: 579.27485, found: 579.27442.

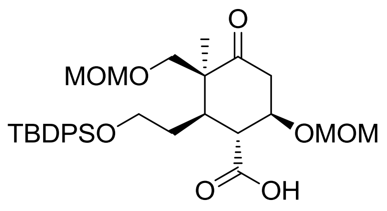

**92**

**Carboxylic acid 92.** To a solution of aldehyde **S39** (146 mg, 0.26 mmol) in *t*-BuOH (14 ml) and water (3.5 ml) were added 2-methyl-2-butene (0.56 ml, 5.24 mmol), $NaH_2PO_4$ (126 mg, 1.05 mmol) and $NaClO_2$ (80%, 148 mg, 1.31 mmol) at 0 °C. After 10 min, the mixture was allowed to warm to room temperature and stirred for 2.5 h. The reaction was quenched by the addition of aqueous saturated $NH_4Cl$-solution (15 ml). The aqueous phase was extracted with EtOAc (3 × 15 ml) and the combined organic layers were washed with brine (30 ml), dried ($MgSO_4$) and concentrated under reduced pressure. Purification by flash chromatography on silica gel (petroleum ether/EtOAc 2:1, 0.1% acetic acid) gave carboxylic acid **92** (149 mg, 99%) as a colourless oil.

$\mathbf{R_f}$ = 0.22 (petroleum ether:EtOAc = 1:1); $[\boldsymbol{\alpha}]_{\mathbf{D}}^{\mathbf{20}}$ = −48.9 (*c* = 1.0 in $CHCl_3$); **$^{1}$H NMR** (400 MHz, $C_6D_6$) δ = 7.82 – 7.73 (m, 4H, Ar-H), 7.32 – 7.22 (m, 6H, Ar-H), 4.42 (s, 2H, -OC$H_2$OCH$_3$), 4.15 (d, *J* = 6.6 Hz, 1H, -OC$H_2$OCH$_3$), 4.13 (d, *J* = 6.5 Hz, 1H, -OC$H_2$OCH$_3$), 3.98 (td, *J* = 10.8, 5.1 Hz, 1H, 1-H), 3.80 – 3.62 (m, 2H, 7-H), 3.49 (d, *J* = 9.7 Hz, 1H, 29-$H_a$), 3.26 (t, *J* = 11.0 Hz, 1H, 10-H), 3.19 (d, *J* = 9.8 Hz, 1H, 29-$H_b$), 3.12 (s, 3H, -OCH$_2$OC$H_3$), 3.06 (dd, *J* = 13.5, 5.2 Hz, 1H, 2-$H_a$), 3.01 (s, 3H, -OCH$_2$OC$H_3$), 2.77 (dd, *J* = 13.4, 11.6 Hz, 1H, 2-$H_b$), 1.92 – 1.81 (m, 2H, 5-H, 6-$H_a$), 1.77 – 1.66 (m, 1H, 6-$H_b$), 1.16 (s, 9H, -SiC(C$H_3$)$_3$), 1.11 (s, 3H, -C$H_3$) ppm; **$^{13}$C NMR** (101 MHz, $C_6D_6$) δ = 207.71 (q, 3-C), 178.71 (q, -*C*O$_2$H), 136.09 (2C), 136.04 (2C), 134.07 (2C), 130.11 (2C), 128.22 (2C), 128.20 (2C) (Ar-C), 96.49 (s, -O*C*H$_2$OCH$_3$), 96.05 (s, -O*C*H$_2$OCH$_3$), 76.41 (t, 1-C), 71.25 (s, 29-C), 63.28 (s, 7-C), 55.45 (p, -OCH$_2$O*C*H$_3$), 55.30 (p, -OCH$_2$O*C*H$_3$), 52.97 (t, 10-C), 51.82 (q, 4-C), 46.21 (s, 2-C), 40.36 (t, 5-C), 32.48 (s, 6-C), 27.12 (p, 3C, -SiC(*C*H$_3$)$_3$), 19.44 (p, -*C*H$_3$), 19.20 (q, -Si*C*(CH$_3$)$_3$) ppm; **HRMS-ESI** ($C_{31}H_{44}O_8Si$): calc. for $[M+Na]^+$: 595.26977, found: 579.26920.

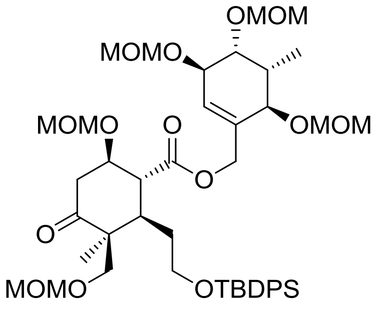

**93**

**Ester 93**. To a solution of carboxylic acid **92** (38 mg, 66.3 µmol) in $CH_2Cl_2$ (0.5 ml) was added EDC·HCl (28 mg, 0.15 mmol) and DMAP (9 mg, 73.0 µmol). After stirring for 30 min a solution of alcohol **40** (20 mg, 66.3 µmol) in $CH_2Cl_2$ (0.5 ml) was added. The reaction mixture was stirred at room temperature for 2 days. EtOAc (10 ml) was added and the organic layer was washed with 1 M HCl (10 ml), aqueous saturated $NaHCO_3$-solution (10 ml), water (10 ml) and brine (10 ml), dried ($MgSO_4$) and evaporated under reduced pressure. Purification by flash chromatography on silica gel with petroleum ether/EtOAc 4:1 to 2:1 as eluent gave ester **93** (27 mg, 47%) as a colourless oil.

$\mathbf{R_f}$ = 0.39 (petroleum ether:EtOAc = 1:1); $[\alpha]_D^{20}$ = −13.0 (*c* = 1.0 in $CHCl_3$); **$^1$H NMR** (400 MHz, $CDCl_3$) δ = 7.66 – 7.58 (m, 4H, Ar-H), 7.45 – 7.34 (m, 6H, Ar-H), 5.73 – 5.69 (m, 1H, 9-H), 4.78 – 4.48 (m, 11H, 30-$H_a$, -OC$H_2$OC$H_3$), 4.29 (d, *J* = 13.1 Hz, 1H, 30-$H_b$), 4.01 (d, *J* = 7.9 Hz, 1H, 11-H), 3.98 – 3.88 (m, 2H, 1-H, 12-H), 3.82 – 3.73 (m, 2H, 14-H, 29-$H_a$), 3.55 (ddd, *J* = 10.6, 7.9, 4.4 Hz, 1H, 7-Ha), 3.40 – 3.38 (m, 1H, 7-$H_b$), 3.37 (s, 3H, -OC$H_2$OC$H_3$), 3.37 (s, 3H, -OC$H_2$OC$H_3$), 3.36 (s, 3H, -OC$H_2$OC$H_3$), 3.33 – 3.32 (m, 1H, 29-$H_b$), 3.31 (s, 3H, -OC$H_2$OC$H_3$), 3.29 (s, 3H, -OC$H_2$OC$H_3$), 3.06 (dd, *J* = 11.8, 10.5 Hz, 1H, 10-H), 2.94 (dd, *J* = 13.4, 5.1 Hz, 1H, 2-$H_a$), 2.72 (dd, *J* = 13.3, 11.9 Hz, 1H, 2-$H_b$), 2.41 (ddq, *J* = 10.9, 7.2, 3.7 Hz, 1H, 13-H), 1.94 – 1.83 (m, 1H, 6-$H_a$), 1.80 (ddd, *J* = 11.8, 6.7, 2.4 Hz, 1H, 5-H), 1.61 (dtd, *J* = 11.2, 6.7, 4.7 Hz, 1H, 6-$H_b$), 1.03 (s, 3H, 28-H), 1.02 (s, 9H, -SiC(C$H_3$)$_3$), 0.92 (d, *J* = 7.3 Hz, 3H, 18-H) ppm; **$^{13}$C NMR** (101 MHz, $CDCl_3$) δ = 209.33 (q, 3-C), 173.64 (q, -*C*O(O)R), 135.75 (2C), 135.62 (2C), 133.68, 133.61 (Ar-C), 133.44 (q, 8-C), 129.85, 129.83 (Ar-C), 128.33 (t, 9-C), 127.86 (2C), 127.85 (2C) (Ar-C), 97.28 (s, -O*C*$H_2$OC$H_3$), 96.77 (s, -O*C*$H_2$OC$H_3$), 96.63 (s, -O*C*$H_2$OC$H_3$), 96.50 (s, -O*C*$H_2$OC$H_3$), 96.19 (s, -O*C*$H_2$OC$H_3$), 77.47 (t, 14-C), 77.07 (t, 12-C), 76.27 (t, 1-C), 73.75 (t, 11-C), 71.11 (s, 29-C), 65.11 (s, 1'-C), 62.68 (s, 7-C), 55.96 (p, -OC$H_2$O*C*$H_3$), 55.80 (p, -OC$H_2$O*C*$H_3$), 55.76 (p, -OC$H_2$O*C*$H_3$), 55.58 (p, -OC$H_2$O*C*$H_3$), 55.55 (p, -OC$H_2$O*C*$H_3$), 52.86 (t, 10-C), 51.89 (q, 4-C), 45.94 (s, 2-C), 40.42 (t, 5-C), 36.48 (t, 13-C), 32.45 (s, 6-C),

26.93 (p, 3C, -SiC(*C*$H_3$)$_3$), 19.27 (q, -Si*C*($CH_3$)$_3$), 18.63 (p, 28-C), 11.40 (p, 18-C) ppm; **HRMS-ESI** ($C_{45}H_{68}O_{14}Si$): calc. for $[M+H]^+$: 861.44511, found: 861.44565.

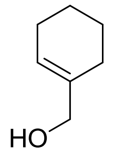


**S40**

**Cyclohexenylmethanol S40.** A solution of 1-cyclohexene-1-carboxcylic acid (555 mg, 4.40 mmol) in $Et_2O$ (1.5 ml) was added dropwise to a solution of $LiAlH_4$ (217 mg, 5.72 mmol) in $Et_2O$ (10 ml). The resulting suspension was stirred at room temperature for 1.5 h. Water was added and the mixture was filtered. The aqueous phase was extracted with $EtO_2$. The combined organic layers were washed with brine, dried ($MgSO_4$) and concentrated in vacuo. Purification of the crude product by flash chromatography on silica gel with petroleum ether/EtOAc 4:1 as eluent gave compound **S40** (280 mg, 57%) as a colourless oil.

Spectroscopic data are identical to those reported [2]: **$^1$H NMR** (400 MHz, CDCl3) δ = 5.72 – 5.63 (m, 1H), 4.00 – 3.93 (m, 2H), 2.07 – 1.96 (m, 4H), 1.70 – 1.54 (m, 4H), 1.47 (bs, 1H); **$^{13}$C NMR** (101 MHz, $CDCl_3$) δ = 137.67, 123.19, 67.84, 25.76, 25.06, 22.68, 22.58.

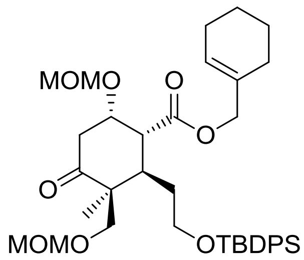


**95**

**Ester 95**. To a solution of carboxylic acid **87** (39 mg, 68.1 µmol) in $CH_2Cl_2$ (0.5 ml) was added EDC·HCl (29 mg, 0.15 mmol) and DMAP (9 mg, 74.9 µmol). After stirring for 30 min a solution of cyclohexenylmethanol **S40** (8.4 mg, 74.9 µmol) in $CH_2Cl_2$ (0.5 ml) was added. The reaction mixture was stirred at room temperature overnight. EtOAc (10 ml) was added and the organic layer was washed with 1 M HCl (10 ml), aqueous saturated $NaHCO_3$-solution (10 ml), water (10 ml) and brine (10 ml), dried ($MgSO_4$) and evaporated under reduced

pressure. Purification by flash chromatography on silica gel with petroleum ether/EtOAc 6:1 as eluent gave ester **95** (34 mg, 75%) as a colourless oil.

**R$_f$** = 0.31 (petroleum ether:EtOAc = 4:1); $[\alpha]_D^{20}$ = −8.7 (*c* = 1.0 in $CHCl_3$); **$^1$H NMR** (400 MHz, $CDCl_3$) δ = 7.69 – 7.62 (m, 4H, Ar-H), 7.44 – 7.33 (m, 6H, Ar-H), 5.72 (s, 1H, 9-H), 4.58 (d, *J* = 7.1 Hz, 1H, -OC*H*$_2$OCH$_3$), 4.49 (s, 2H, -OC*H*$_2$OCH$_3$), 4.46 (d, *J* = 7.2 Hz, 1H, -OC*H*$_2$OCH$_3$), 4.43 – 4.32 (m, 3H, 1-H, 30-H), 3.87 (td, *J* = 10.2, 5.6 Hz, 1H, 7-H$_a$), 3.67 – 3.57 (m, 2H, 7-H$_b$, 29-H$_a$), 3.28 (s, 3H, -OCH$_2$OC*H*$_3$), 3.27 (s, 3H, -OCH$_2$OC*H*$_3$), 3.27 – 3.26 (m, 1H, 29-H$_b$), 3.14 (dd, *J* = 12.1, 2.9 Hz, 1H, 10-H), 2.75 (qd, *J* = 14.3, 3.5 Hz, 2H, 2-H), 2.20 (dt, *J* = 11.9, 4.6 Hz, 1H, 5-H), 2.06 – 1.99 (m, 2H, cyclohexyl), 1.99 – 1.92 (m, 2H, cyclohexyl), 1.83 (ddd, *J* = 15.7, 10.8, 5.5 Hz, 1H, 6-H$_a$), 1.68 – 1.60 (m, 2H, cyclohexyl), 1.60 – 1.47 (m, 3H, 6-H$_b$, cyclohexyl), 1.04 (s, 9H, -SiC(C*H*$_3$)$_3$), 0.89 (s, 3H, 28-H) ppm; **$^{13}$C NMR** (101 MHz, $CDCl_3$) δ = 210.68 (q, 3-C), 172.18 (q, -*C*(O)OR), 135.76 (2C), 135.71 (2C), 134.16, 134.06 (Ar-C), 132.77 (q, 8-C), 129.76, 129.72, 127.79 (4C) (Ar-C), 127.23 (t, 9-C), 96.78 (s, -O*C*H$_2$OCH$_3$), 95.44 (s, -O*C*H$_2$OCH$_3$), 75.09 (t, 1-C), 71.85 (s, 29-C), 69.42 (s, 30-C), 64.15 (s, 7-C), 55.86 (p, -OCH$_2$O*C*H$_3$), 55.83 (p, -OCH$_2$O*C*H$_3$), 51.73 (q, 4-C), 50.68 (t, 10-C), 44.29 (s, 2-C), 38.49 (t, 5-C), 33.70 (s, 6-C), 27.05 (p, 3C, -SiC(*C*H$_3$)$_3$), 26.17 (s, cyclohexyl), 25.17 (s, cyclohexyl), 22.52 (s, cyclohexyl), 22.20 (s, cyclohexyl), 19.30 (q, -Si*C*(CH$_3$)$_3$), 19.02 (p, 28-C) ppm; **HRMS-ESI** ($C_{38}H_{54}O_8Si$): calc. for $[M+H]^+$: 667.36607, found: 667.36665.

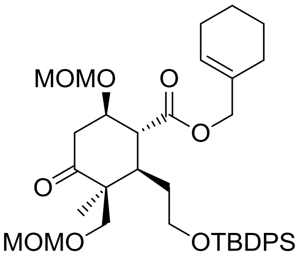


**96**

**Ester 96**. To a solution of carboxylic acid **92** (20 mg, 34.9 µmol) in $CH_2Cl_2$ (0.5 ml) was added EDC·HCl (15 mg, 76.8 µmol) and DMAP (5 mg, 38.4 µmol). After stirring for 30 min a solution of cyclohexenylmethanol **S40** (3.5 mg, 31.4 µmol) in $CH_2Cl_2$ (0.5 ml) was added. The reaction mixture was stirred at room temperature overnight. EtOAc (10 ml) was added and the organic layer was washed with 1 M HCl (10 ml), aqueous saturated $NaHCO_3$-solution (10 ml), water (10 ml) and brine (10 ml), dried ($MgSO_4$) and evaporated under reduced

pressure. Purification by flash chromatography on silica gel with petroleum ether/EtOAc 6:1 as eluent gave ester **96** (11 mg, 53%) as a colourless oil.

**R$_f$** = 0.33 (petroleum ether:EtOAc = 4:1); $[\alpha]_D^{20}$ = −25.1 (*c* = 1.0 in $CHCl_3$); **$^1$H NMR** (400 MHz, $CDCl_3$) δ = 7.66 – 7.59 (m, 4H, Ar-H), 7.45 – 7.33 (m, 6H, Ar-H), 5.63 (s, 1H, 9-H), 4.59 (d, *J* = 6.9 Hz, 1H, -OC$H_2$OCH$_3$), 4.56 (d, *J* = 6.8 Hz, 1H, -OC$H_2$OCH$_3$), 4.52 (s, 2H, -OC$H_2$OCH$_3$), 4.29 (s, 2H, 30-H), 3.92 (ddd, *J* = 11.6, 10.5, 5.1 Hz, 1H, 1-H), 3.77 (d, *J* = 9.7 Hz, 1H, 29-H$_a$), 3.55 (ddd, *J* = 10.2, 8.6, 4.7 Hz, 1H, 7-H$_a$), 3.40 (dt, *J* = 10.2, 7.7 Hz, 1H, 7-H$_b$), 3.33 (d, *J* = 9.7 Hz, 1H, 29-H$_b$), 3.31 (s, 3H, -OCH$_2$OC$H_3$), 3.29 (s, 3H, -OCH$_2$OC$H_3$), 3.04 (dd, *J* = 11.8, 10.5 Hz, 1H, 10-H), 2.91 (dd, *J* = 13.4, 5.2 Hz, 1H, 2-H$_a$), 2.72 (dd, *J* = 13.3, 11.8 Hz, 1H, 2-H$_b$), 2.00 – 1.92 (m, 2H, cyclohexyl), 1.92 – 1.82 (m, 3H, 6-H$_a$, cyclohexyl), 1.74 (ddd, *J* = 11.7, 6.6, 2.4 Hz, 1H, 5-H), 1.69 – 1.62 (m, 1H, 6-H$_b$), 1.62 – 1.48 (m, 4H, cyclohexyl), 1.02 (s, 9H, -SiC(C$H_3$)$_3$), 1.01 (s, 3H, 28-H) ppm; **$^{13}$C NMR** (101 MHz, $CDCl_3$) δ = 209.37 (q, 3-C), 173.67 (q, -*C*(O)OR), 135.76 (2C), 135.65 (2C), 133.76, 133.72 (Ar-C), 132.47 (q, 8-C), 129.82, 129.80, 127.84 (4C) (Ar-C), 127.11 (t, 9-C), 96.77 (s, -O*C*H$_2$OCH$_3$), 96.14 (s, -O*C*H$_2$OCH$_3$), 76.26 (t, 1-C), 71.08 (s, 29-C), 69.49 (s, 30-C), 62.93 (s, 7-C), 55.80 (p, -OCH$_2$O*C*H$_3$), 55.78 (p, -OCH$_2$O*C*H$_3$), 53.18 (t, 10-C), 51.91 (q, 4-C), 45.95 (s, 2-C), 40.43 (t, 5-C), 32.46 (s, 6-C), 26.96 (p, 3C, -SiC(*C*H$_3$)$_3$), 26.04 (s, cyclohexyl), 25.10 (s, cyclohexyl), 22.46 (s, cyclohexyl), 22.16 (s, cyclohexyl), 19.27 (q, -Si*C*(CH$_3$)$_3$), 18.57 (p, 28-C) ppm; **HRMS-ESI** ($C_{38}H_{54}O_8Si$): calc. for $[M+H]^+$: 667.36607, found: 667.36669.

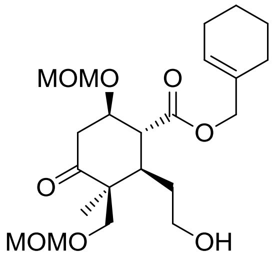


**S41**

**Alcohol S41**. To a solution of ester **96** (28 mg, 42.0 µmol) in DMF (1 ml) was added TAS-F (14 mg, 50.4 µmol) at 0 °C. The reaction mixture was stirred 2 h at 0 °C and 20 h at room temperature. EtOAc (10 ml) was added and the organic layer was washed with water (10 ml) and brine (10 ml), dried ($MgSO_4$) and evaporated under reduced pressure. Purification by

flash chromatography on silica gel with petroleum ether/EtOAc 2:1 as eluent gave alcohol **S41** (7 mg, 39%) as a colourless oil.

**R*f*** = 0.27 (petroleum ether:EtOAc = 1:1); **$^{1}$H NMR** (400 MHz, $C_6D_6$) δ = 5.70 – 5.65 (m, 1H, 9-H), 4.57 (d, *J* = 11.9 Hz, 1H, 30-$H_a$), 4.53 – 4.45 (m, 3H, -OC*H*$_2$OCH$_3$, 30-$H_b$), 4.16 (d, *J* = 6.6 Hz, 1H, -OC*H*$_2$OCH$_3$), 4.14 (d, *J* = 6.6 Hz, 1H, -OC*H*$_2$OCH$_3$), 4.07 (ddd, *J* = 11.7, 10.5, 5.1 Hz, 1H, 1-H), 3.53 (d, *J* = 9.7 Hz, 1H, 29-$H_a$), 3.42 (ddd, *J* = 10.8, 7.7, 5.2 Hz, 1H, 7-$H_a$), 3.36 – 3.28 (m, 1H, 7-$H_b$), 3.26 (dd, *J* = 11.9, 10.4 Hz, 1H, 10-H), 3.19 (d, *J* = 9.7 Hz, 1H, 29-$H_b$), 3.13 (s, 3H, -OCH$_2$OC*H*$_3$), 3.09 (dd, *J* = 13.1, 4.9 Hz, 1H, 2-$H_a$), 3.01 (s, 3H, -OCH$_2$OC*H*$_3$), 2.81 (dd, *J* = 13.2, 11.8 Hz, 1H, 2-$H_b$), 1.96 (s, 2H, cyclohexyl), 1.88 – 1.80 (m, 2H, cyclohexyl), 1.75 (ddd, *J* = 12.0, 6.7, 2.8 Hz, 1H, 5-H), 1.64 (dtd, *J* = 10.7, 7.9, 2.9 Hz, 1H, 6-$H_a$), 1.57 – 1.45 (m, 3H, 6-$H_b$, cyclohexyl), 1.44 – 1.37 (m, 2H, cyclohexyl), 1.11 (s, 3H, 28-H) ppm; **$^{13}$C NMR** (101 MHz, $C_6D_6$) δ = 207.54 (q, 3-C), 174.35 (q, -*C*(O)OR), 133.04 (q, 8-C), 127.18 (t, 9-C), 96.51 (s, -O*C*H$_2$OCH$_3$), 96.04 (s, -O*C*H$_2$OCH$_3$), 76.50 (t, 1-C), 71.23 (s, 29-C), 69.59 (s, 30-C), 61.69 (s, 7-C), 55.46 (p, -OCH$_2$O*C*H$_3$), 55.26 (p, -OCH$_2$O*C*H$_3$), 53.31 (t, 10-C), 51.89 (q, 4-C), 46.27 (s, 2-C), 40.85 (t, 5-C), 32.50 (s, 6-C), 26.27 (s, cyclohexyl), 25.30 (s, cyclohexyl), 22.72 (s, cyclohexyl), 22.39 (s, cyclohexyl), 18.86 (p, 28-C) ppm; **HRMS-ESI** ($C_{22}H_{36}O_8$): calc. for $[M+H]^+$: 429.24829, found: 429.24829.

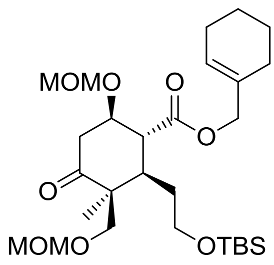


**97**

**TBS ether 97**. To a solution of alcohol **S41** (6 mg, 14.0 µmol) in DMF (1 ml) was added imidazole (3 mg, 42.0 µmol) and TBSCl (4 mg, 28.0 µmol). The reaction mixture was stirred at room temperature overnight. EtOAc (10 ml) and 1 M HCl (10 ml) were added and the layers were separated. The aqueous phase was extracted with EtOAc (2 × 10 ml). The combined organic layers were washed with saturated aqueous $NaHCO_3$-solution (20 ml) and brine (20 ml), dried ($MgSO_4$) and evaporated under reduced pressure. Purification by flash

chromatography on silica gel with petroleum ether/EtOAc 6:1 as eluent gave TBS-ether **97** (5 mg, 66%) as a colourless oil.

**R*f*** = 0.40 (petroleum ether:EtOAc = 4:1); **$^{1}$H NMR** (400 MHz, $C_6D_6$) δ = 5.74 – 5.67 (m, 1H), 4.60 (d, *J* = 12.1 Hz, 1H), 4.54 (d, *J* = 12.2 Hz, 1H), 4.50 (d, *J* = 6.8 Hz, 1H), 4.47 (d, *J* = 6.8 Hz, 1H), 4.16 (d, *J* = 6.6 Hz, 1H), 4.14 (d, *J* = 6.5 Hz, 1H), 4.13 – 4.05 (m, 1H), 3.63 (ddd, *J* = 10.0, 7.9, 4.6 Hz, 1H), 3.58 (d, *J* = 9.8 Hz, 1H), 3.48 (dd, *J* = 17.0, 8.0 Hz, 1H), 3.31 (dd, *J* = 11.7, 10.5 Hz, 1H), 3.23 (d, *J* = 9.7 Hz, 1H), 3.12 (s, 3H), 3.13 – 3.07 (m, 1H), 3.02 (s, 3H), 2.83 (dd, *J* = 13.2, 11.8 Hz, 1H), 2.01 – 1.93 (m, 2H), 1.90 – 1.79 (m, 4H), 1.74 – 1.64 (m, 1H), 1.55 – 1.46 (m, 2H), 1.45 – 1.37 (m, 2H), 1.21 (s, 3H), 0.98 (s, 9H), 0.11 (s, 3H), 0.10 (s, 3H) ppm; **$^{13}$C NMR** (101 MHz, $C_6D_6$) δ = 207.61, 173.84, 133.07, 126.89, 96.51, 96.14, 76.66, 71.25, 69.44, 62.58, 55.45, 55.25, 53.50, 51.96, 46.37, 40.75, 32.89, 26.28, 26.17, 25.31, 22.73, 22.43, 18.99, 18.48, -5.10, -5.13 ppm; **HRMS-ESI** ($C_{28}H_{50}O_8Si$): calc. for $[M+H]^+$: 543.33477, found: 543.33518.

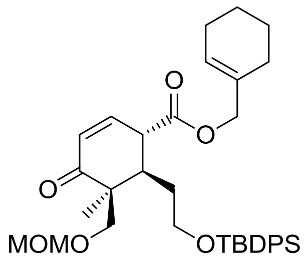


**98**

**Enone 98.** **R*f*** = 0.23 (petroleum ether:EtOAc = 9:1); $[\alpha]_D^{20}$ = +69.1 (*c* = 1.0 in $CHCl_3$); **$^{1}$H NMR** (400 MHz, $C_6D_6$) δ = 7.81 – 7.74 (m, 4H, Ar-H), 7.29 – 7.20 (m, 6H, Ar-H), 6.45 (dd, *J* = 10.2, 3.6 Hz, 1H, 1-H), 5.96 (dd, *J* = 10.2, 2.4 Hz, 1H, 2-H), 5.62 – 5.56 (m, 1H, 9-H), 4.45 – 4.35 (m, 2H, 30-H), 4.31 – 4.26 (m, 2H, $-OCH_2OCH_3$), 3.79 (ddd, *J* = 10.3, 7.5, 5.6 Hz, 1H, $7-H_a$), 3.67 (dt, *J* = 10.4, 7.1 Hz, 1H, $7-H_b$), 3.63 – 3.57 (m, 3H, 10-H, 29-H), 3.06 (s, 3H, $-OCH_2OCH_3$), 2.80 (ddd, *J* = 8.2, 6.5, 3.4 Hz, 1H, 5-H), 1.94 (dtd, *J* = 14.5, 7.4, 3.5 Hz, 1H, $6-H_a$), 1.88 – 1.78 (m, 4H, cyclohexyl), 1.62 – 1.51 (m, 1H, $6-H_b$), 1.51 – 1.41 (m, 2H, cyclohexyl), 1.42 – 1.33 (m, 2H, cyclohexyl), 1.20 (s, 3H, 28-H), 1.16 (s, 9H, $-SiC(CH_3)_3$) ppm; **$^{13}$C NMR** (101 MHz, $C_6D_6$) δ = 200.59 (q, 3-C), 172.41 (q, -*C*(O)OR), 143.42 (t, 1-C), 136.07 (2C), 136.02 (2C), 134.10 (2C) (Ar-C), 132.89 (q, 8-C), 130.07, 130.05 (Ar-C), 128.87 (t, 2-C), 128.15 (4C, Ar-C), 126.94 (t, 9-C), 96.74 (s, $-OCH_2OCH_3$), 71.35 (s, 29-C), 69.70 (s, 30-C), 63.58 (s, 7-C), 55.12 (p, $-OCH_2OCH_3$), 49.15 (q, 4-C), 47.07

(t, 10-C), 40.67 (t, 5-C), 32.97 (s, 6-C), 27.12 (p, 3C, -SiC(*C*$H_3$)$_3$), 26.20 (s, cyclohexyl), 25.24 (s, cyclohexyl), 22.68 (s, cyclohexyl), 22.36 (s, cyclohexyl), 21.18 (p, 28-C), 19.42 (q, -Si*C*($CH_3$)$_3$) ppm; **HRMS-ESI** ($C_{36}H_{48}O_6Si$): calc. for $[M+H]^+$: 605.32929, found: 605.32974.

## Abbreviations

AIBN = 2,2´-azo-bis*iso*butyronitrile, brsm = based on recovered starting material, CSA = camphorsulfonic acid, DCC = dicyclohexylcarbodiimide, DDQ = 2,3-Dichloro-5,6-dicyano-*p*-benzoquinone, DEAD = diethylazodicarboxylate, DIBAL-H = diisobutylaluminium hydride, DIPEA = diisopropylethylamine, DMAP = dimethylaminopyridine, DMF = dimethylformamide, DMP = Dess–Martin periodinane, DMPU = 1,3-dimethyl-3,4,5,6-tetrahydro-2(1*H*)-pyrimidinone, DMS = dimethylsulfide, DMSO = dimethylsulfoxide, EDC = 1-ethyl-3-(3-(dimethylamino)propyl)carbodiimide, HMPA = hexamethylphosphoramide, KHMDS = potassium bis(trimethylsilyl)amide, LiHMDS = lithium bis(trimethylsilyl)amide, MOM = methoxymethyl, Ms = methanesulfonyl, NBS = *N*-bromosuccinimide, Piv = pivaloyl, *p*TSA = *para*-toluenesulfonic acid, RCM = ring closing metathesis, rt = room temperature, TAS-F = Tris(dimethylamino)sulfonium difluorotrimethylsilicate, TBAF = tetrabutylammonium fluoride, TBDPS = *tert*-butyldiphenylsilyl, TBS = *tert*-butyldimethylsilyl, TES = triethylsilyl, Tf = triflouromethanesulfonyl, TFA = trifluoroacetic acid, THF = tetrahydrofuran, TIPS = triisopropylsilyl, TMS = trimethylsilyl.

## References

1. Schuster, H.; Martinez, R.; Bruss, H.; Antonchick, A. P.; Kaiser, M.; Schürmann, M.; Waldmann, H.; *Chem. Comm.* **2011**, *47*, 6545-6547.
2. Faulkner, A.; Scott, J. S.; Bower, J. F., *Chem Comm* **2013,** *49*, 1521-1523.
